# Empirical evidence about inconsistency among studies in a pair‐wise meta‐analysis

**DOI:** 10.1002/jrsm.1193

**Published:** 2015-12-17

**Authors:** Kirsty M. Rhodes, Rebecca M. Turner, Julian P. T. Higgins

**Affiliations:** ^1^ MRC Biostatistics UnitInstitute of Public HealthCambridgeUK; ^2^ School of Social and Community MedicineUniversity of BristolUK

**Keywords:** meta‐analysis, heterogeneity, inconsistency, intervention studies, Bayesian analysis

## Abstract

This paper investigates how inconsistency (as measured by the *I^2^* statistic) among studies in a meta‐analysis may differ, according to the type of outcome data and effect measure. We used hierarchical models to analyse data from 3873 binary, 5132 continuous and 880 mixed outcome meta‐analyses within the Cochrane Database of Systematic Reviews. Predictive distributions for inconsistency expected in future meta‐analyses were obtained, which can inform priors for between‐study variance. Inconsistency estimates were highest on average for binary outcome meta‐analyses of risk differences and continuous outcome meta‐analyses. For a planned binary outcome meta‐analysis in a general research setting, the predictive distribution for inconsistency among log odds ratios had median 22% and 95% CI: 12% to 39%. For a continuous outcome meta‐analysis, the predictive distribution for inconsistency among standardized mean differences had median 40% and 95% CI: 15% to 73%. Levels of inconsistency were similar for binary data measured by log odds ratios and log relative risks. Fitted distributions for inconsistency expected in continuous outcome meta‐analyses using mean differences were almost identical to those using standardized mean differences. The empirical evidence on inconsistency gives guidance on which outcome measures are most likely to be consistent in particular circumstances and facilitates Bayesian meta‐analysis with an informative prior for heterogeneity. © 2015 The Authors. *Research Synthesis Methods* published by John Wiley & Sons, Ltd. © 2015 The Authors. *Research Synthesis Methods* published by John Wiley & Sons, Ltd.

## 1 Introduction

A meta‐analysis combines results of multiple studies to summarize evidence in a specific research area. The combined results are reported with greater precision than the results of the individual studies, and so are more influential.

Variation among the results of individual studies in a meta‐analysis, known as heterogeneity, is inevitable when the studies have been conducted using various methods, at various times and by various research groups. We can allow for unexplained heterogeneity in a random‐effects meta‐analysis, estimating a between‐study variance and a summary effect (Higgins *et al*, 2009). Higgins and Thompson (2002) proposed *I* ^2^ as a statistic to measure the impact of between‐study heterogeneity. Throughout this paper, we refer to *I* ^2^ as a quantifier of inconsistency across results of included studies in a meta‐analysis, because it depends on the extent of overlap in confidence intervals across studies. The *I* ^2^ statistic directly relates to the between‐study heterogeneity variance *τ* ^2^ but has a similar interpretation regardless of the type of outcome data and the outcome metric used to perform the meta‐analysis. *I* ^2^ has an intuitive interpretation as the proportion of total variation in the estimates of intervention effect that is due to heterogeneity among studies. A number of papers have presented empirical investigations of heterogeneity between studies in meta‐analyses (Higgins and Whitehead, 1996; Engels *et al*, 2000; Deeks, 2002; Pullenayegum, 2011; Turner *et al*, 2012; Rhodes *et al*, 2015). However, despite extensive use of *I* ^2^ as a measure of the impact of heterogeneity, no large study has empirically examined typical values of inconsistency among results of included studies in a meta‐analysis.

An important consideration for meta‐analysis is the selection of a metric to measure the intervention effects of individual studies. When performing meta‐analysis of binary outcome data, Cochrane review authors may choose to use the relative risk scale for ease of interpretation (Sackett *et al*, 1996; Deeks, 1998). However, statisticians working in meta‐analysis often prefer to use the odds ratio scale for mathematical convenience (Senn, 1998; Olkin, 1998). For meta‐analysis of continuous outcome data, we typically use difference measures (mean difference or standardized mean difference [SMD]) for comparison of treatment arms. However, in principle, we could use relative effects by computing a ratio of mean values (RoM) (Friedrich *et al*, 2008). To date, little is known in regard to whether SMDs are more or less consistent than mean differences or whether difference measures exhibit lower or higher levels of inconsistency than relative measures.

Another important consideration for random‐effects meta‐analysis concerns the estimation of between‐study heterogeneity. Numerous meta‐analyses in healthcare research combine results from only a small number of studies, for which between‐study heterogeneity is estimated imprecisely. Of 22 453 meta‐analyses from the Cochrane Database of Systematic Reviews (CDSR), containing at least two studies, almost 75% contained five or fewer studies (Davey *et al*, 2011). A Bayesian approach to meta‐analysis is beneficial in allowing an analyst to incorporate information on the likely extent of heterogeneity by drawing on relevant external evidence (Higgins and Whitehead, 1996; Pullenayegum, 2011; Turner *et al*, 2012; Rhodes *et al*, 2015).

This paper presents large‐scale empirical evidence, from published meta‐analyses, on inconsistency across studies in meta‐analyses using binary, continuous and mixed outcome data (including data from time‐to‐event outcomes, ordinal outcomes and binary or continuous data from studies with complex designs). Three binary outcome measures are considered: odds ratio (OR); relative risk (RR) and risk difference (RD). For continuous outcome data, we consider the SMD and mean difference. We also explore the sensitivity of inconsistency to switching the event for the relative risk and opting for a relative measure (the ratio of means) as opposed to a commonly used difference measure for continuous outcome meta‐analysis. To assist Bayesian meta‐analysis, we provide predictive distributions for the degree of inconsistency expected among studies in future meta‐analyses in specific research settings. These distributions can be used in new meta‐analyses to inform prior distributions for the between‐study variance.

This paper has three main sections. Section 2 introduces the data set and describes our methods of statistical analysis. Section 3 presents the findings of this research, focusing on three primary objectives: (i) to compare levels of inconsistency across meta‐analyses using various types of outcome data; (ii) to investigate how inconsistency among studies may differ according to the scale on which the meta‐analysis is performed; and (iii) to illustrate the use of empirical evidence on inconsistency, in order to facilitate Bayesian meta‐analysis with an informative prior for the between‐study variance. We conclude with a discussion in Section 4.

## 2 Methods

### 2.1 Data description

The Cochrane Database of Systematic Reviews (CDSR) is a rich resource of systematic reviews in areas of health care. These reviews have been prepared by the Cochrane Collaboration, with the objective to make the most up to date and reliable evidence conveniently available to healthcare consumers, professionals and providers (Davey *et al*, 2011). For the purpose of this research, data from the CDSR (Issue 1, 2008) were provided by the Nordic Cochrane Centre.

**Figure 1:**
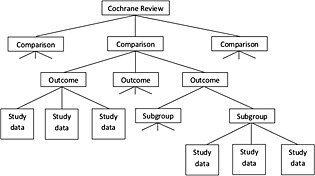
Flow diagram illustrating the structure of the data set.

Cochrane reviews typically include multiple meta‐analyses, which correspond to comparisons of interventions and the assessment of various outcomes within these comparisons. For example, a review examining antibiotics could report separate meta‐analyses comparing each of the several antibiotics against a placebo, with respect to both infection severity and adverse effects. The structure of the data set is illustrated in Figure 1. Meta‐analyses were included in our analyses if they consisted of data from at least two studies. In some reviews, results from studies eligible for a meta‐analysis were available but no pooled results were published in the Cochrane review. Such data were regarded in the same way as meta‐analyses in order to maximise the amount of information available. The review authors may have decided not to perform meta‐analysis based on the degree of heterogeneity between studies (Davey *et al*, 2011).

Reviews could present results for several subgroup analyses within each outcome (Figure 1). Because we are interested in the overall degree of inconsistency in a meta‐analysis, results for study data were combined across subgroups. In some reviews, the subgroups presented within an outcome were not mutually exclusive; therefore, we checked for study duplications and used data for only the first occurrence of each study in each meta‐analysis (Davey *et al*, 2011).

The database contains four types of data: binary data, continuous data, generic results and “O‐E and variance” data. Binary data arise when each participant in a study can have one of two possible outcomes (event or no event), for example, death or survival. We refer to “continuous” data as numerical data that can take any value in a specified range, for example, height and weight. As well as binary or continuous data from studies with complex designs, generic results include ordinal outcomes and time‐to‐event outcomes. Ordinal outcome data occur when participants in a study are classified into ordered categories, for example, disease severity is often classified into categories of “mild”, “moderate” or “severe”. Studies with “O‐E and variance” data tend to represent time‐to‐event outcomes. Time‐to‐event data arise when interest lies in the time to elapse until an event occurs, for example, time from diagnosis or start of treatment to death, time to re‐admission to hospital after discharge or time to healing of a wound.

In the CDSR database, binary data are numbers of events, and continuous data are means and standard deviations, together with the number of participants in each intervention arm in each study. Generic results are an effect estimate, such as a hazard ratio, and corresponding standard error for each study, and “O‐E and variance” data are the observed‐minus‐expected number of events and variance for each study.

All meta‐analyses in the database had been classified according to the type of outcome, types of interventions evaluated and therapeutic area, as described in an earlier paper (Davey *et al*, 2011). The outcomes, intervention comparisons and therapeutic areas were assigned to fairly narrow categories, which we later grouped together. When grouping outcomes for analyses of binary and continuous outcome data, we chose the same categories as for empirical investigations of between‐study heterogeneity using the same data set (Turner *et al*, 2012; Rhodes *et al*, 2015). Based on the distribution of outcomes in the original CDSR data set, it seemed appropriate to group outcomes for analysis of mixed outcome data in the same way as for the binary data (Table 1). For each meta‐analysis in the data set, we are given the scale on which the Cochrane review authors performed meta‐analysis (e.g. RR, RD and SMD). The classifications of outcome types, types of intervention comparison and therapeutic area enabled Turner *et al* (2012) and Rhodes *et al* (2015) to explore how meta‐analysis characteristics influence the extent of between‐study heterogeneity in meta‐analyses of binary and continuous outcome data. Likewise, they allow us to investigate the impact of meta‐analysis characteristics on the degree of inconsistency among studies in a meta‐analysis.

**Table 1:** Frequencies of outcome types, intervention comparison types and therapeutic areas among meta‐analyses (MAs).

	No. of meta‐analyses (%)					
Outcome type	Binary outcome MAs		Continuous outcome MAs		Mixed outcome MAs	
All‐cause mortality	508	(13%)			145	(16%)
Semi‐objective outcomes a	1078	(28%)			122	(14%)
Subjective outcomes b	2287	(59%)			613	(70%)
Obstetric outcomes			165	(3%)		
Resource use and hospital stay/process			417	(8%)		
Internal and external structure‐related outcome			125	(2%)		
General physical health‐related outcomes c			2006	(39%)		
Signs/symptoms reflecting continuation/end of condition and Infection/onset of new acute/chronic disease			845	(16%)		
Mental health outcomes			306	(6%)		
Biological‐markers d			996	(19%)		
Various subjectively measured outcomes e			272	(5%)		
Intervention comparison type						
Pharmacological versus placebo/control	1394	(36%)	2030	(39%)	315	(36%)
Pharmacological versus pharmacological	1014	(26%)	1004	(20%)	203	(23%)
Non‐pharmacological versus any intervention f	1465	(38%)	2098	(41%)	362	(41%)
Therapeutic area						
Cancer	186	(5%)	21	(0.4%)	187	(21%)
Cardiovascular	278	(7%)	354	(7%)	14	(2%)
Central nervous system/ musculoskeletal	337	(9%)	544	(11%)	35	(4%)
Digestive system	429	(11%)	1028	(20%)	70	(8%)
Infectious diseases	273	(7%)	138	(3%)	38	(4%)
Mental health and behavioural conditions	534	(14%)	538	(10%)	36	(4%)
Obstetrics and gynaecology	703	(18%)	626	(12%)	74	(8%)
Pathological conditions	157	(4%)	149	(3%)	21	(2%)
Respiratory diseases	327	(8%)	1050	(20%)	297	(34%)
Urogenital	236	(6%)	337	(7%)	9	(1%)
Other	413	(11%)	347	(7%)	99	(11%)

MAs, meta‐analyses.

a Semi‐objective outcomes include cause‐specific mortality, composite mortality/morbidity, major morbidity event, obstetric outcomes, internal structure, external structure, surgical device success/failure, withdrawals/drop outs, resource use and hospital stay/process measures.

b Subjective outcomes include pain, mental health outcomes, dichotomous biological markers, quality of life/functioning, consumption, satisfaction with care, general physical health, adverse events, infection/new disease, continuation/termination of condition being treated and composite endpoint (including at most one mortality/morbidity endpoint).

c General health‐related outcomes include general physical health, adverse events, pain and quality of life/functioning.

d Biological‐markers include quantifiable biological parameters, typically measured in a laboratory, such as blood components.

e Various subjectively measured outcomes include consumption, satisfaction with care, composite endpoint (including at most one mortality/morbidity endpoint) and surgical device related success/failure.

f Non‐pharmacological interventions include interventions classified as medical devices, surgical, complex, resources and infrastructure, behavioural, psychological, physical, complementary, educational, radiotherapy, vaccines, cellular and gene and screening.

An objective of this paper is to provide predictive distributions for the degree of inconsistency expected among studies in future meta‐analyses. In the sections to follow, the predictive distributions are reported so that they can be conveniently selected for use as priors in future meta‐analyses. This was a difficult task because of the dependency of *I* ^2^ on the precisions of the individual studies (Rücker *et al*, 2008) that are specific to each analysis. We placed meta‐analyses into categories for mean study size that were chosen to approximate 25th and 75th quantiles across all outcome types. We assigned meta‐analyses to a few sample size regions for computational convenience and also to keep prior selection simple for other researchers. We categorised binary and mixed outcome meta‐analyses into the following categories for mean study size: fewer than 50 participants, 50 to 200 participants and more than 200 participants. When analysing continuous outcome data, we combined categories of mean study size: 50 to 200 participants and more than 200 participants. As demonstrated in Table 2, studies tend to have smaller sample sizes when the outcome is continuous.

**Table 2:** Structure of the data set.

	*N*	Min	Median	Max	IQR	95% range
Binary outcome meta‐analyses^g^						
No. of comparisons per review	1967 reviews	1	1	20	1 to 2	1 to 8
No. of studies per meta‐analysis	3873 meta‐analyses	2	3	270	2 to 6	2 to 22
Sample size	21902 studies	2	90	1 242 000	46 to 200	16 to 1827
Continuous outcome meta‐analyses^h^						
No. of comparisons per review	1000 reviews	1	1	12	1 to 2	1 to 5
No. of meta‐analyses per comparison	1605 comparisons	1	2	31	1 to 4	1 to 14
No. of studies^i^ per meta‐analysis	5132 meta‐analyses	2	3	98	2 to 5	2 to 15
Sample size	21612 studies	5	65	18 850	34 to 150	14 to 687
Mixed outcome meta‐analyses^j^						
No. of comparisons per review	193 reviews	1	1	9	1 to 2	1 to 5
No. of meta‐analyses per comparison	318 comparisons	1	2	20	1 to 3	1 to 10
No. of studies^k^ per meta‐analysis	880 meta‐analyses	2	3	133	2 to 7	2 to 23
Sample size	5263 studies	2	88	36 510	20 to 254	2 to 1417

IQR, inter‐quartile range.

g Sixty two binary outcome meta‐analyses were excluded where the outcome type did not fit into any of our pre‐defined categories and was classified as “Other”.

h Thirty continuous outcome meta‐analyses were excluded where the outcome type did not fit into any of our pre‐defined categories and was classified as “Other”.

i Among continuous outcome meta‐analyses, 726 studies were excluded due to missing standard deviations.

j Six mixed outcome meta‐analyses were excluded where the outcome type did not fit into any of our pre‐defined categories and was classified as “Other”.

k Mixed outcome data from 81 studies with generic results were excluded because of missing standard deviations, and 101 studies with “O‐E and variance” data were omitted from our analyses because variances of zero do not represent real data (Davey *et al*, 2011).

### 2.2 Statistical analysis

In this work, we focused on *I* ^2^ to quantify the impact of between‐study heterogeneity in a meta‐analysis. *I* ^2^ describes the proportion of variation among results of included studies in a meta‐analysis that is due to heterogeneity rather than within‐study errors. In our analyses, we make use of the relationship *I* ^2^ = *τ* ^2^/(*τ* ^2^ + *σ* ^2^), where *τ* ^2^ is the between‐study variance and *σ* ^2^ is the “typical” within‐study variance for each meta‐analysis (Higgins and Thompson, 2002).

Initial descriptive analyses were based upon the method of moments estimates of between‐study heterogeneity *τ* ^2^ for each meta‐analysis. *I* ^2^ values computed this way are equivalent to the formulation *I* ^2^ = (*Q* − *df*)/*Q*, where *Q* is the usual heterogeneity test statistic and *df* the number of studies less 1. The initial analyses were conducted to explore what the *I* ^2^ data can tell us about how levels of inconsistency among studies in a meta‐analysis may differ according to the type of outcome data and outcome measure used.

In a more formal statistical analysis, we investigated the distributional form of *I* ^2^ under a fully Bayesian framework, incorporating all sources of parameter uncertainty. Previous research suggests a log‐normal or log‐*t* distribution with 5 degrees of freedom for underlying values of between‐study heterogeneity *τ* ^2^ (Pullenayegum, 2011; Turner *et al*, 2012; Rhodes *et al*, 2015). Therefore, the logit‐normal and logit‐*t*_5_ distributions were natural candidate distributions for *I* ^2^. We also considered an inverse‐gamma distribution for *I* ^2^/(1 − *I* ^2^) in order to imply an inverse‐gamma distribution for *τ* ^2^. Higgins and Whitehead (1996) found the inverse‐gamma distribution to be good fit to values of *τ* ^2^. We visually compared the empirical distribution of the method of moments‐based estimates for *I* ^2^ to *I* ^2^ values obtained under a Bayesian framework. After adjusting for meta‐analysis characteristics as covariates, a comparison of model fit based on deviance information criteria (Spiegelhalter *et al*, 2002; Spiegelhalter *et al*, 2014) led to the choice of the logit‐*t*_5_ model for *I* ^2^, implying a log‐*t* distribution with 5 degrees of freedom for *τ* ^2^. We assessed the fit of the logit‐*t*_5_ model for *I* ^2^ using a quantile–quantile plot of posterior medians for *I* ^2^ versus the fitted logit‐*t*_5_ distribution. To demonstrate our model selection procedure, we provide the results based on binary outcome meta‐analyses of log odds ratios in Supplementary Information Section A.3.

We used hierarchical models to analyse study data from each binary outcome meta‐analysis, whilst investigating the influence of meta‐analysis characteristics on the degree of inconsistency among results of included studies. We use $r_{ij}^{a}$ to denote the number of events in treatment arm *a* (*a* = *C*, *T* for Control and Treatment arm being compared) in study *i* of meta‐analysis *j*, from a total number of $n_{ij}^{a}$ patients, each assumed to have probability $\pi_{ij}^{a}$ of having the event. Within each meta‐analysis *j*, a random‐effects model with binomial within‐study likelihoods was fitted to binary outcome data $r_{ij}^{a}/n_{ij}^{a}$ from each study. We analysed data on each of the log OR, log RR and RD scales. Each of these effect measures for binary outcome data are defined in Supplementary Information Section A.1.1. The Bayesian random‐effects model of Smith *et al* (1995) was used for analyses of log odds ratios. Models for meta‐analysis using the log relative risk and risk difference were developed on the basis of methods proposed by Warn *et al* (2002). We assumed

$$\begin{array}{l} r_{ij}^{C}\;∼\;\text{Binomial}\left(\pi_{ij}^{C},\;n_{ij}^{C}\right)\\ r_{ij}^{T}\;∼\;\text{Binomial}\left(\pi_{ij}^{T},\;n_{ij}^{T}\right) \end{array}$$

On the log OR scale:

$$\begin{array}{l} \text{logit}\left(\pi_{ij}^{C}\right)\;=\;\alpha_{ij}\;-\;\theta_{ij}/2\\ \text{logit}\left(\pi_{ij}^{T}\right)\;=\;\alpha_{ij}\;+\;\theta_{ij}/2\\ \;\theta_{ij}\;∼\;N\left(\mu_{j},\;\tau_{j}^{2}\right)\text{,} \end{array}$$

where the *α*_*ij*_ are the baseline log odds and the underlying treatment effects (log odds ratios) *θ*_*ij*_ have normal random‐effects distributions. In the defined model, *μ*_*j*_ corresponds to the summary intervention effect for meta‐analysis *j*, and $\tau_{j}^{2}$ is the underlying between‐study heterogeneity.

To ensure that the models for the log relative risk and risk difference have appropriate parameter values, we need to constrain *θ*_*ij*_ so that $\pi_{ij}^{T}$ lies in the interval [0, 1]. In the log relative risk model, this is the same as constraining log( $\pi_{ij}^{T}$) to the interval (−∞, 0], achieved by confining *θ*_*ij*_ to be less than $-\text{log}\left(\pi_{ij}^{C}\right)$. Each *θ*_*ij*_ can take any value in the range (−∞, ∞), because it is drawn from a normal distribution with mean *μ*_*j*_ and variance $\tau_{j}^{2}$. Denote by $\theta_{ij}^{L}$ and $\theta_{ij}^{U}$ the lower and upper bounds for *θ*_*ij*_, respectively. In the log relative risk models, we let $\theta_{ij}^{U}$ be the minimum of *θ*_*ij*_ and $-\text{log}\left(\pi_{ij}^{C}\right)$, so that $\theta_{ij}^{U}$ can take any value in the required range $\left(-\infty ,-\text{log}\left(\pi_{ij}^{C}\right)\right)$. In the models for the risk difference, we confine *θ*_*ij*_ to the interval $\left[-\pi_{ij}^{C},1-\pi_{ij}^{C}\right]$ so that $\pi_{ij}^{T}\in \left[0,1\right]$. We let $\theta_{ij}^{L}$ be the maximum of *θ*_*ij*_ and $-\pi_{ij}^{C}$, so that it can take any value in the range $\left[-\pi_{ij}^{C},\infty \right)$. Similarly, we let $\theta_{ij}^{U}$ be the minimum of $\theta_{ij}^{L}$ and $1-\pi_{ij}^{C}$, so that $\theta_{ij}^{U}$ is confined to the required range $\left[-\pi_{ij}^{C},1-\pi_{ij}^{C}\right]$ (see Warn *et al* (2002) for additional details).

The equivalent model on the log RR scale replaces the logits of the treatment group risks $\pi_{ij}^{T}$ and the control group risks $\pi_{ij}^{C}$ by logs so that *θ*_*ij*_ are the log relative risks:

$$\begin{array}{l} \log\left(\pi_{ij}^{C}\right)\;=\;\alpha_{ij}\;-\;\theta_{ij}^{U}/2\\ \log\left(\pi_{ij}^{T}\right)\;=\;\alpha_{ij}\;+\;\theta_{ij}^{U}/2\\ \;\theta_{ij}^{U}\;=\;\min\left(\theta_{ij},\;-\log\left(\pi_{ij}^{C}\right)\right),\\ \;\theta_{ij}\;∼\;N\left(\mu_{j},\;\tau_{j}^{2}\right) \end{array}$$

For the RD scale, we replace the logits in the model on the log odds ratio scale by the risks themselves:

$$\begin{array}{l} \pi_{ij}^{C}\;=\alpha_{ij}\;-\;\theta_{ij}^{U}/2\\ \pi_{ij}^{T}\;=\alpha_{ij}\;+\;\theta_{ij}^{U}/2\\ \theta_{ij}^{U}\;=\;\min\left(\theta_{ij}^{L},\;1\;-\pi_{ij}^{C}\right)\\ \theta_{ij}^{L}\;=\;\max\left(\theta_{ij},\;-\pi_{ij}^{C}\right)\\ \theta_{ij}\;∼\;N\left(\mu_{j},\;\tau_{j}^{2}\right)\text{,} \end{array}$$

where *θ*_*ij*_ are now risk differences.

$I_{j}^{2}$ describes the proportion of variation in study results of meta‐analysis *j* that is due to heterogeneity rather than within‐study errors. We considered a statistic of the form

$$I_{j}^{2}\;=\;\frac{\tau_{j}^{2}}{\tau_{j}^{2}\;+\sigma_{j}^{2}}\text{,}$$

as proposed by Higgins and Thompson (2002).

All Bayesian hierarchical models fitted to the CDSR data set make use of the definition:

$$\log\left(\tau_{j}^{2}\right)\;=\text{logit}\left(I_{j}^{2}\right)\;+\;\log\left(\sigma_{j}^{2}\right)$$

Although this definition explicitly involves a fixed within‐study variance $\sigma_{j}^{2}$, which will vary between studies in practise, we computed a “typical” within‐study variance in meta‐analysis *j* as

$${\hat{\sigma }}_{j}^{2}\;=\frac{\sum_{i}{\hat{\sigma }}_{ij}^{-2}\left(k_{j}-1\right)}{{\left(\sum_{i}{\hat{\sigma }}_{ij}^{-2}\right)}^{2}\;-\sum_{i}{\hat{\sigma }}_{ij}^{-2}}\text{,}$$

where ${\hat{\sigma }}_{ij}^{2}$ are within‐study precisions and *k*_*j*_ is the number of studies included in meta‐analysis *j* ( ${\hat{\sigma }}_{j}^{2}$ is equation 9 in Higgins and Thompson (2002)). Each ${\hat{\sigma }}_{ij}^{2}$ was computed outside WinBUGS using the observed data ( $r_{ij}^{C},n_{ij}^{C},r_{ij}^{T},n_{ij}^{T}$).

Mittlböck and Heinzl (2006) performed a simulation study to examine measures of heterogeneity in a meta‐analysis. They argued that the use of this within‐study variance estimate ${\hat{\sigma }}_{j}^{2}$ is preferable to using the reciprocal of the arithmetic mean weight as it better explains the effect of heterogeneity. Ideally, we would have incorporated uncertainty in the estimated within‐study variances ${\hat{\sigma }}_{ij}^{-2}$, but this proved too computationally intensive, with many parameters in the model being very imprecisely estimated.

In an earlier stage of this research, we fitted a hierarchical regression model to underlying values of log‐transformed between‐study variance $\tau_{j}^{2}$ across binary outcome meta‐analyses, assuming a normal distribution for the residual variation (Turner *et al*, 2012). Here, the hierarchical regression model was fitted to underlying values of logit $\left(I_{j}^{2}\right)$, assuming the preferred *t*‐distribution with 5 degrees of freedom. We crudely adjusted for the important dependency of *I* ^2^ on within‐study precisions, as well as between‐study variation *τ* ^2^, by including a categorical covariate for mean study size in the regression model.

In the defined regression model, *x*_1*j*_ and *x*_2*j*_ are indicators for whether the binary outcome meta‐analysis indexed *j* had an outcome that was all‐cause mortality or semi‐objective, respectively. Likewise, *z*_1*j*_ and *z*_2*j*_ are binary indicators for whether the intervention comparison was pharmacological versus placebo/control or pharmacological versus pharmacological, respectively. Here, *s*_1*j*_ and *s*_2*j*_ are indicators for whether meta‐analysis *j* had a mean study size fewer than 50 participants or more than 200 participants, respectively. After adjustment for other predictors of inconsistency *I* ^2^ in the model, regression coefficients represent average differences in *I* ^2^ on the logit scale among meta‐analyses of different characteristics. *β*_1_ and *β*_2_ are regression coefficients that represent average differences between each outcome type and the reference group of subjective outcomes, whereas the fixed effects *γ*_1_ and *γ*_2_ denote the average differences between each intervention comparison type and the reference group of non‐pharmacological intervention comparisons. Similarly, we included fixed effects *ξ*_1_ and *ξ*_2_ in the regression model to represent average differences between meta‐analyses grouped by mean study size and the reference group of meta‐analyses with mean study size between 50 and 200 participants. Additional fixed effects *δ*_1_, …, *δ*_10_ were added to the regression model to investigate differences between each therapeutic area and the largest group of meta‐analyses related to obstetrics and gynaecology. Error terms *ε*_*uvj*_ allow for residual variation across meta‐analyses, with separate variances for each pairwise combination of outcome types *u* and intervention comparison types *v*.

$$\text{logit}\left(I_{j}^{2}\right)\;=\;\alpha \;+\;\beta_{1}x_{1j}\;+\;\beta_{2}x_{2j}+\;\gamma_{1}z_{1j}\;+\;\gamma_{2}z_{2j}\;+\;\xi_{1}s_{1j}\;+\xi_{2}s_{2j}\;+\;\sum_{p=1}^{10}\delta_{p}a_{pj}+\in_{uvj}\text{,}$$

where $\in_{uvj}\;∼\;t\left(0,\phi_{uv}^{2},5\right)$, for all *u* = 1, 2, 3 and *υ* = 1, 2, 3.

A fully Bayesian meta‐analysis requires prior distributions to be specified for unknown parameters. Vague normal(0,10) prior distributions were assigned to summary effects *μ*_*j*_ and all regression coefficients as recommended by Spiegelhalter et al. (2004), whilst we specified uniform(0,1) prior distributions for underlying probabilities of events $\pi_{ij}^{C}$ in the same way as Warn *et al* (2002). As priors for variance parameters of the random effects, we used inverse‐gamma(0.1,0.1) distributions as in previous work (Rhodes *et al*, 2015).

When analysing data from continuous outcome meta‐analyses, we used similar Bayesian hierarchical models. However, in these cases, we assumed normality of observed study‐level effects because we do not have patient‐level data. Study data were analysed on both the mean difference and SMD scales. Definitions of the mean difference and SMD are provided in Supplementary Information Section A.1.2 together with the mathematical forms of all fitted models. Analyses of mixed outcome data similarly assumed that the summary statistics (e.g. log hazard ratios) had an approximate normal distribution.

All models were fitted using the Markov chain Monte Carlo within the WinBUGS (Version 1.4.3) software (Lunn *et al*, 2000), and results were based on 50 000 iterations following an initial period of 10 000 iterations. This was sufficient to achieve convergence. Convergence diagnostics were run on the 50 000 monitored iterations. We monitored convergence using the Brooks–Gelman–Rubin statistic (Brooks and Gelman, 1998), as implemented in WinBUGS, with three chains starting from widely dispersed initial values. For Markov chain Monte Carlo with a single chain, convergence was checked graphically via trace plots and autocorrelation plots.

For each research setting defined by outcome type, type of intervention comparison, therapeutic area and mean study size, we obtained a predictive distribution for inconsistency $I_{\text{new}}^{2}$ in a new meta‐analysis in that setting, within the full Bayesian model. For example, the predictive distribution for *I* ^2^ in a new binary outcome meta‐analysis assessing a semi‐objective outcome (*x*_2*new*_ = 1), comparing a pharmacological intervention with a placebo (*x*_1*new*_ = 1) in the reference therapeutic area, with a mean study size of less than 50 participants (*s*_1*new*_ = 1), is found by monitoring the following:

$$\log\text{it}\left(I_{\text{new}}^{2}\right)∼\;t\left(\alpha \;+\beta_{2}\;+\;\gamma_{1}\;+\xi_{1},\phi_{21}^{2},5\right)$$

In our initial analyses, we compared the fit of various models that differed according to the meta‐analysis characteristics included as covariates. The inclusion of indicators for therapeutic area led to improvement in model fit based on deviance information criteria. However, the obtained predictive distributions tended to be very similar across therapeutic areas. Where the distributions were very close, we report a set of predictive distributions for *I* ^2^ expected in research settings defined only by outcome type, type of intervention comparison and mean study size. These distributions were obtained by fixing each indicator for therapeutic area equal to the corresponding proportion of meta‐analyses in the data. We consider it undesirable to report more predictive distributions than necessary.

We used WinBUGS to obtain 50 000 samples from the posterior distribution of logit $\left(I_{\text{new}}^{2}\right)$ after convergence. To allow us to summarize the distributions easily, we report *t* distributions with 5 degrees of freedom fitted to each sample of values for logit( $I_{\text{new}}^{2}$), using the *R* function *fitdistr* in library *MASS*. This provided parametric distributions approximating the predictive distributions obtained under the full Bayesian model. These distributions can serve to inform priors for the between‐study variance *τ* ^2^ in new meta‐analyses.

## 3 Results

### 3.1 Descriptive analysis

The data set used for our statistical analyses includes 3873 binary outcome, 5132 continuous outcome and 880 mixed outcome meta‐analyses, including at least two studies. For computational convenience, we analysed data from a subset of binary outcome meta‐analyses in the original CDSR data set. Our data set includes the first reported binary outcome meta‐analysis for each comparison of interventions within each Cochrane review. The original CDSR database includes far fewer continuous outcome meta‐analyses than meta‐analyses of binary outcomes. We therefore decided not to take one continuous outcome meta‐analysis per intervention comparison but, instead, to take all those that were originally published in Cochrane reviews on the mean difference scale. For these meta‐analyses, we could compare the mean difference and standardized mean difference. Of the mixed outcome meta‐analyses, 79% (692 meta‐analyses) combine studies with generic results, whilst the remaining 21% (188 meta‐analyses) combine studies with “O‐E and variance” data. Table 2 shows the structure of the data set.

Table 1 displays the frequencies of outcome types, intervention comparison types and therapeutic areas among the meta‐analyses included in our statistical analyses. Each meta‐analysis compares two types of intervention, which we classified according to three broad categories (pharmacological, placebo or control, and non‐pharmacological). Meta‐analyses comparing pharmacological interventions dominate the data set; 38% compare a pharmacological intervention against a placebo or control, and 22% compare two pharmacological interventions. The frequencies of therapeutic areas are shown in Table 1. Among binary outcome meta‐analyses, obstetrics and gynaecology are the most frequently occurring category (18% of meta‐analyses). Meta‐analyses specializing in respiratory diseases (20% of meta‐analyses) and the digestive system (20% of meta‐analyses) are most frequent among continuous data. Meta‐analyses in respiratory disease are also most frequent among the mixed outcome data (34% of meta‐analyses).

### 3.2 Comparing inconsistency across types of outcome data

A primary aim of this investigation is to compare levels of inconsistency across meta‐analyses using various types of outcome data. Initial descriptive analyses were based upon the method of moments estimates of between‐study heterogeneity *τ* ^2^ for each meta‐analysis, from which we derived estimates for *I* ^2^ values. For each type of outcome data, a histogram representing the empirical distribution of positive estimates for *I* ^2^ on the logit scale is provided in Supplementary Information Section A.2. Provided in Supplementary Information Section A.2.1 are empirical quantile–quantile plots that compare the distributions for the three types of outcome data.

**Figure 2:**
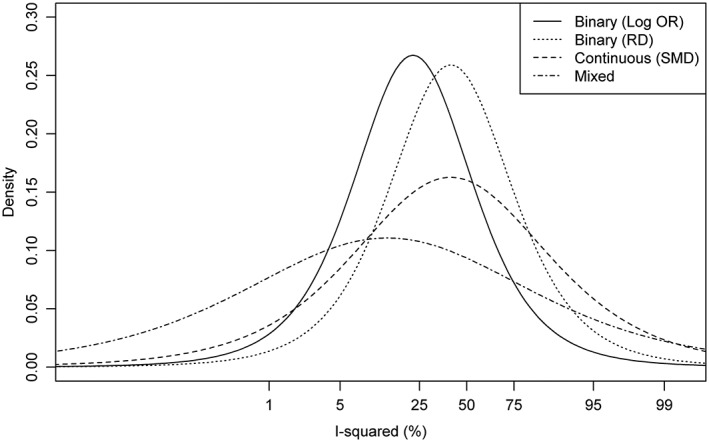
Predictive distributions for logit(*I* ^2^) expected in future meta‐analyses in a general research setting.

Initial analyses based on the method of moments estimation demonstrate that the data may tell us useful information about how inconsistency across studies can differ according to the type of outcome data. As formal statistical analysis, we used Bayesian hierarchical modelling, allowing for all sources of parameter uncertainty. For each type of outcome data and outcome metric, we report a predictive distribution for inconsistency across studies in a future meta‐analysis in a general research setting. These were obtained from hierarchical models fitted to all meta‐analyses in the data set, including no meta‐analysis characteristics as covariates. The estimated fitted distributions for logit $\left(I_{\text{new}}^{2}\right)$ are reported in Table 3, together with summary statistics for $I_{\text{new}}^{2}$ on the untransformed scale. Density plots representing fitted distributions for $I_{\text{new}}^{2}$ on the logit scale are displayed in Figure 2.

**Table 3:** Predictive distributions for logit(*I*^2^) expected in a future meta‐analysis in a general setting.

Outcome data	Outcome metric	Predictive *t*_5_ ldistribution		Median	IQR	95% range	*Pr* (*I* ^2^ < 5 %)
		*μ*	*σ*				
Binary	Log odds ratio	−1.25	1.42	22%	12% to 39%	2% to 82%	0.093
Binary	Log relative risk	−1.35	1.68	21%	9% to 39%	9% to 88%	0.136
Binary	Risk difference	−0.37	1.47	41%	24% to 61%	4% to 92%	0.038
Continuous	Mean difference	−0.16	2.31	46%	18% to 77%	0.8% to 99%	0.096
Continuous	Standardized mean difference	−0.38	2.33	40%	15% to 73%	0.6% to 99%	0.112
Mixed outcome		−1.85	3.43	13%	2% to 53%	0.02% to 99%	0.362

IQR, inter‐quartile range.

l *t*‐distribution with location *μ*, scale *σ* and 5 degrees of freedom.

There are clear differences across the three types of outcome data irrespective of the scale of analysis; the fitted distributions for binary outcome meta‐analyses of log odds ratios and log relative risks have lower medians, 25%, 75% and 97% quantiles, compared with the predictive distributions for inconsistency expected in continuous outcome meta‐analyses (Table 3). The fitted distribution based on analyses of binary outcome data on the risk difference scale has a similar median to those based on analyses of continuous outcome data, but gives support to a narrower range of *I* ^2^ values. The fitted distribution for a mixed outcome meta‐analysis has the lowest median and a considerably higher probability of a very low degree of inconsistency across studies (*I* ^2^ < 5 %).

### 3.3 Assessing the sensitivity of *I*^2^ to the choice of outcome measure

This section seeks to investigate how inconsistency among studies may differ according to the scale on which meta‐analysis is performed.

#### 3.3.1 Binary outcome meta‐analyses

**Figure 3:**
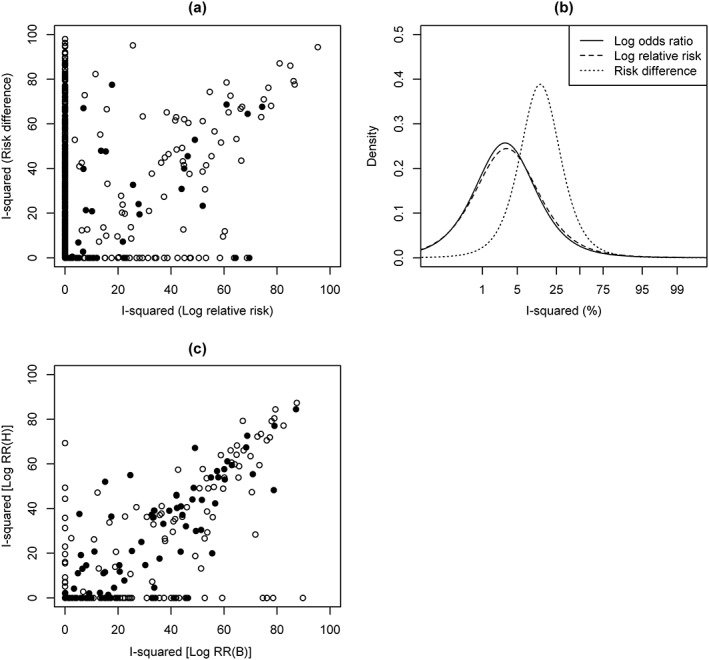
Results to assess the sensitivity of *I* ^2^ to the choice of binary outcome metric. (a) For all 2084 meta‐analyses with *I* ^2^=0 on the log odds ratio scale, a scatter plot for comparison of the risk difference and the log relative risk. In 1566 (75%) of these meta‐analyses *I* ^2^=0 on both the log relative risk and risk difference scales. 375 (18%) of the 2084 meta‐analyses have *I* ^2^>0 on the risk difference scale while *I* ^2^=0 on the log relative risk scale. 55 (3%) of the 2084 meta‐analyses have *I* ^2^>0 on the log relative risk scale while *I* ^2^=0 on the risk difference scale. (b) Predictive distributions for logit(*I* ^2^) expected in pharmacological vs placebo/control meta‐analyses with an all‐cause mortality outcome and mean study size 50 to 200 participants. (c) A scatter plot for comparison of the log relative risk of death (RR(H)) and log relative risk of survival (RR(B)). 59 (12%) meta‐analyses have *I* ^2^>0 on the log(RR(B)) scale while *I* ^2^=0 on the log(RR(H)) scale. 14 (3%) meta‐analyses have *I* ^2^=0 on the log(RR(B)) scale while *I* ^2^>0 on the log(RR(H)) scale. Filled points in scatter plots correspond to meta‐analyses with at least six studies.

We first focus on results from analysing binary outcome data in the CDSR data set. The inconsistency of the study results for each meta‐analysis was initially measured using *I* ^2^ statistics that were each obtained from the method of moment based estimate for between‐study variance *τ* ^2^. In 2084 (54%) binary outcome meta‐analyses performed on the log odds ratio scale, the method of moments estimates for *τ* ^2^ and *I* ^2^ were negative and hence set to zero. Positive estimates for *I* ^2^ have a median of 50% and inter‐quartile range (IQR) 28% to 68%. Results give some indication that *I* ^2^ could be reduced substantially by using an alternative outcome metric. For all 2084 meta‐analyses with *I* ^2^ estimated as zero when based on the log odds ratio scale, a plot of the inconsistency statistics for the comparison of the risk difference with the log relative risk is given in Figure 3(a). Seemingly, re‐analysing the data on the log RR or the RD scale could result in a considerably higher estimate for *I* ^2^.

There is evidence that increases in the average and range of control group event rates that are associated with the increasing degree of inconsistency *I* ^2^ (Table 4). Positive *I* ^2^ estimates for the Log OR and Log RR‐based analyses have similar medians when event rates are low but differ when average events rates are considerably high (> 80 %). *I* ^2^ estimates of zero are more frequent when the average and range of control group event rates are lower. The RD is less consistent than both the OR and RR under all scenarios, and *I* ^2^ estimates of zero are less frequent for the RD analyses.

**Table 4:** Summary of the method of moments‐based estimates of inconsistency *I*^2^ observed in 3873 binary outcome meta‐analyses, comparing log OR, log RR and RD based analyses. *N* denotes the number of meta‐analyses; mean CGER is the unweighted mean of the observed control group event rates in each meta‐analysis; range CGER is the difference between the highest and lowest observed control group event rates.

		Median non‐zero *I* ^2^ (% where *I* ^2^ = 0 %)		
CGER	*N*	Log OR	Log RR	RD
Mean				
0 to 20%	1727	43% (68%)	41% (68%)	52% (56%)
>20% to 40%	959	50% (46%)	49% (48%)	59% (40%)
>40% to 60%	593	53% (40%)	52% (41%)	61% (35%)
>60% to 80%	399	55% (31%)	58% (30%)	66% (27%)
>80%	195	53% (52%)	62% (44%)	62% (39%)
Range				
0 to 20%	1952	47% (68%)	48% (68%)	54% (60%)
>20% to 40%	1003	48% (48%)	51% (48%)	56% (38%)
>40% to 60%	545	51% (33%)	49% (35%)	62% (23%)
>60% to 80%	281	52% (30%)	55% (27%)	66% (17%)
>80%	92	52% (18%)	57% (18%)	71% (7%)

OR, odds ratio; RR, relative risk; RD, risk difference.

In our formal statistical analyses, we investigated the influences of meta‐analysis characteristics on levels of inconsistency among studies in a meta‐analysis, under a fully Bayesian framework. Inferences regarding associations between meta‐analysis characteristics and the impact of heterogeneity were similar for analyses conducted on the log odds ratio, log relative risk or risk difference scale. The section to follow reports predictive distributions for inconsistency expected in various research settings. Table 5 summarizes the predictive distributions for logit(*I* ^2^) expected in meta‐analyses of log odds ratios. The predictive distributions for inconsistency expected in meta‐analyses of log relative risks and risk differences can be found in Supplementary Information Section A.4. The fitted distributions for logit( $I_{\text{new}}^{2}$) based on analyses of log odds ratios and log relative risks are similar, but the distributions based on analyses of risk differences give greater support to higher levels of inconsistency. The density plots in Figure 3(b), representing the predictive distributions for logit(*I* ^2^), illustrate these findings. These densities correspond to the predictive distributions for pharmacological versus placebo/control meta‐analyses, with an all‐cause mortality outcome and a mean study size between 50 and 200 participants.

**Table 5:** Binary outcome data: predictive distributions for logit(*I*^2^) expected in future meta‐analyses of log odds ratios, together with summary statistics for *I*^2^ on the untransformed scale. A *t*(*μ*, *σ*^2^, 5) distribution represents a t‐distribution with location *μ*, scale *σ* and 5 degrees of freedom. *N* denotes the total number of meta‐analyses contributing in each category.

	Pharmacological versus placebo/control	Pharmacological versus pharmacological	Non‐pharmacological (any)
Mean study size < 50 participants			
All‐cause mortality	*t*(−4.02, 1.48^2^, 5)	*t*(−4.44, 0.66^2^, 5)	*t*(−3.78, 1.713^2^, 5)
	Median = 2%	Median = 1%	Median = 2%
	IQR = 1% to 4%	IQR = 1% to 2%	IQR = 1% to 5%
	95% range = 0.09% to 23%	95% range = 0.3% to 4%	95% range = 0.06% to 38%
	*Pr*(*I* ^2^ < 5 %) = 0.810	*Pr*(*I* ^2^ < 5 %) = 0.982	*Pr*(*I* ^2^ < 5 %) = 0.729
	*N* = 39	*N* = 17	*N* = 29
Semi‐objective	*t*(−2.25, 1.27^2^, 5)	*t*(−2.67, 1.35^2^, 5)	*t*(−2.06, 1.81^2^, 5)
	Median = 10%	Median = 6%	Median = 12%
	IQR = 5% to 18%	IQR = 3% to 13%	IQR = 5% to 25%
	95% range = 0.7% to 55%	95% range = 0.4% to 50%	95% range = 0.3% to 82%
	*Pr*(*I* ^2^ < 5 %) = 0.234	*Pr*(*I* ^2^ < 5 %) = 0.397	*Pr*(*I* ^2^ < 5 %) = 0.272
	*N* = 70	*N* = 29	*N* = 90
Subjective	*t*(−1.34, 1.11^2^, 5)	*t*(−1.77, 1.20^2^, 5)	*t*(−1.14, 0.82^2^, 5)
	Median = 21%	Median = 15%	Median = 24%
	IQR = 12% to 33%	IQR = 8% to 25%	IQR = 2% to 34%
	95% range = 3% to 70%	95% range = 2% to 63%	95% range = 6% to 64%
	*Pr*(*I* ^2^ < 5 %) = 0.061	*Pr*(*I* ^2^ < 5 %) = 0.123	*Pr*(*I* ^2^ < 5 %) = 0.016
	*N* = 154	*N* = 123	*N* = 140
Mean study size between 50 and 200 participants			
All‐cause mortality	*t*(−3.52, 1.48^2^, 5)	*t*(−3.94, 0.66^2^, 5)	*t*(−3.28, 1.71^2^, 5)
	Median = 3%	Median = 2%	Median = 4%
	IQR = 1% to 6%	IQR = 1% to 3%	IQR = 1% to 3%
	95% range = 0.1% to 34%	95% range = 0.6% to 7%	95% range = 0.09% to 51%
	*Pr*(*I* ^2^ < 5 %) = 0.677	*Pr*(*I* ^2^ < 5 %) = 0.951	*Pr*(*I* ^2^ < 5 %) = 0.585
	*N* = 99	*N* = 55	*N* = 89
Semi‐objective	*t*(−1.75, 1.26^2^, 5)	*t*(−2.18, 1.35^2^, 5)	*t*(−1.57, 1.80^2^, 5)
	Median = 15%	Median = 10%	Median = 18%
	IQR = 8% to 26%	IQR = 5% to 19%	IQR = 7% to 36%
	95% range = 1% to 68%	95% range = 0.7% to 63%	95% range = 0.5% to 88%
	*Pr*(*I* ^2^ < 5 %) = 0.132	*Pr*(*I* ^2^ < 5 %) = 0.235	*Pr*(*I* ^2^ < 5 %) = 0.184
	*N* = 156	*N* = 152	*N* = 269
Subjective	*t*(−0.84, 1.10^2^, 5)	*t*(−1.28, 1.20^2^, 5)	*t*(−0.65, 0.81^2^, 5)
	Median = 30%	Median = 22%	Median = 34%
	IQR = 19% to 45%	IQR = 13% to 35%	IQR = 25% to 45%
	95% range = 4% to 79%	95% range = 3% to 74%	95% range = 9% to 74%
	*Pr*(*I* ^2^ < 5 %) = 0.030	*Pr*(*I* ^2^ < 5 %) = 0.062	*Pr*(*I* ^2^ < 5 %) = 0.007
	*N* = 480	*N* = 354	*N* = 437
Mean study size > 200 participants			
All‐cause mortality	*t*(−3.17, 1.48^2^, 5)	*t*(−3.59, 0.66^2^, 5)	*t*(−2.93, 1.71^2^, 5)
	Median = 4%	Median = 3%	Median = 5%
	IQR = 2% to 9%	IQR = 2% to 4%	IQR = 2% to 12%
	95% range = 0.2% to 42%	95% range = 0.8% to 9%	95% range = 0.1% to 60%
	*Pr*(*I* ^2^ < 5 %) = 0.569	*Pr*(*I* ^2^ < 5 %) = 0.871	*Pr*(*I* ^2^ < 5 %) = 0.486
	*N* = 78	*N* = 37	*N* = 65
Semi‐objective	*t*(−1.40, 1.27^2^, 5)	*t*(−1.83, 1.35^2^, 5)	*t*(−1.22, 1.81^2^, 5)
	Median = 20%	Median = 14%	Median = 23%
	IQR = 11% to 33%	IQR = 7% to 25%	IQR = 10% to 44%
	95% range = 2% to 76%	95% range = 1% to 70%	95% range = 0.8% to 91%
	*Pr*(*I* ^2^ < 5 %) = 0.089	*Pr*(*I* ^2^ < 5 %) = 0.164	*Pr*(*I* ^2^ < 5 %) = 0.135
	*N* = 81	*N* = 102	*N* = 129
Subjective	*t*(−0.49, 1.11^2^, 5)	*t*(−0.93, 1.20^2^, 5)	*t*(−0.30, 0.82^2^, 5)
	Median = 38%	Median = 29%	Median = 43%
	IQR = 25% to 53%	IQR = 17% to 43%	IQR = 32% to 54%
	95% range = 6% to 84%	95% range = 4% to 80%	95% range = 13% to 80%
	*Pr*(*I* ^2^ < 5 %) = 0.017	*Pr*(*I* ^2^ < 5 %) = 0.036	*Pr*(*I* ^2^ < 5 %) = 0.003
	*N* = 237	*N* = 145	*N* = 217

IQR, inter‐quartile range.

**Figure 4:**
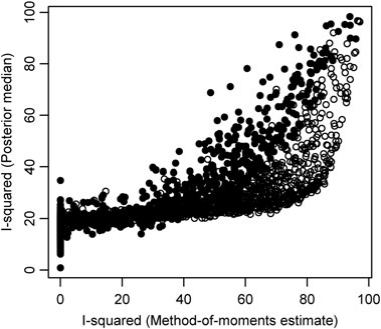
For binary outcome meta‐analyses of log odds ratios, a scatter plot for comparison of method‐of‐moments estimators for *I* ^2^ and Bayesian estimates for *I* ^2^. Bayesian estimates were obtained from a hierarchical model without covariates for meta‐analysis characteristics. Filled points in scatter plots correspond to meta‐analyses with at least six studies.

In our initial descriptive analyses, we used *I* ^2^ values based on the method of moments estimates for *τ* ^2^ and the definition *τ* ^2^/(*τ* ^2^ + *σ* ^2^). Our formal statistical analyses were conducted under a fully Bayesian framework; therefore, it would be useful to explore the relationship between our initial descriptive estimates for *I* ^2^ and Bayesian estimates. For each of the 3873 meta‐analyses performed on the log odds ratio scale, we compare the method of moments estimators for *I* ^2^ and posterior medians for *I* ^2^ obtained from the Bayesian hierarchical model fitted without covariates for meta‐analysis characteristics (Figure 4). For meta‐analyses with more than five studies, the differences between the two estimates for *I* ^2^ range from 0.07% to 35% with median 14% (IQR: 9% to 17%). For meta‐analyses with at most five studies, the differences between the two estimates for *I* ^2^ range from 0.08% to 52% with median 19% (IQR:18% to 21%).

The method of moments‐based estimator for *I* ^2^ is negative and hence set to zero in 2084 (54%) meta‐analyses, for which posterior medians of *I* ^2^ range from 1% to 35% with median 19%. When the method of moments‐based estimate for *I* ^2^ is considerably high, Bayesian estimation tends to yield a reduced estimate for *I* ^2^. When the method of moments‐based estimate for *I* ^2^ is low, Bayesian estimation typically leads to an increased estimate for *I* ^2^. In 1278 (71%) of the 1789 meta‐analyses with a positive method of moments‐based estimate for *I* ^2^, the posterior median of *I* ^2^ is less than the initial descriptive estimator. The positive method of moments estimators that are greater than the posterior medians of *I* ^2^ range from 18% to 97% with median 56% (IQR: 40% to 71%). The positive method of moments estimators that are less than the posterior medians of *I* ^2^ range from 0.06% to 96% with a lower median of 13% (IQR: 6% to 18%). We note that the observed discrepancies between the method of moments based estimates and Bayesian estimates for *I* ^2^ could be attributable to considerable bias in the method of moments estimator of *τ* ^2^ that uses estimates of study‐specific variances (Böhning *et al*, 2002; Hamza *et al*, 2008). This bias may cause the method of moments‐based estimators of *I* ^2^ to be lower than they should be.

#### 3.3.2 Exploring the impact of switching the event definition for relative risks

Many healthcare interventions are designed to either reduce the risk of an adverse outcome or increase the chance of a desirable outcome. In the context of binary outcomes, it is natural to refer to one of the outcome states as being an event (Deeks, 2002). It is possible to switch events and non‐events and focus on the proportion of participants not having the event. Here, we explore the impact of switching the event definition on levels of inconsistency among relative risks.

We decided to focus on a subset of the CDSR data set in which beneficial and harmful events were easily distinguished. A total of 508 binary outcome meta‐analyses examined all‐cause mortality. These meta‐analyses were classified according to whether they measured survival or death as the outcome. Each meta‐analysis was analysed twice on the log relative risk scale with the event and non‐event switched. We denote the relative risk of death as RR(H) and the relative risk of survival as RR(B). Initial descriptive analyses used the method of moments‐based estimates for between‐study variance *τ* ^2^. A plot of *I* ^2^ estimates for the comparison of log RR(H) and the log RR(B) is given in Figure 3(c). The *I* ^2^ estimate is zero for 315 (62%) meta‐analyses, irrespective of the definition of the event for the relative risk. Of the remaining 193 meta‐analyses, 140 (73%) are more consistent when death is treated as the event.

In meta‐analyses where *I* ^2^ on the log RR(H) scale and *I* ^2^ on the log RR(B) scale are equivalent, the average control group event rates have median 0.08 (IQR 0.04 to 0.17). The differences between the highest and lowest observed control group event rates have median 0.11 (IQR 0.04 to 0.22). In the remaining 193 meta‐analyses, differences between *I* ^2^ on the log RR(H) and the log RR(B) scales range from 0.06% to 90%, with median 11% (95% range 0.3% to 61%). The meta‐analyses where *I* ^2^ on the log RR(H) scale and *I* ^2^ on the log RR(B) scale differ by more than 61% have average control group event rates with median 0.18 (IQR 0.11 to 0.29). The differences between the highest and lowest observed control group event rates have median 0.28 (IQR 0.21 to 0.32).

#### 3.3.3 Continuous outcome meta‐analyses

**Figure 5:**
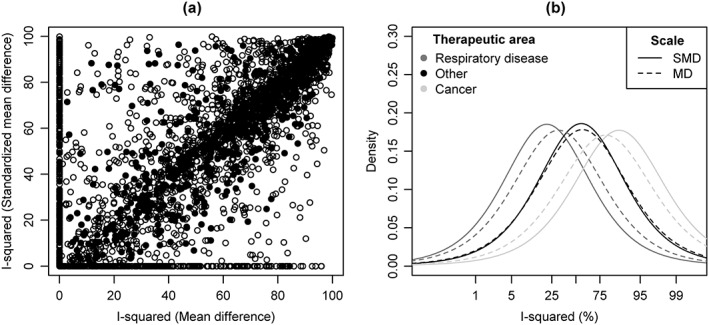
Results to assess the sensitivity of *I* ^2^ to the choice of continuous outcome metric. (a) A scatter plot of *I* ^2^ statistics for comparison of the SMD and the mean difference (MD) scales. Filled points correspond to meta‐analyses with at least six studies. In 1934 (38%) meta‐analyses *I* ^2^=0 on both scales. 311 (6%) meta‐analyses have *I* ^2^>0 on the SMD scale where *I* ^2^=0 on the mean difference scale. 296 (6%) meta‐analyses have *I* ^2^>0 on the mean difference scale where *I* ^2^=0 on the SMD scale. (b) Example predictive distributions for logit(*I* ^2^) expected in future non‐pharmacological meta‐analyses of general health‐related outcomes with a mean study size greater than or equal to 50 participants.

An empirical investigation was conducted to assess inconsistency among SMDs and mean differences across the CDSR data set. In our initial descriptive analyses, the inconsistency of the study results for each of the 5132 meta‐analyses was quantified using *I* ^2^ statistics, derived from method of moments‐based estimates for *τ* ^2^. We obtained negative estimates for *I* ^2^ in 2230 (43%) meta‐analyses on the SMD scale and in 2245 (44%) meta‐analyses on the mean difference scale. Positive estimates for *I* ^2^ based on analyses of SMDs have a median of 66% and IQR 40% to 84%. The positive estimates for *I* ^2^ in meta‐analyses combining mean differences have a similar median of 66% and IQR 39% to 83%. A plot of *I* ^2^ statistics for comparison of the SMD with the mean difference scale is given in Figure 5(a). For some meta‐analyses, it appears that *I* ^2^ could be reduced considerably by performing meta‐analysis on the alternative scale.

In later analyses, we used Bayesian hierarchical models to analyse continuous outcome data from each meta‐analysis on both the SMD and mean difference scales, whilst exploring the influences of meta‐analysis characteristics on inconsistency among studies. Results reflecting comparisons of inconsistency across meta‐analyses of different characteristics were not very sensitive to the scale on which study data are analysed. We obtained sets of predictive distributions for inconsistency expected among studies in future continuous outcome meta‐analyses in different research settings. There is empirical evidence to suggest that heterogeneity is substantially lower in meta‐analyses related to respiratory diseases and considerably higher in meta‐analyses related to cancer than in other therapeutic areas (Rhodes *et al*, 2015). For these reasons, we report separate predictive distributions for *I* ^2^ expected in future meta‐analyses related to respiratory diseases and cancer. For the largest group meta‐analyses related to therapeutic areas other than cancer and respiratory, the predictive distributions based on the SMD scale are summarized in Table 6. All fitted distributions based on the mean difference scale are available in Supplementary Information Section A.4, together with the distributions for SMD‐based analyses related to cancer and respiratory diseases. For comparison of inconsistency among SMDs and mean differences, we contrast the fitted distributions for $I_{\text{new}}^{2}$. With the exception of the very small group of meta‐analyses for cancer, it appears that inconsistency across SMDs tends to be marginally lower, or very similar, to inconsistency across mean differences. The overall strong similarities between the predictive distributions for logit(*I* ^2^) based on the SMD and mean difference scales are illustrated in the example density plots displayed in Figure 5(b). These density plots represent the fitted distributions for logit( $I_{\text{new}}^{2}$) in non‐pharmacological meta‐analyses with general health‐related outcomes and mean study size greater than or equal to 50 participants.

**Table 6:** Continuous outcome data: predictive *t*(*μ*, *σ*^2^, 5) distributions for logit(*I*^2^) expected in future meta‐analyses of standardized mean differences. Summary statistics are for *I*^2^ on the untransformed scale. A *t*(*μ*, *σ*^2^, 5) distribution represents a *t*‐distribution with location *μ*, scale *σ* and 5 degrees of freedom. *N* denotes the total number of meta‐analyses contributing in each category.

	Mean study size < 50 participants			Mean study size ≥ 50 participants		
	Pharmacological versus placebo/control	Pharmacological versus pharmacological	Non‐pharmacological (Any)	Pharmacological versus placebo/ control	Pharmacological versus pharmacological	Non‐pharmacological (any)
Obstetric outcome	*t*(−1.30, 1.91^2^, 5)	*t*(−1.49, 1.84^2^, 5)	*t*(−1.16, 1.88^2^, 5)	*t*(−0.32, 1.90^2^, 5)	*t*(−0.51, 1.83^2^, 5)	*t*(−0.19, 1.87^2^, 5)
	Median = 22%	Median = 19%	Median = 24%	Median = 43%	Median = 38%	Median = 45%
	IQR = 8% to 46%	IQR = 7% to 40%	IQR = 9% to 48%	IQR = 18% to 69%	IQR = 17% to 64%	IQR = 22% to 71%
	95% range = 0.6% to 92%	95% range = 0.5% to 91%	95% range = 0.7% to 93%	95% range = 2% to 97%	95% range = 1% to 96%	95% range = 2% to 97%
	*Pr*(*I* ^2^ < 5 %) = 0.173	*Pr*(*I* ^2^ < 5 %) = 0.181	*Pr*(*I* ^2^ < 5 %) = 0.148	*Pr*(*I* ^2^ < 5 %) = 0.075	*Pr*(*I* ^2^ < 5 %) = 0.081	*Pr*(*I* ^2^ < 5 %) = 0.062
	*N* = 3	*N* = 7	*N* = 8	*N* = 47	*N* = 39	*N* = 61
Resource use and hospital stay/process	*t*(−0.24, 2.24^2^, 5)	*t*(−0.43, 2.18^2^, 5)	*t*(−0.11, 2.27^2^, 5)	*t*( 0.73, 2.24^2^, 5)	*t*( 0.54, 2.18^2^, 5)	*t*( 0.87, 2.27^2^, 5)
	Median = 44%	Median = 40%	Median = 46%	Median = 67%	Median = 63%	Median = 70%
	IQR = 17% to 75%	IQR = 15% to 70%	IQR = 19% to 77%	IQR = 35% to 89%	IQR = 32% to 86%	IQR = 38% to 90%
	95% range = 0.9% to 99%	95% range = 0.8% to 98%	95% range = 1% to 99%	95% range = 2% to 99%	95% range = 2% to 99%	95% range = 3% to 99.5%
	*Pr*(*I* ^2^ < 5 %) = 0.098	*Pr*(*I* ^2^ < 5 %) = 0.107	*Pr*(*I* ^2^ < 5 %) = 0.089	*Pr*(*I* ^2^ < 5 %) = 0.050	*Pr*(*I* ^2^ < 5 %) = 0.051	*Pr*(*I* ^2^ < 5 %) = 0.043
	*N* = 33	*N* = 0	*N* = 16	*N* = 45	*N* = 46	*N* = 200
Internal and external structure‐related outcome	*t*(−0.16, 2.20^2^, 5)	*t*(−0.35, 2.18^2^, 5)	*t*(−0.02, 2.24^2^, 5)	*t*( 0.82, 2.19^2^, 5)	*t*( 0.63, 2.18^2^, 5)	*t*( 0.95, 2.23^2^, 5)
	Median = 46%	Median = 42%	Median = 49%	Median = 69%	Median = 66%	Median = 72%
	IQR = 19% to 77%	IQR = 17% to 72%	IQR = 20% to 79%	IQR = 38% to 98%	IQR = 35% to 87%	IQR = 405 to 91%
	95% range = 0.9% to 99%	95% range = 0.9% to 98%	95% range = 1% to 99%	95% range = 2% to 99%	95% range = 2% to 99%	95% range = 3% to 99.6%
	*Pr*(*I* ^2^ < 5 %) = 0.091	*Pr*(*I* ^2^ < 5 %) = 0.104	*Pr*(*I* ^2^ < 5 %) = 0.083	*Pr*(*I* ^2^ < 5 %) = 0.042	*Pr*(*I* ^2^ < 5 %) = 0.050	*Pr*(*I* ^2^ < 5 %) = 0.041
	*N* = 32	*N* = 3	*N* = 9	*N* = 42	*N* = 1	*N* = 35
General physical health and Adverse event and Pain and Quality of life/functioning	*t*(−0.86, 2.01^2^, 5)	*t*(−1.05, 1.99^2^, 5)	*t*(−0.73, 2.04^2^, 5)	*t*( 0.11, 2.00^2^, 5)	*t*(−0.08, 1.98^2^, 5)	*t*( 0.25, 2.04^2^, 5)
	Median = 30%	Median = 265	Median = 32%	Median = 53%	Median = 48%	Median = 56%
	IQR = 11% to 59%	IQR = 10% to 53%	IQR = 13% to 62%	IQR = 24% to 79%	IQR = 22% to 75%	IQR = 28% to 81%
	95% range = 0.7% to 96%	95% range = 0.7% to 95%	95% range = 0.8% to 97%	95% range = 2% to 98%	95% range = 2% to 98%	95% range = 2% to 99%
	*Pr*(*I* ^2^ < 5 %) = 0.133	*Pr*(*I* ^2^ < 5 %) = 0.145	*Pr*(*I* ^2^ < 5 %) = 0.120	*Pr*(*I* ^2^ < 5 %) = 0.059	*Pr*(*I* ^2^ < 5 %) = 0.066	*Pr*(*I* ^2^ < 5 %) = 0.052
	*N* = 143	*N* = 57	*N* = 249	*N* = 413	*N* = 117	*N* = 388
Signs/symptoms reflecting continuation/end of condition and infection/onset of new acute/chronic disease	*t*(−0.78, 2.11^2^, 5)	*t*(−0.98, 2.10^2^, 5)	*t*(−0.65, 2.12^2^, 5)	*t*( 0.19, 2.10^2^, 5)	*t*( 0.00, 2.10^2^, 5)	*t*( 0.33, 2.12^2^, 5)
	Median = 31%	Median = 28%	Median = 34%	Median = 55%	Median = 50%	Median = 57%
	IQR = 11% to 62%	IQR = 10% to 57%	IQR = 13% to 64%	IQR = 25% to 81%	IQR = 23% to 78%	IQR = 29% to 83%
	95% range = 0.6% to 97%	95% range = 0.5% to 96%	95% range = 0.7% to 98%	95% range = 2% to 99%	95% range = 1% to 99%	95% range = 2% to 99%
	*Pr*(*I* ^2^ < 5 %) = 0.135	*Pr*(*I* ^2^ < 5 %) = 0.147	*Pr*(*I* ^2^ < 5 %) = 0.118	*Pr*(*I* ^2^ < 5 %) = 0.058	*Pr*(*I* ^2^ < 5 %) = 0.071	*Pr*(*I* ^2^ < 5 %) = 0.054
	*N* = 63	*N* = 41	*N* = 76	*N* = 221	*N* = 69	*N* = 143
Mental health outcome	*t*(−0.54, 1.75^2^, 5)	*t*(−0.73, 1.70^2^, 5)	*t*(−0.40, 1.75^2^, 5)	*t*( 0.44, 1.75^2^, 5)	*t*( 0.25, 1.70^2^, 5)	*t*( 0.57, 1.75^2^, 5)
	Median = 30%	Median = 37%	Median = 32%	Median = 61%	Median = 56%	Median = 64%
	IQR = 18% to 62%	IQR = 16% to 57%	IQR = 20% to 65%	IQR = 37% to 81%	IQR = 33% to 77%	IQR = 40% to 83%
	95% range = 2% to 95%	95% range = 2% to 93%	95% range = 2% to 96%	95% range = 4% to 98%	95% range = 4% to 97%	95% range = 5% to 98%
	*Pr*(*I* ^2^ < 5 %) = 0.075	*Pr*(*I* ^2^ < 5 %) = 0.075	*Pr*(*I* ^2^ < 5 %) = 0.063	*Pr*(*I* ^2^ < 5 %) = 0.029	*Pr*(*I* ^2^ < 5 %) = 0.032	*Pr*(*I* ^2^ < 5 %) = 0.025
	*N* = 39	*N* = 16	*N* = 30	*N* = 106	*N* = 46	*N* = 64
Biological‐marker	*t*(−1.23, 2.48^2^, 5)	*t*(−1.43, 2.42^2^, 5)	*t*(−1.10, 2.48^2^, 5)	*t*(−0.26, 2.48^2^, 5)	*t*(−0.45, 2.41^2^, 5)	*t*(−0.12, 2.48^2^, 5)
	Median = 23%	Median = 19%	Median = 25%	Median = 44%	Median = 39%	Median = 47%
	IQR = 6% to 56%	IQR = 6% to 49%	IQR = 7% to 59%	IQR = 14% to 77%	IQR = 14% to 72%	IQR = 16% to 79%
	95% range = 0.2% to 98%	95% range = 0.2% to 97%	95% range = 0.2% to 98%	95% range = 0.5% to 99%	95% range = 0.55 to 99%	95% range = 0.6% to 99%
	*Pr*(*I* ^2^ < 5 %) = 0.221	*Pr*(*I* ^2^ < 5 %) = 0.234	*Pr*(*I* ^2^ < 5 %) = 0.206	*Pr*(*I* ^2^ < 5 %) = 0.120	*Pr*(*I* ^2^ < 5 %) = 0.124	*Pr*(*I* ^2^ < 5 %) = 0.108
	*N* = 175	*N* = 51	*N* = 128	*N* = 212	*N* = 99	*N* = 268
Various subjectively measured outcomes	*t*(−0.31, 2.16^2^, 5)	*t*(−0.50, 2.09^2^, 5)	*t*(−0.18, 2.16^2^, 5)	*t*( 0.66, 2.16^2^, 5)	*t*( 0.47, 2.09^2^, 5)	*t*( 0.80, 2.15^2^, 5)
	Median = 43%	Median = 39%	Median = 46%	Median = 66%	Median = 62%	Median = 69%
	IQR = 17% to 72%	IQR = 15% to 68%	IQR = 19% to 75%	IQR = 35% to 87%	IQR = 22% to 85%	IQR = 39% to 89%
	95% range = 1% to 98%	95% range = 0.9% to 97%	95% range = 1% to 98%	95% range = 3% to 99%	95% range = 2% to 99%	95% range = 2 Pr(I ^2^ < 5 %) = 0.092
	*Pr*(*I* ^2^ < 5 %) = 0.092	*Pr*(*I* ^2^ < 5 %) = 0.099	*Pr*(*I* ^2^ < 5 %) = 0.084	*Pr*(*I* ^2^ < 5 %) = 0.041	*Pr*(*I* ^2^ < 5 %) = 0.046	*Pr*(*I* ^2^ < 5 %) = 0.040
	*N* = 14	*N* = 4	*N* = 31	*N* = 37	*N* = 30	*N* = 104

IQR,inter‐quartile range.

#### 3.3.4 Investigating the use of a relative measure for continuous outcome meta‐analysis

Results suggest that SMDs tend to be slightly more consistent than mean differences. However, levels of inconsistency among difference measures for continuous data are very similar. To explore whether relative measures exhibit lower levels of inconsistency than difference measures for continuous outcome meta‐analysis, we analyse study data from all 165 meta‐analyses assessing an obstetric outcome. These meta‐analyses were chosen because the RoM can be calculated for all included studies in the data set. The RoM is restricted to use in studies where the means on the two treatment arms are either both positive or both negative. This is because we compute the RoM on the natural logarithm scale for mathematical convenience (Friedrich et al., 2008).

**Figure 6:**
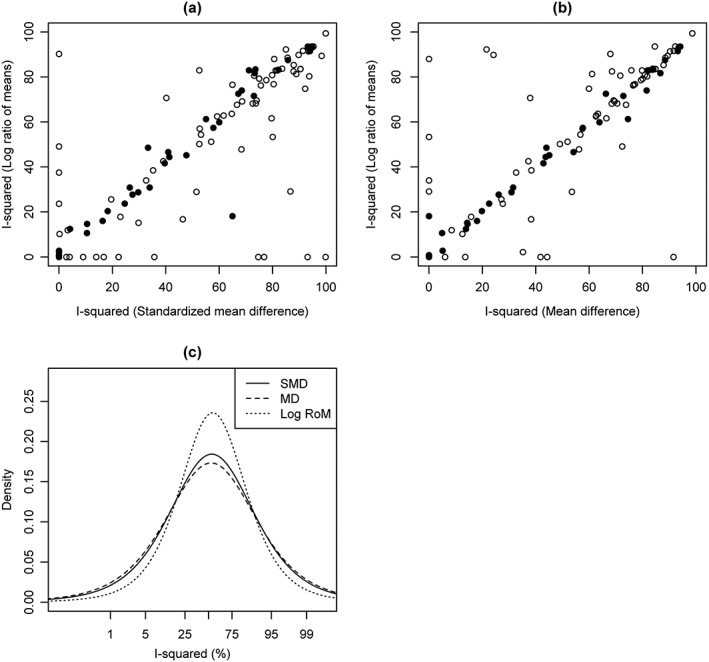
Results for comparison of the log RoM with the SMD and mean difference (MD). Scatter plots for comparison of (a) the log RoM and the SMD and (b) the log RoM and the mean difference. Filled points in scatter plots correspond to meta‐analyses with at least six studies. (c) Predictive distributions for logit(*I* ^2^) expected in future meta‐analyses of obstetric outcomes.

For descriptive purposes, we computed estimates for *I* ^2^ for each continuous outcome meta‐analysis assessing an obstetric outcome using the method of moments estimates for the between‐study variance *τ* ^2^. Plots of inconsistency statistics for comparisons of the ratio of means with the SMD and of the ratio of means with the mean difference are given in Figures 6(a) and (b), respectively. Discrepancies between *I* ^2^ statistics are most apparent for small meta‐analyses including at most five studies.

We fitted hierarchical models that performed Bayesian random‐effects meta‐analysis for each meta‐analysis with an obstetric outcome on each of the mean difference, SMD and log(RoM) scales. Each of these metrics are defined in Supplementary Information Section A.1.2. Predictive *t*_5_ distributions for logit(*I* ^2^) expected in future meta‐analyses are displayed graphically in Figure 6(c) and summarized numerically in Table 7. The fitted distributions based on the SMD and mean difference scales resemble each other strongly, and the distribution based on the log RoM scale is also very similar. This distribution does however seem to give support to a slightly narrower range of *I* ^2^ values. We find that the estimated predictive distributions for *I* ^2^ expected in future meta‐analyses assessing an obstetric outcome have similar quantiles, regardless of the scale on which the meta‐analysis is performed.

**Table 7:** Predictive distributions for logit(*I*^2^) expected in a future continuous meta‐analysis assessing an obstetric outcome.

Outcome metric	Predictive *t*_5_ m distribution		Median	IQR	95% range	*Pr* (*I* ^2^ < 5 %)
	*μ*	*σ*				
Log ratio of Means	0.199	1.610	55%	33% to 75%	4% to 97%	0.031
Mean difference	0.123	2.190	53%	25% to 80%	1% to 99%	0.068
Standardized mean difference	0.155	2.060	54%	26% to 79%	2% to 99%	0.055

m *t*‐distribution with location *μ*, scale *σ* and 5 degrees of freedom.

### 3.4 Incorporating empirical evidence on inconsistency in meta‐analysis

The purpose of this section is to facilitate the incorporation of external evidence on inconsistency among studies in a meta‐analysis. We summarize predictive distributions for inconsistency expected across studies in future meta‐analyses, across research settings, together with summary statistics for *I* ^2^ on the untransformed scale. These distributions can be used to inform priors for the between‐study variance *τ* ^2^ in future meta‐analyses. In an earlier stage of this research project, we conducted empirical investigations of between‐study variance *τ* ^2^. Analyses of binary outcome data found no evidence of differences in between‐study heterogeneity between therapeutic areas (Turner *et al*, 2012). However, analyses of continuous outcome data showed that heterogeneity was substantially lower in meta‐analyses related to respiratory diseases and considerably higher in meta‐analyses related to cancer (Rhodes *et al*, 2015). There was no evidence of a difference between the remaining therapeutic areas not related to respiratory disease or cancer. Exploratory data analysis revealed low frequencies of mixed outcome meta‐analyses for many therapeutic areas. For these reasons, meta‐analysis settings for binary and mixed outcome data were described according to outcome type, intervention comparison type and mean study size. For continuous outcome data, meta‐analysis setting was additionally described according to the therapeutic area.

For each meta‐analysis setting, a predictive *t*_5_ distribution for logit(*I* ^2^) expected in binary outcome meta‐analyses using the log odds ratio is given in Table 5. The predictive distributions for inconsistency expected among log relative risks and risk differences are available in Supplementary Information Section A.4. We report predictive distributions for logit(*I* ^2^) expected in mixed outcome meta‐analyses in Table 8.

**Table 8:** Mixed outcome data: predictive distributions for logit(*I*^2^) expected in future meta‐analyses, together with summary statistics for *I*^2^ on the untransformed scale. A *t*(*μ*, *σ*^2^, 5) distribution represents a *t*‐distribution with location *μ*, scale *σ* and 5 degrees of freedom. *N* denotes the total number of meta‐analyses contributing in each category.

	Pharmacological versus placebo/control	Pharmacological versus pharmacological	Non‐pharmacological (any)
Mean study size < 50 participants			
All‐cause mortality	*t*(−7.34,4.81^2^,5)	*t*(−7.84,4.60^2^,5)	*t*(−7.41,4.92^2^,5)
	Median = 0.07%	Median = 0.04%	Median = 0.07%
	IQR = 0.004% to 1%	IQR = 0.003% to 0.5%	IQR = 0.004% to 1%
	95% range = <0.0001% to 91%	95% range = <0.0001% to 76%	95% range = <0.0001% to 87%
	Pr(I^2^ < 5%) = 0.856	Pr(I^2^ < 5%) = 0.891	Pr(I^2^ < 5%) = 0.844
	*N* = 11	*N* = 11	*N* = 4
Semi‐objective	*t*(−2.81,3.21^2^,5)	*t*(−3.31,2.92^2^,5)	*t*(−2.88,3.41^2^,5)
	Median = 6%	Median = 4%	Median = 6%
	IQR = 1% to 28%	IQR = 0.8% to 16%	IQR = 0.8% to 29%
	95% range = 0.008% to 97%	95% range = 0.009% to 89%	95% range = 0.006% to 98%
	Pr(I^2^ < 5%) = 0.473	Pr(I^2^ < 5%) = 0.548	Pr(I^2^ < 5%) = 0.483
	*N* = 8	*N* = 0	*N* = 73
Subjective	*t*(−1.12,2.04^2^,5)	*t*(−1.62,1.52^2^,5)	*t*(−1.19,2.35^2^,5)
	Median = 25%	Median = 16%	Median = 24%
	IQR = 9% to 53%	IQR = 8% to 32%	IQR = 7% to 55%
	95% range = 0.6% to 94%	95% range = 1% to 80%	95% range = 0.3% to 97%
	Pr(I^2^ < 5%) = 0.157	Pr(I^2^ < 5%) = 0.161	Pr(I^2^ < 5%) = 0.195
	*N* = 149	*N* = 46	*N* = 131
Mean study size between 50 and 200 participants			
All‐cause mortality	*t*(−7.36,4.81^2^,5)	*t*(−7.87,4.60^2^,5)	*t*(−7.43,4.93^2^,5)
	Median = 0.07%	Median = 0.04%	Median = 0.07%
	IQR = 0.005% to 1%	IQR = 0.003% to 0.5%	IQR = 0.004% to 1%
	95% range = <0.0001% to 89%	95% range = <0.0001% to 74%	95% range = <0.0001% to 86%
	Pr(I^2^ < 5%) = 0.859	Pr(I^2^ < 5%) = 0.892	Pr(I^2^ < 5%) = 0.848
	*N* = 16	*N* = 25	*N* = 25
Semi‐objective	*t*(−2.83,3.23^2^,5)	*t*(−3.33,2.95^2^,5)	*t*(−2.90,3.44^2^,5)
	Median = 6%	Median = 4%	Median = 6%
	IQR = 1% to 27%	IQR = 0.8% to 16%	IQR = 0.8% to 29%
	95% range = 0.008% to 97%	95% range = 0.007% to 90%	95% range = 0.005% to 98%
	Pr(I^2^ < 5%) = 0.472	Pr(I^2^ < 5%) = 0.550	Pr(I^2^ < 5%) = 0.483
	*N* = 4	*N* = 7	*N* = 7
Subjective	*t*(−1.14,2.03^2^,5)	*t*(−1.64,1.52^2^,5)	*t*(−1.21,2.36^2^,5)
	Median = 25%	Median = 16%	Median = 23%
	IQR = 9% to 52%	IQR = 7% to 32%	IQR = 7% to 55%
	95% range = 0.5% to 94%	95% range = 1% to 79%	95% range = 0.3% to 97%
	Pr(I^2^ < 5%) = 0.160	Pr(I^2^ < 5%) = 0.171	Pr(I^2^ < 5%) = 0.202
	*N* = 51	*N* = 34	*N* = 72
Mean study size > 200 participants			
All‐cause mortality	*t*(−7.21,4.80^2^,5)	*t*(−7.71,4.59^2^,5)	*t*(−7.28,4.92^2^,5)
	Median = 0.08%	Median = 0.05%	Median = 0.07%
	IQR = 0.005% to 1%	IQR = 0.004% to 0.6%	IQR = 0.004% to 1%
	95% range = < 0.0001% to 91%	95% range = < 0.0001% to 75%	95% range = < 0.0001% to 88%
	Pr(I^2^ < 5%) = 0.850	Pr(I^2^ < 5%) = 0.888	Pr(I^2^ < 5%) = 0.839
	*N* = 15	*N* = 15	*N* = 23
Semi‐objective	*t*(−2.68,3.21^2^,5)	*t*(−3.18,2.91^2^,5)	*t*(−2.75,3.42^2^,5)
	Median = 7%	Median = 4%	Median = 6%
	IQR = 1% to 30%	IQR = 0.9% to 18%	IQR = 0.9% to 33%
	95% range = 0.01% to 97%	95% range = 0.009% to 91%	95% range = 0.006% to 98%
	Pr(I^2^ < 5%) = 0.454	Pr(I^2^ < 5%) = 0.523	Pr(I^2^ < 5%) = 0.462
	*N* = 11	*N* = 6	*N* = 6
Subjective	*t*(−0.99,2.04^2^,5)	*t*(−1.49,1.51^2^,5)	*t*(−1.06,2.37^2^,5)
	Median = 28%	Median = 18%	Median = 26%
	IQR = 10% to 56%	IQR = 9% to 35%	IQR = 8% to 58%
	95% range = 0.06% to 95%	95% range = 1% to 82%	95% range = 0.3% to 97%
	Pr(I^2^ < 5% = 0.141)	Pr(I^2^ < 5% = 0.19)	Pr(I^2^ < 5% = 0.185)
	*N* = 50	*N* = 59	*N* = 21

IQR, inter‐quartile range.

Table 6 provides predictive *t*_5_ distributions for logit(*I* ^2^) expected in future continuous outcome meta‐analyses combining SMDs. Predictive distributions for inconsistency expected among mean differences are given in the Supplementary Information Section A.4. The predictive distributions reported in this paper are for inconsistency expected across studies in 4061 (79%) continuous outcome meta‐analyses that are not related to respiratory disease or cancer. The obtained fitted distributions for $I_{\text{new}}^{2}$ in meta‐analyses related to respiratory diseases and cancer are reported in Supplementary Information Section A.4.

**Figure 7:**
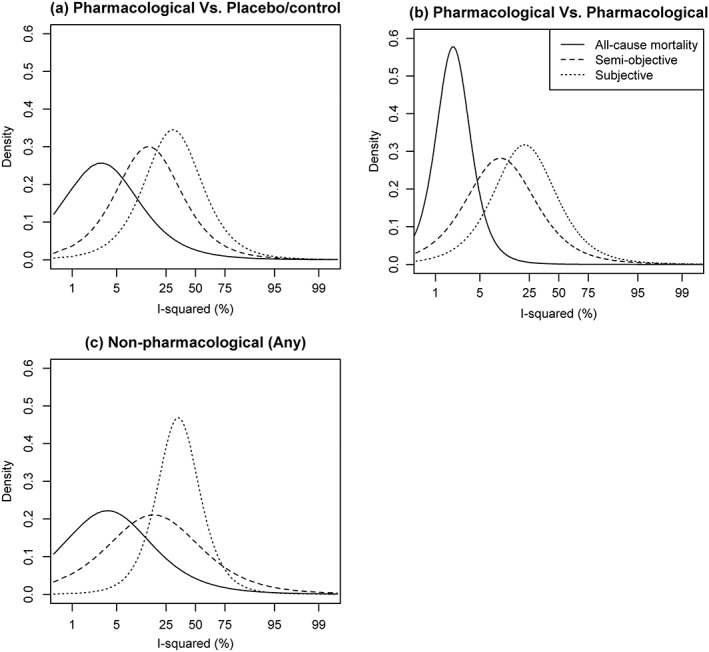
Predictive distributions for logit(*I* ^2^) expected in future binary outcome meta‐analyses combining log odds ratios, with a mean study size between 50 and 200 participants.

Discrepancies among fitted distributions for $I_{\text{new}}^{2}$ suggest differences in levels of inconsistency across meta‐analyses of different characteristics. There are notable differences across outcome types; the fitted distributions for binary and mixed outcome meta‐analyses of an all‐cause mortality outcome have much lower medians and quantiles, whilst the distributions for subjective outcomes have the highest medians and quantiles. Predictive distributions for continuous outcome meta‐analyses of obstetric outcomes and biological markers have much lower medians and 75% quantiles, with the fitted distributions for structure‐related outcomes having the highest medians and 75% quantiles. Within outcome types, differences among the three types of intervention comparisons seem small, but levels of inconsistency are consistently higher for meta‐analyses comparing a non‐pharmacological intervention. Figure 7 illustrates differences between the predictive distributions for inconsistency expected in meta‐analyses of log odds ratios, on the logit scale. These densities correspond to the fitted *t*_5_ distributions for logit( $I_{\text{new}}^{2}$) in binary outcome meta‐analyses combining studies with a mean sample size between 50 and 200 participants.

### 3.4.1 Applications to example meta‐analyses

To demonstrate the use of a predictive distribution for *I* ^2^ to inform a prior for the between‐study variance in a new meta‐analysis, we re‐analysed data from two published meta‐analyses. Each of the two examples represents the common situation where there are only a small number of studies in the meta‐analysis and Bayesian estimation is particularly beneficial. The first example includes just five studies with a mean sample size of 45.8 participants (Roqué i Figuls *et al*, 2011). This binary outcome meta‐analysis assesses radioisotopes against a placebo for relieving metastatic bone pain. The DerSimonian and Laird (DL) procedure (DerSimonian and Laird, 1986) is most commonly used to estimate the random‐effects model and is the default method in many statistical software packages. In a conventional random‐effects meta‐analysis by the DL procedure, estimates for heterogeneity are high (*τ* ^2^ = 1.06 [95 % CI : 0.11 to 20.6], *I* ^2^ = 71 %), but imprecisely estimated. The combined odds ratio is estimated as 1.98 (95% CI: 0.65 to 6.01). The confidence interval for the conventional estimate of *τ* ^2^ was obtained iteratively via the Q‐profile method (Viechtbauer, 2007).

A number of simulation studies have demonstrated that the DL procedure is likely to underestimate the between‐study variance, particularly when the number of studies is small and there is substantial heterogeneity between studies (Brockwell and Gordon, 2001, Brockwell and Gordon, 2007; Sidik and Jonkman, 2005; Sidik and Jonkman, 2007; Hartung and Makambi, 2003). When between‐study variance is underestimated, the *p*‐value for the combined intervention effect may become artificially small and the confidence bounds produced for combined intervention effects may be too narrow. For this reason, many meta‐analysts may draw false conclusions that an intervention is effective. When it is appropriate to combine studies whose estimates differ considerably, methods that better account for uncertainty than the DL procedure are recommended (Cornell *et al*, 2014). The Knapp and Hartung adjustment (Knapp and Hartung, 2003) and Bayesian modelling provide approaches that better capture the uncertainty associated with statistical heterogeneity. The use of the Knapp and Hartung adjustment leads to a considerably wider confidence interval for the combined odds ratio than that obtained using the DL method alone (Table 9).

**Table 9:** Results from re‐analysing data from published meta‐analyses using conventional and Bayesian approaches.

Radioisotopes against a placebo. Binary outcome: metastatic bone pain relief			
	Summary OR (95% CI)	${\hat{\tau }}^{2}$ (95% CI)	*I* ^2^ (95% CI)
Conventional random‐effects meta‐analysis (using DerSimonian and Laird estimation)	1.98 (0.65, 6.01)^n^	1.06 (0.11, 20.6)^n^	71% (20%, 98%)^n^
Conventional random‐effects meta‐analysis (Knapp–Hartung adjustment, based on DerSimonian and Laird estimation)	1.98 (0.35, 11.3)^n^	1.06 (0.11, 20.6)^n^	71% (20%, 98%)^n^
Bayesian random‐effects meta‐analysis with a non‐informative uniform(0,5) prior for *τ*	2.10 (0.36, 17.8)^o^	2.28 (0.14, 17.7)^o^	84% (25%, 98%)^o^
Bayesian random‐effects meta‐analysis with a non‐informative half‐normal(0,10) prior for *τ*	2.06 (0.41, 14.9)^o^	1.91 (0.12, 14.9)^o^	82% (22%, 97%)^o^
Bayesian random‐effects meta‐analysis with an informative *t*(−2.00, 0.82^2^, 5) p prior for log(*τ* ^2^)	1.96 (0.83, 4.23)^o^	0.41 (0.04, 1.81)^o^	39% (8%, 81%)^o^
Standard lower dose of risperidone against any dose. Continuous outcome: mental state			
	Summary SMD (95% CI)	${\hat{\tau }}^{2}$ (95% CI)	*I* ^2^ (95% CI)
Conventional random‐effects meta‐analysis (using DerSimonian and Laird estimation)	0.61 (0.19, 1.03)^n^	0.14 (0.02, 3.73)^n^	76% (31%, 99%)^n^
Conventional random‐effects meta‐analysis (Knapp–Hartung adjustment, based on DerSimonian and Laird estimation)	0.61 (−0.18, 1.39)^n^	0.14 (0.02, 3.73)^n^	76% (31%, 99%)^n^
Bayesian random‐effects meta‐analysis with a non‐informative uniform(0,5) prior for *τ*	0.61 (−0.55, 1.64)^o^	0.39 (0.02, 8.67)^o^	90% (28%, 99.5%)^o^
Bayesian random‐effects meta‐analysis with a non‐informative half‐normal(0,10) prior for *τ*	0.61 (−0.46, 1.57)^o^	0.36 (0.01, 6.15)^o^	89% (25%, 99%)^o^
Bayesian random‐effects meta‐analysis with an informative *t*(−2.88, 1.70^2^, 5) q prior for log(*τ* ^2^)	0.63 (0.11, 1.07)^o^	0.10 (0.004, 0.87)^o^	70% (8%, 95%)^o^

OR, odds ratio; SMD, standardized mean difference.

n 95% confidence interval. For *τ* ^2^, this is obtained iteratively via the Q‐profile method (Viechtbauer, 2007). The interval for *I* ^2^ is obtained by monotonic transformation of *τ* ^2^ (Higgins and Thompson (2002)).

o Posterior medians and 95% credible intervals are reported.

p Predictive distribution for log(*τ* ^2^) based on the “typical” within‐study variance ${\hat{\sigma }}^{2}=0.42$ and the predictive *t*(−1.14, 0.82^2^, 5) distribution for logit(*I* ^2^) for a non‐pharmacological binary outcome meta‐analysis with a subjective outcome.

q Predictive distribution for log(*τ* ^2^) based on the “typical” within‐study variance ${\hat{\sigma }}^{2}=0.04$ and the predictive *t*(0.25, 1.70^2^, 5) distribution for logit(*I* ^2^) for a pharmacological vs pharmacologicalcontinuous outcome meta‐analysis assessing mental health.

Results for performing Bayesian random‐effects meta‐analysis with non‐informative priors for the between‐study heterogeneity are provided in Table 9. As a non‐informative prior for the between‐study standard deviation *τ*, we applied a uniform(0, 5) prior, as recommended by Spiegelhalter *et al* (2004). We also used a positive half‐normal(0,10) prior for *τ*, which has been used in previous applications to meta‐analysis (Thompson *et al*, 1997). Clearly, the between‐study variance *τ* ^2^ is estimated subject to substantial uncertainty in each case, and this is reflected by the considerably wide intervals for the summary intervention effect. The example binary outcome meta‐analysis compares a non‐pharmacological intervention against a placebo with respect to a subjective outcome. Using a *t*(−1.14, 0.82^2^, 5) distribution as an informative prior for logit(*I* ^2^) (and hence a log *t*(−2.00, 0.82^2^, 5) prior for *τ* ^2^, based on the “typical” within‐study variance ${\hat{\sigma }}^{2}=0.42$), the central estimates for both *I* ^2^ and *τ* ^2^ decrease. Evidently, the corresponding 95% CIs for the summary OR and the between‐study heterogeneity variance are narrower in comparison with those obtained using conventional methods for random‐effects meta‐analysis.

As a contrasting example, we re‐analysed data from a continuous outcome meta‐analysis combining four studies to assess the standard lower dose of risperidone in comparison with any dose with respect to the mental state of schizophrenia patients (Li et al., 2009). This meta‐analysis has a mean study size of 103 patients. Symptom severity was measured using a Positive and Negative Syndrome Scale score where a reduction in Positive and Negative Syndrome Scale score represents an improvement in mental state. In a conventional random‐effects meta‐analysis by the DL method combining standardized mean differences, the heterogeneity estimates are high and again imprecisely estimated (*τ* ^2^ = 0.14 [95 % CI : 0.02 to 3.73], *I* ^2^ = 76 %). The use of the Knapp and Hartung adjustment shows a somewhat wider confidence interval for the combined SMD. Bayesian meta‐analysis using an informative logit *t*( 0.25, 1.70^2^, 5) prior for *I* ^2^ (and hence a log *t*(−2.88, 1.70^2^, 5) prior for *τ* ^2^, based on the “typical” within‐study variance ${\hat{\sigma }}^{2}=0.04$) leads to a slightly reduced estimate for the between‐study variance of 0.10 and a noticeably narrower 95% credible interval (0.004 to 0.87). Although the central estimate for *τ* ^2^ has reduced only slightly from the conventional estimate of 0.14, the 95% interval for the combined SMD has widened because the Bayesian approach accounts for the uncertainty in *τ* ^2^.

The Bayesian approaches implementing informative priors for *I* ^2^ incorporate our beliefs about the likely inconsistency across studies in the meta‐analysis. Therefore, we consider these results to be more appropriate than those obtained using conventional methods for meta‐analysis. The basic code to perform each of the example Bayesian random‐effects meta‐analyses, using informative priors for inconsistency, is available in Supplementary Information Section A.6, together with the study data. Code is available to perform binary outcome meta‐analysis on the log OR, log RR or RD scale and to undertake continuous outcome meta‐analysis on the SMD or mean difference scale.

## 4 Discussion

This paper has presented an empirical investigation of 3873 binary outcome, 5132 continuous outcome and 880 mixed outcome meta‐analyses from the CDSR. Our investigation has demonstrated that analyses may exhibit contrasting levels of inconsistency according to the type of outcome data and the scale on which the meta‐analysis is performed.

Measures for binary outcomes have already been compared in terms of the statistical significance of heterogeneity in a pair‐wise meta‐analysis. Engels *et al* (2000) compared analyses of odds ratios and risk differences in 125 meta‐analyses, and Deeks (2002) additionally explored heterogeneity among relative risks in 551 meta‐analyses. In our work, we computed the scale‐invariant *I* ^2^ statistics for a very large collection of published meta‐analyses. Our analyses allowed us to compare outcome measures with respect to consistency across studies. We have shown that the analyses of odds ratios and relative risks exhibit similar levels of inconsistency on average, with analyses of risk differences yielding higher levels of inconsistency. These findings confirm the comparisons of binary outcome measures by Engels *et al* and Deeks. Our results show a number of cases where an *I* ^2^ estimate close to zero based on one metric corresponds to an extremely large *I* ^2^ estimate based on an alternative metric. In situations where inconsistency among risk differences is substantially different to inconsistency among relative risks or odds ratios, there are likely to be high discrepancies among the study sizes and event rates (Engels *et al*, 2000). Likewise, differences between odds ratios and relative risks are likely to occur, if there is a wide range of absolute event rates. This is particularly important in network meta‐analyses comparing three or more interventions, where more varied absolute risks might be expected, because of the greater number of included interventions.

To our knowledge, this paper represents the first empirical investigation of inconsistency across studies in continuous outcome meta‐analyses. Friedrich *et al* (2008) used simulated data sets to assess the performance characteristics of the mean difference and SMD. Our findings corroborate those of Friedrich *et al*, which suggest that mean differences are generally as consistent as SMDs. Nonetheless, there is evidence to suggest that, in some situations, *I* ^2^ might be reduced substantially by using the alternative scale. As a possible explanation for this, Friedrich *et al* (2008) showed that the mean difference has minimal bias, whereas the SMD is biased towards zero (no intervention effect) when the number of participants in each study is small. When the SMD is biased, *I* ^2^ is smaller than in the mean difference‐based analysis because the bias decreases the weighting of the studies with effects further away from zero, reducing *I* ^2^. Under scenarios with less bias, the simulation study showed *I* ^2^ values to compare favourably between metrics.

The selection of an outcome measure for meta‐analysis depends on balancing three factors (Deeks 2002). First, we would like an outcome measure that gives similar results for all studies included in the meta‐analysis. When there is substantial variation among study results and there is inconsistency in the direction of effect, it would be misleading to give an average estimate for the intervention effect, particularly if study designs, within‐study biases, and reporting biases are not taken into account (Turner *et al*, 2009; Welton *et al*, 2009; Dwan *et al*, 2013). Second, it is important that the outcome measure has the mathematical properties required for conducting a sound meta‐analysis. Third, it would be desirable to use an outcome measure that is easy for meta‐analysts to interpret and compute. For binary data analysis, there is no single metric that is best for all criteria, and the choice of an outcome measure therefore involves a trade‐off. We recommend performing a sensitivity analysis to investigate whether choice of effect metric influences the conclusions of the meta‐analysis. We suggest selecting the outcome metric for analysis on the basis of homogeneity and mathematical properties alone and then using empirical evidence on heterogeneity to compute results on the scale desired for interpretation (van Valkenhoef and Ades, 2013; Dias *et al*, 2013). Choice of an outcome measure is especially important in network meta‐analysis, because it can affect the ranking of treatments (Norton *et al*, 2012). Caldwell *et al* (2012) discussed selecting the scale of measurement in network meta‐analysis and showed that the larger evidence base in such analyses may enable a data driven approach to selecting the scale.

This research set out to compare consistency across studies in meta‐analyses using various types of outcome data. We have demonstrated that analyses of relative measures for binary data and analyses of mixed outcome data tend be more consistent than analyses of continuous data. We have analysed data from continuous outcome meta‐analyses assessing an obstetric outcome for which we could calculate the RoM for each individual study. Our results suggest that the logarithm of RoM compares favourably with the mean difference and SMD in terms of inconsistency among results of included studies in a meta‐analysis. However, these findings are limited by the normal approximation to the logarithm of RoM, which may not be entirely appropriate. It is therefore unclear whether the observed high levels of inconsistency are associated with the use of difference measures as opposed to relative measures. A possible explanation may be that continuous data analysis is more susceptible to errors in the data. For instance, the standard error and standard deviation are often confused. Further empirical research could explore this issue by manually searching published articles and noting the inappropriate use of the term “standard error”, in a similar way to Nagele (2003).

A secondary objective of this work was to facilitate the incorporation of external evidence on heterogeneity in meta‐analysis. This paper has provided sets of predictive distributions for *I* ^2^ in a wide range of specific research settings, which can be used directly to inform priors for the between‐study heterogeneity variance in future meta‐analyses of binary, continuous and mixed outcomes. The distributions would be very useful in future meta‐analyses including only a small number of studies. We have demonstrated how a predictive distribution for *I* ^2^ can be implemented in a Bayesian random‐effects meta‐analysis, in order to inform a prior for the between‐study variance *τ* ^2^. In each of the two examples, the precision of heterogeneity improved with use of an informative prior for *τ* ^2^. We note that without validation that the coverage rate of the credible interval for heterogeneity is closer to optimal, the narrower intervals obtained do not necessarily mean that the estimation is more accurate. A simulation study could inform this question in future work.

In other work, Turner *et al* (2012) have suggested informative priors for the between‐study variance *τ* ^2^ for use in binary outcome meta‐analyses on the log odds ratio scale, and Rhodes *et al* (2015) have reported priors for continuous outcome meta‐analyses using the SMD scale. The present study provides predictive distributions for *I* ^2^ that would serve to provide informative prior distributions for *τ* ^2^ in binary outcome meta‐analyses using the log OR, log RR and RD scales, continuous outcome meta‐analyses using the SMD and mean difference scales, and mixed outcome meta‐analyses. The discrepancies between our priors across different settings reflect the associations between meta‐analysis characteristics and heterogeneity identified by Turner *et al* and Rhodes *et al.* The implications are that Bayesian meta‐analysis with an informative prior for *I* ^2^ should lead to comparable inference to meta‐analysis assigning an informative prior to *τ* ^2^. We recommend to assign a prior distribution directly to *τ* ^2^, where available, because this parameter is used in the analysis. Otherwise, for example, in meta‐analyses of mean differences, a predictive distribution for *I* ^2^ can be used to inform a prior for *τ* ^2^. We recommend using the predictive distributions for specific research settings if the new meta‐analysis fits directly into the category. In situations where the new meta‐analysis is suited to a number of different research settings, we suggest that the predictive distribution reported for a general healthcare setting may be an appropriate prior.

The findings of this paper are subject to the limitations of *I* ^2^ as a measure of heterogeneity. In our analyses, we estimated *I* ^2^ using the definition *τ* ^2^/(*τ* ^2^ + *σ* ^2^). We applied this equation to the general case, by replacing *σ* ^2^ by an estimate of the “typical” within‐study variance in the same way as Higgins and Thompson (2002). In our exploratory data analyses, we used *I* ^2^ values derived from the method of moments‐based estimates of the between‐study variance *τ* ^2^. The derivation of the method of moments estimate of *τ* ^2^, which is part of the DerSimonian and Laird procedure, is dependent on the unknown true within‐study variances from each study. This estimate of *τ* ^2^ can have non‐trivial negative bias (Böhning *et al*, 2002; Hamza *et al*, 2008), and so the *I* ^2^ values obtained in this way may be smaller than they should be. Furthermore, Wetterslev *et al* (2009) have criticised *I* ^2^ because of the dependency on an estimate for a “typical” within‐study variance. Estimation of the “typical” within‐study variance may mislead, because it gives less emphasis to larger studies with larger numbers of events. If the “typical” within‐study variance overestimates the sampling error then *I* ^2^ will be underestimated and vice versa. Another limitation of our analyses is that they did not account for uncertainty in the estimate of the “typical” within‐study variance, which we treated as fixed and known.

A further limitation of *I* ^2^ concerns the dependency of the statistic on within‐study precisions. Our initial descriptive analyses did not allow for this dependency, and our formal statistical analyses using a Bayesian framework only crudely adjusted for it. If the included studies are very large such that the within‐study variances are very small, then even though the estimate for the between‐study heterogeneity variance *τ* ^2^ may be small, the estimate for *I* ^2^ could be large. In view of the limitations of *I* ^2^, Higgins and Thompson (2002) recommend using *τ* ^2^ to best describe the underlying between‐study variation in a meta‐analysis. *I* ^2^ essentially compares the estimated value of *τ* ^2^ with the “typical” within‐study variance ${\hat{\sigma }}^{2}$ (Higgins and Thompson, 2002), which is dependent on the design characteristics of included studies. For this reason, *I* ^2^ cannot be used to measure the extent of the between‐study heterogeneity in a meta‐analysis. In this paper, focus on the *I* ^2^ statistic was useful to compare different types of outcome data and outcome measures in terms of consistency across studies in a meta‐analysis. Such comparisons could not be made using only the between‐study variance *τ* ^2^ that is defined on the outcome metric scale.

A number of caveats need to be mentioned regarding the use of our informative priors for *I* ^2^. First, for computational convenience, we classified meta‐analyses into just two or three categories for mean study size. Guidelines are needed for use of our priors in practise, for example, what to recommend for a meta‐analysis combining studies with a large mean sample size greater than say 10 000 participants. Second, we note that our analyses include insufficient data for certain types of meta‐analyses such as continuous outcome meta‐analyses for cancer and pharmacological versus pharmacological meta‐analyses of mixed outcomes examining a semi‐objective outcome (Supplementary Information Section A.5). In case there is no sufficient data on a similar outcome, intervention comparison or therapeutic area, we suggest considering several prior distributions as sensitivity analysis. In addition to using the prior for the specific meta‐analysis setting, an analyst could apply the prior for a general research setting. Where no relevant data‐based prior is available, it would be possible to use elicited opinion from experts to construct an informative prior for inconsistency among studies in the meta‐analysis. For example, Turner *et al* (2009) used elicited opinion to construct prior distributions representing biases in each study and perform a bias‐adjusted meta‐analysis.

The data set includes only meta‐analyses in Cochrane reviews. These include a wide range of application areas but may not be representative of all healthcare meta‐analyses. Automated data extraction was used to obtain the data from each meta‐analysis in the CDSR. For this reason, a limitation of our work is that the data set only includes data that were entered numerically in tables or figures by the Cochrane review authors, and meta‐analyses reported only in the main text are excluded. This could cause us to underestimate the true levels of heterogeneity, because meta‐analyses described in the main text alone may tend to include more heterogeneous studies. On the other hand, we included data sets in which authors had chosen not to combine the study data numerically, so our priors might support higher values of between‐study variance than would apply to many syntheses in practise. Another limitation is that the classifications of meta‐analysis characteristics were extremely time‐consuming and were therefore undertaken by just one person.

In summary, levels of inconsistency among results of included studies were found to be sensitive to the type of outcome data used and the scale on which the meta‐analysis is performed. Predictive distributions for inconsistency would be useful to inform priors for the between‐study variance in meta‐analyses including few studies (particularly for continuous outcome meta‐analysis on the mean difference scale where we do not have “off the shelf” prior distributions for the between‐study variance). For meta‐analysis of binary and continuous outcome data, our investigation provides some guidance on which outcome measures are likely to be most consistent in particular settings.

## Supporting information

Supporting info item
