# Supplementary material for: Empirical evidence about inconsistency among studies in a pair‐wise meta‐analysis
Source: Res Synth Methods. 2015 Dec 17;7(4):346–70. doi: 10.1002/jrsm.1193 (PMC5217093; doi:10.1002/jrsm.1193)
Supplement: Supplementary file 1 — Supporting info item [file JRSM-7-346-s001.pdf]

# A Appendix (Supplementary material)

## A.1 Statistical analysis

### A.1.1 Effect measures for binary outcomes

For binary outcome data, results of each study indexed $i$ include the number of participants with each outcome (event or no event) in each intervention group (C or T), and the total number of participants in each intervention group (Table A1).

| | Event | No event | Total |
|---|---|---|---|
| Control | $r_i^C$ | $(n_i^C - r_i^C)$ | $n_i^C$ |
| Intervention | $r_i^T$ | $(n_i^T - r_i^T)$ | $n_i^T$ |

Table A1: Binary outcome data for study $i$

The odds ratio (OR) is given by

$$OR_i = \frac{\text{odds of event in intervention group}}{\text{odds of event in control group}} = \frac{r_i^T/(n_i^T - r_i^T)}{r_i^C/(n_i^C - r_i^C)}.$$

The standard error of the log OR is

$$SE(\log(OR_i)) = \sqrt{\frac{1}{r_i^T} + \frac{1}{r_i^C} + \frac{1}{(n_i^T - r_i^T)} + \frac{1}{(n_i^C - r_i^C)}}.$$

The relative risk (RR) is

$$RR_i = \frac{\text{risk of event in intervention group}}{\text{risk of event in control group}} = \frac{r_i^T/n_i^T}{r_i^C/n_i^C}.$$

The standard error of the log RR is

$$SE(\log(RR_i)) = \sqrt{\frac{1}{r_i^T} + \frac{1}{r_i^C} - \frac{1}{n_i^T} - \frac{1}{n_i^C}}.$$

The risk difference (RD) is

$$RD_i = \text{risk of event in intervention group} - \text{risk of event in control group} = \frac{r_i^T}{n_i^T} - \frac{r_i^C}{n_i^C},$$

with standard error

$$SE(RD_i) = \sqrt{\frac{r_i^T(n_i^T - r_i^T)}{(n_i^T)^3} + \frac{r_i^C(n_i^C - r_i^C)}{(n_i^C)^3}}.$$

Where zeros cause problems with computation of odds ratios and relative risks or their standard errors, 0.5 is added to all cells ($r_i^C$, $(n_i^C - r_i^C)$,$r_i^T$, $(n_i^T - r_i^T)$) which is usually satisfactory (Gart *et al*, 1985). Except where $r_i^C = r_i^T = 0$ or $(n_i^C - r_i^C) = (n_i^T - r_i^T) = 0$, in which case the odds ratio and relative risk are undefined. In Bayesian approaches that incorporate heterogeneity, continuity corrections are not required.

### A.1.2 Fitted hierarchical model for continuous data

Before describing the mathematical form of the models fitted to continuous outcome data, we define the outcome measures used for analysing continuous data. For continuous outcome data, results of each study include the number of participants in each of the two intervention arms, and their mean response and standard deviation of their mean responses (Table A2).

| Study $i$ | Mean response | Standard deviation | No. of participants |
|---|---|---|---|
| Control | $m_i^C$ | $sd_i^C$ | $n_i^C$ |
| Intervention | $m_i^T$ | $sd_i^T$ | $n_i^T$ |

Table A2: Continuous outcome data for study $i$

Denote the pooled standard deviation across the two intervention groups by $s_i$:

$$s_i = \sqrt{\frac{(n_i^C - 1)(sd_i^C)^2 + (n_i^T - 1)(sd_i^T)^2}{N_i - 2}}$$

where $N_i = n_i^C + n_i^T$.

The mean difference is given by

$$MD_i = m_i^T - m_i^C,$$

with standard error

$$SE(MD_i) = \sqrt{\frac{(sd_i^T)^2}{n_i^T} + \frac{(sd_i^C)^2}{n_i^C}}.$$

The standardized mean difference (SMD) is

$$SMD_i = \frac{MD_i}{s_i}\left(1 - \frac{3}{4N_i - 9}\right),$$

with standard error

$$SE(SMD_i) = \sqrt{\frac{N_i}{n_i^C n_i^T} + \frac{SMD_i^2}{2(N_i - 3.94)}}.$$

The ratio of means (ROM) is given by

$$ROM_i = \frac{m_i^T}{m_i^C}.$$

The standard error of the log OR is

$$SE(\log(ROM_i)) = \sqrt{\frac{1}{n_i^T}\left(\frac{sd_i^T}{m_i^T}\right)^2 + \frac{1}{n_i^C}\left(\frac{sd_i^C}{m_i^C}\right)^2}.$$

The statistical models fitted to study data from continuous outcome meta-analyses differed in a couple of respects from those fitted to the binary outcome data. For meta-analysis of binary outcome data, we used the binomial likelihood approach which is preferable in principle (Hamza *et al*, 2008). When analysing continuous data, we assume normality of observed study-level effects, because we do not have patient-level data. Our binary data analyses included one meta-analysis per intervention comparison, whereas continuous and mixed outcome meta-analyses were nested within intervention comparisons. The hierarchical models used to analyse these study data allowed inconsistency to vary across meta-analyses within comparisons and to vary across comparisons.

We provide the mathematical form of the statistical models fitted to study data from all continuous outcome meta-analyses in the data set. Within each meta-analysis with outcome $j$ and comparison $k$, a random-effects model with normal within-study likelihoods was fitted to outcome data $y_{kji}$ from each study $i$:

$$\begin{aligned} y_{kji} &\sim N(\theta_{kji}, \sigma^2_{kji}) \\ \theta_{kji} &\sim N(\mu_{kj}, \tau^2_{kj}), \\ \log(\tau^2_{kj}) &= \text{logit}(I^2_{kj}) + \log(\hat{\sigma}^2_{kj}), \end{aligned}$$

where $\hat{\sigma}^2_{kj}$ is a "typical" within-study variance for the meta-analysis, computed using the observed within-study variances $\sigma^2_{kji}$ in study $i$. In the defined model, $\theta_{kji}$ denotes the underlying intervention effect for the $i$-th study within outcome $j$ within comparison $k$, $\mu_{kj}$ is the combined intervention effect for a meta-analysis with outcome $j$ and comparison $k$ and $\tau^2_{kj}$ is the corresponding between-study heterogeneity variance.

In an earlier paper, we fitted a hierarchical regression model to underlying values of $\log(\tau^2_{kj})$ across continuous outcome meta-analyses, assuming a $t$ distribution with 5 degrees of freedom for the residual variation (Rhodes *et al*, 2015). Here, the hierarchical regression model was fitted to underlying values of $\text{logit}(I^2_{kj})$, with the inclusion of additional indicators of mean study size as covariates. In the model below, $x_{1kj}, ..., x_{8kj}$ are indicators for the type of outcome examined by meta-analysis with outcome $j$ within comparison $k$. After adjustment for other predictors of inconsistency in the model, regression coefficients represent average differences in $I^2$ on the logit scale among meta-analyses of different characteristics. Fixed effects $\beta_1, ..., \beta_7$ estimate average differences among each outcome type and the reference group of general health-related outcomes, whilst the error terms $e_1, ..., e_8$ allow for residual variation across meta-analyses with separate variances $\phi^2_1, ..., \phi^2_8$ assumed for each outcome type. Similarly $z_{1k}, z_{2k}, z_{3k}$ are binary indicators for the type of intervention comparison under comparison $k$. Fixed effects $\gamma_1$ and $\gamma_2$ estimate average differences each intervention comparison type and the reference group of meta-analyses comparing a non-pharmacological intervention, while the random effects $u_1, u_2, u_3$ allow for variability across comparisons, with separate variances $\kappa^2_1, \kappa^2_2, \kappa^2_3$ assumed for each intervention comparison type. We denote $s_{1kj}$ to be an indicator of mean study size fewer than 50 participants, with $\xi_1$ estimating the average difference between meta-analyses with mean sample size fewer than 50 and those meta-analyses with greater mean sample sizes. Covariates $a_{1kj}, ..., a_{10kj}$ are indicators for therapeutic areas, with fixed effects $\delta_1, ..., \delta_{10}$ estimating average differences between each therapeutic area and the reference group of meta-analyses related to cardiovascular disease, for which the mean estimate of $I^2$ was central across therapeutic areas.

The logit-$t$ model fitted to $I^2_{kj}$ was:

$$\text{logit}(I^2_{kj}) = \alpha_k + \beta_1 x_{1kj} + ... + \beta_7 x_{7kj} + \xi_1 s_{1kj} + \sum_{p=1}^{10} \delta_p a_{pkj} + \sum_{l=1}^{8} e_{lkj} x_{lkj}$$

$$\text{and } \alpha_k = \alpha + \gamma_1 z_{1k} + \gamma_2 z_{2k} + \sum_{m=1}^{3} u_{mk} z_{mk},$$

where $e_{lkj} \sim t(0, \phi^2_l, 5)$ and $u_{mk} \sim t(0, \kappa^2_m, 5)$ for $l = 1, 2, ..., 8$ and $m = 1, 2, 3$.

Similar statistical models were fitted to the study data from all mixed outcome meta-analyses in the data set. The only differences between the models were the covariates representing meta-analysis characteristics. To recall, meta-analyses were categorised into groups by outcome type and mean study size, according to the type of outcome data (See Data description in Methods section).

### A.1.3 WinBUGS code to fit a simple hierarchical model

We provide the WinBUGS code to fit a simple hierarchical model to full study-level data, according to Appendix A.1.2, estimating variation in inconsistency without adjustment for meta-analysis characteristics as covariates.

```
model {
     for (i in 1:N) {
          # y and v are the study-specific intervention effects and variances
          y[i]~dnorm(theta[i],prec.y[i])
          prec.y[i]<-1/v[i]
          # Random-effects model within meta-analyses
          theta[i] ~ dnorm(mu[ma[i]],invtausq[ma[i]])
          }
          for (m in 1:M) {
               mu[m] ~ dnorm(0,0.1)
               invtausq[m] <- 1 / tausq[m]
               # ssq are the fixed "typical" within-study variances
               tausq[m] <- (ssq[m]*isq[m])/(1-isq[m])
               isq[m] <- exp(logit.isq[m]) / (1 + exp(logit.isq[m]))
               logit.isq[m] ~ dt(amongma.mu[comparison[m]],amongma.prec,5)
               }
          for (j in 1:C) {
               amongma.mu[j] ~ dt(mu.all,amongcomp.prec,5)
               }

          # Priors for unknown parameters

          mu.all ~ dnorm(0,0.1)
          amongma.prec ~ dgamma(0.1,0.1)
          amongcomp.prec ~ dgamma(0.1,0.1)

          # Obtain a predictive distribution for I-squared expected in a new meta-analysis
          logitisq.new ~ dt(amongma.mu.new,amongma.prec,5)
          amongma.mu.new ~ dt(mu.all,amongcomp.prec,5)
          isq.new <- exp(logit.isq.new)/(1+exp(logit.isq.new))
 }
```

## A.2 Method-of-moments estimates for $I^2$

For each type of outcome data, a histogram representing the empirical distribution of positive estimates for $I^2$ on the logit scale is provided in Figure A1. Each histogram representing the empirical distribution of positive estimates for logit($I^2$) shows $I^2$ values that may be considered as outliers. For example, among the binary outcome meta-analyses, just 23 (0.6%) have an $I^2$ statistic greater than 92%. In the lower tail of the distribution, there are 17 meta-analyses (0.04%) with a positive $I^2$ statistic less than 2%.

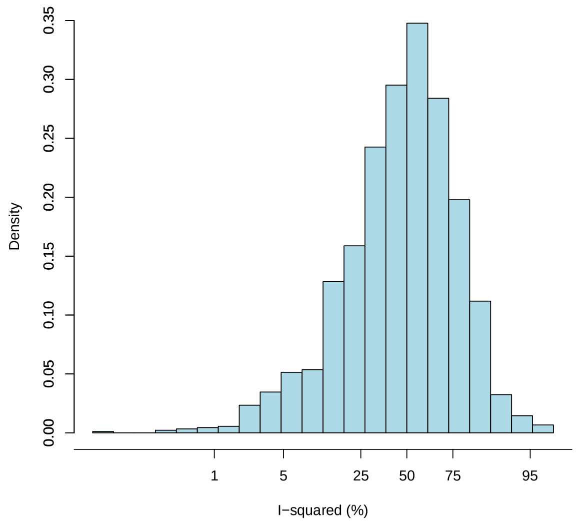


(a) Binary outcome meta-analyses of log odds ratios

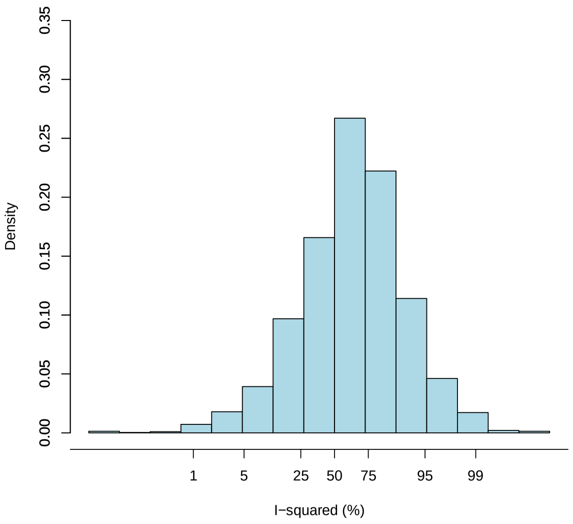


(b) Continuous outcome meta-analyses of SMDs

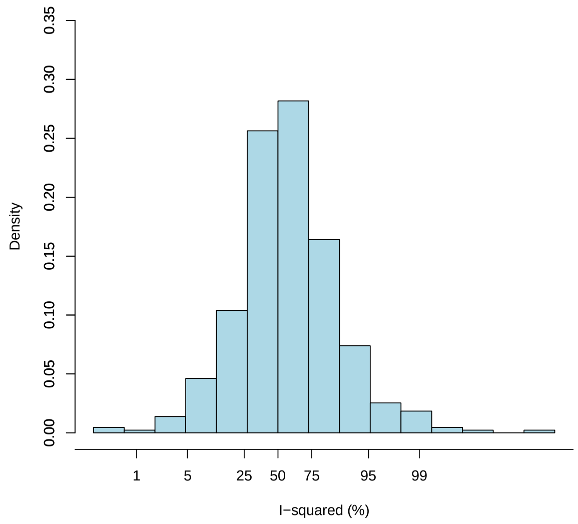


(c) Mixed outcome meta-analyses

Figure A1: Distribution of non-zero estimates for $I^2$, plotted on the logit scale.

| Outcome data | Scale | Min | Median | Max | IQR | 80% range | 90% range | 95% range | 97.5% range |
|---|---|---|---|---|---|---|---|---|---|
| Binary | Log odds ratio | 0.1% | 50% | 97% | 28% to 68% | 13% to 80% | 6% to 85% | 4% to 88% | 2% to 92% |
| Continuous | SMD | 0.1% | 66% | 99.9% | 40% to 84% | 17% to 93% | 9% to 96% | 4% to 98% | 2% to 99% |
| Mixed | | 0.3% | 58% | 99.99% | 35% to 77% | 17% to 90% | 11% to 96% | 6% to 98% | 3% to 99% |

Table A3: Summary statistics for non-zero method-of-moments based estimates for $I^2$.

### A.2.1 Quantile-quantile plots comparing the distributions of method-of-moments estimates for $I^2$ across outcome types

Empirical quantile-quantile plots that compare the distributions for the three types of outcome data are displayed in Figure A2. $I^2$ values based on analyses of binary data appear to be considerably lower than the $I^2$ values for continuous outcome meta-analyses, and also lower than the $I^2$ values for mixed outcome meta-analyses.

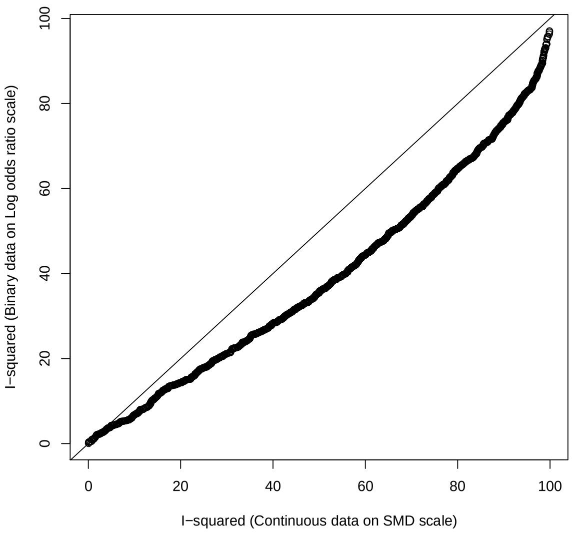


(a) Binary outcome meta-analyses of log odds ratios vs continuous outcome meta-analyses of SMDs

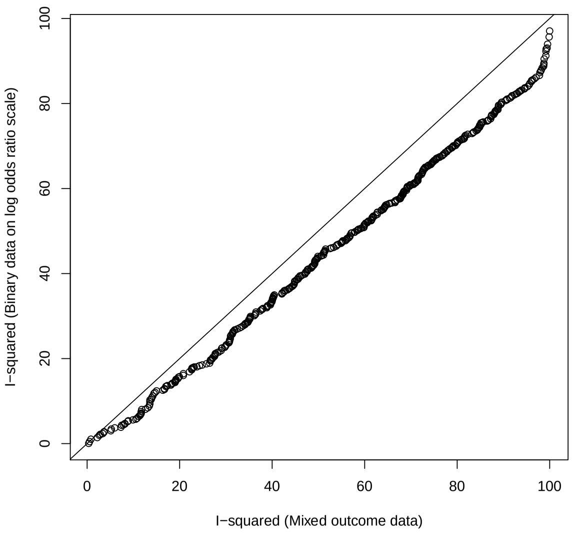


(b) Binary outcome meta-analyses of log odds ratios vs mixed outcome meta-analyses

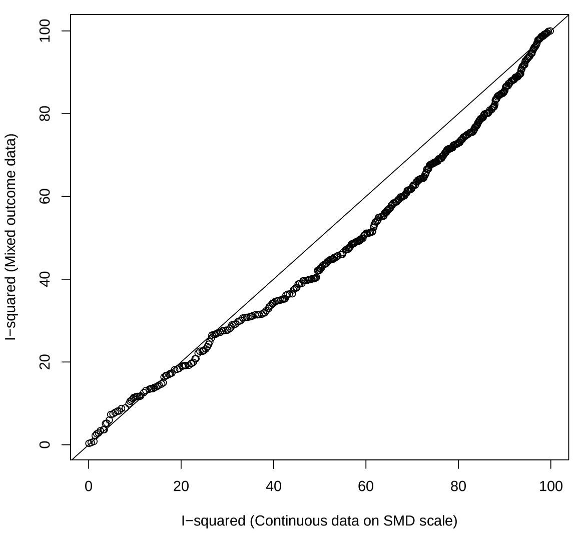


(c) Mixed outcome meta-analyses vs continuous outcome meta-analyses of SMDs

Figure A2: Empirical quantile-quantile plots comparing the distributions of method-of-moments estimates for $I^2$ for the three different types of outcome data.

## A.3 Model comparison

We report results based on analyses of binary outcome data on the log odds ratio scale. To explore the appropriateness of the candidate distributions for $I^2$, we used WinBUGS to produce a pseudo-value for $I^2$, for each meta-analysis in the data set. To derive these values, we fixed hyper parameters relating to $I^2$ to their fitted values and ran a single update of the Gibbs sampler. We then visually compared the histograms of these pseudo-values to the empirical distribution of method-of-moments based estimates for $I^2$ (Figure A3). In conventional random-effects meta-analysis, estimates of between-study variance $\tau^2$, and hence $I^2$, are often zero (Chung *et al*, 2013). Similarly, we might expect a high proportion of Bayesian estimates for $I^2$ to be small, and we would like a distribution for $I^2$ to allow for this.

The histograms of pseudo-values for $I^2$ based on the logit-$t_5$ and logit-normal models for $I^2$ are most similar to the empirical distribution of method-of-moments based estimates for $I^2$. These distributions all show a peak in $I^2$ estimates close to the boundary.

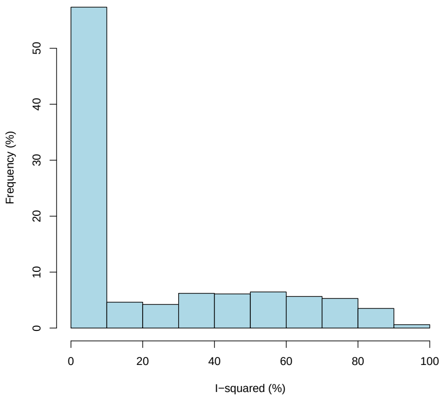


(a) Method-of-moments estimates

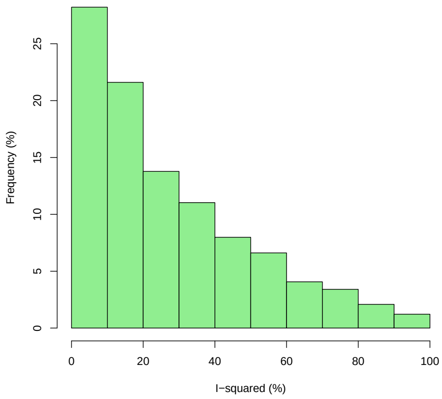


(b) Logit-$t_5$ model

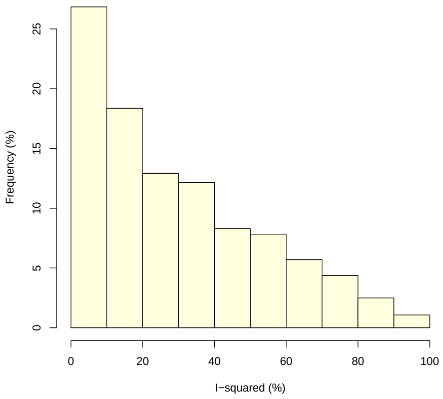


(c) Logit-normal model

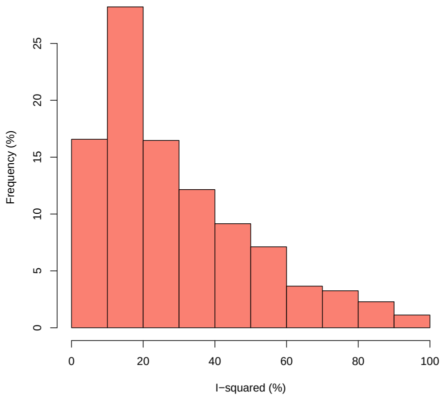


(d) Inverse-gamma model for $I^2/(1-I^2)$

Figure A3: (a) Empirical distribution of method-of-moments based estimates for $I^2$. (b)-(d) Histograms of pseudo-values for $I^2$, under the fitted Bayesian models without covariates for meta-analysis characteristics.

After adjusting for meta-analysis characteristics as covariates, using a logit-normal model for $I^2$ led to a DIC of 226541, compared with 226544 for the inverse-gamma model for $I^2/(1-I^2)$ (implying an inverse-gamma distribution for $\tau^2$). Using a logit-$t$ distribution, with 5 degrees of freedom, for $I^2$ led to a DIC of 226028. Thus, the $t_5$ model for logit($I^2$) appears to be the better choice.

We assessed the fit of the logit-$t_5$ model for $I^2$ using a quantile-quantile plot of posterior medians of $I^2$ versus the fitted logit-$t_5$ distribution (Figure A4). The approximate linearity of the points suggests that the $I^2$ data conform well to a logit-$t_5$ distribution.

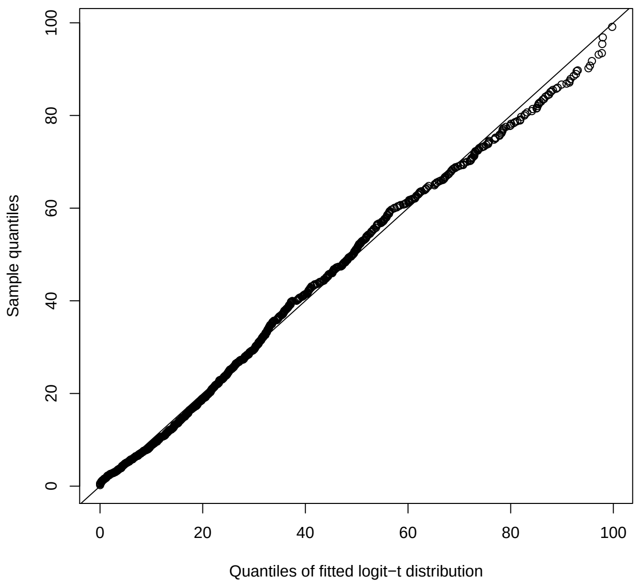


Figure A4: A quantile-quantile plot of posterior medians of $I^2$ versus the fitted logit-$t_5$ distribution.

## A.4 Predictive distributions for inconsistency in future meta-analyses

We present predictive distributions for logit($I^2$) expected in future binary outcome meta-analyses using the log relative risk in Table A4. The fitted distributions for binary outcome meta-analyses of risk differences are given in Table A5. In addition we report predictive distributions for logit($I^2$) expected in future continuous outcome meta-analyses. For therapeutic areas of respiratory disease and cancer, the predictive distributions for inconsistency among SMDs are provided in Table A6 and A7 respectively. Predictive distributions for logit($I^2$) in future continuous outcome meta-analyses using the mean difference scale are reported in Table A8, A9 and A10.

**Mean study size < 50 participants**

| | Pharmacological Vs. Placebo/ Control | Pharmacological Vs. Pharmacological | Non-Pharmacological (Any) |
|---|---|---|---|
| **All-cause mortality** | $t(-4.25, 1.56^2, 5)$<br>Median = 2%<br>IQR = 1% to 3%<br>95% range = 0.05% to 20%<br>$Pr(I^2 < 5\%) = 0.849$<br>$N = 39$ | $t(-4.88, 0.75^2, 5)$<br>Median = 1%<br>IQR = 0% to 1%<br>95% range = 0.2% to 3%<br>$Pr(I^2 < 5\%) = 0.994$<br>$N = 17$ | $t(-4.19, 1.84^2, 5)$<br>Median = 2%<br>IQR = 1% to 4%<br>95% range = 0.02% to 33%<br>$Pr(I^2 < 5\%) = 0.792$<br>$N = 29$ |
| **Semi-objective** | $t(-2.62, 1.55^2, 5)$<br>Median = 7%<br>IQR = 3% to 14%<br>95% range = 0.3% to 60%<br>$Pr(I^2 < 5\%) = 0.392$<br>$N = 70$ | $t(-3.20, 1.63^2, 5)$<br>Median = 4%<br>IQR = 2% to 9%<br>95% range = 0.1% to 50%<br>$Pr(I^2 < 5\%) = 0.578$<br>$N = 29$ | $t(-2.52, 1.86^2, 5)$<br>Median = 7%<br>IQR = 3% to 19%<br>95% range = 0.2% to 78%<br>$Pr(I^2 < 5\%) = 0.392$<br>$N = 90$ |
| **Subjective** | $t(-1.57, 1.38^2, 5)$<br>Median = 17%<br>IQR = 9% to 31%<br>95% range = 1% to 74%<br>$Pr(I^2 < 5\%) = 0.120$<br>$N = 154$ | $t(-2.16, 1.40^2, 5)$<br>Median = 10%<br>IQR = 5% to 21%<br>95% range = 0.6% to 65%<br>$Pr(I^2 < 5\%) = 0.254$<br>$N = 123$ | $t(-1.48, 1.02^2, 5)$<br>Median = 19%<br>IQR = 11% to 29%<br>95% range = 3% to 63%<br>$Pr(I^2 < 5\%) = 0.066$<br>$N = 140$ |

**Mean study size between 50 and 200 participants**

| | Pharmacological Vs. Placebo/ Control | Pharmacological Vs. Pharmacological | Non-Pharmacological (Any) |
|---|---|---|---|
| **All-cause mortality** | $t(-3.45, 1.55^2, 5)$<br>Median = 3%<br>IQR = 1% to 7%<br>95% range = 0.1% to 37%<br>$Pr(I^2 < 5\%) = 0.640$<br>$N = 99$ | $t(-4.08, 0.73^2, 5)$<br>Median = 2%<br>IQR = 1% to 3%<br>95% range = 0.3% to 6%<br>$Pr(I^2 < 5\%) = 0.958$<br>$N = 55$ | $t(-3.39, 1.84^2, 5)$<br>Median = 4%<br>IQR = 1% to 9%<br>95% range = 0.05% to 52%<br>$Pr(I^2 < 5\%) = 0.595$<br>$N = 89$ |
| **Semi-objective** | $t(-1.82, 1.54^2, 5)$<br>Median = 14%<br>IQR = 7% to 27%<br>95% range = 0.8% to 77%<br>$Pr(I^2 < 5\%) = 0.186$<br>$N = 156$ | $t(-2.40, 1.63^2, 5)$<br>Median = 8%<br>IQR = 4% to 18%<br>95% range = 0.3% to 69%<br>$Pr(I^2 < 5\%) = 0.336$<br>$N = 152$ | $t(-1.72, 1.85^2, 5)$<br>Median = 15%<br>IQR = 6% to 34%<br>95% range = 0.5% to 89%<br>$Pr(I^2 < 5\%) = 0.217$<br>$N = 269$ |
| **Subjective** | $t(-0.77, 1.37^2, 5)$<br>Median = 32%<br>IQR = 18% to 49%<br>95% range = 3% to 87%<br>$Pr(I^2 < 5\%) = 0.046$<br>$N = 480$ | $t(-1.36, 1.40^2, 5)$<br>Median = 21%<br>IQR = 10% to 37%<br>95% range = 1% to 81%<br>$Pr(I^2 < 5\%) = 0.108$<br>$N = 354$ | $t(-0.68, 1.01, 5)$<br>Median = 34%<br>IQR = 23% to 47%<br>95% range = 6% to 79%<br>$Pr(I^2 < 5\%) = 0.019$<br>$N = 437$ |

**Mean study size > 200 participants**

| | Pharmacological Vs. Placebo/ Control | Pharmacological Vs. Pharmacological | Non-Pharmacological (Any) |
|---|---|---|---|
| **All-cause mortality** | $t(-3.06, 1.55^2, 5)$<br>Median = 5%<br>IQR = 2% to 10%<br>95% range = 0.2% to 45%<br>$Pr(I^2 < 5\%) = 0.506$<br>$N = 78$ | $t(-3.69, 0.74^2, 5)$<br>Median = 3%<br>IQR = 2% to 4%<br>95% range = 0.5% to 8%<br>$Pr(I^2 < 5\%) = 0.871$<br>$N = 37$ | $t(-3.00, 1.84^2, 5)$<br>Median = 5%<br>IQR = 2% to 12%<br>95% range = 0.07% to 61%<br>$Pr(I^2 < 5\%) = 0.479$<br>$N = 65$ |
| **Semi-objective** | $t(-1.43, 1.55^2, 5)$<br>Median = 20%<br>IQR = 9% to 36%<br>95% range = 1% to 83%<br>$Pr(I^2 < 5\%) = 0.124$<br>$N = 81$ | $t(-2.01, 1.63^2, 5)$<br>Median = 12%<br>IQR = 5% to 25%<br>95% range = 0.5% to 77%<br>$Pr(I^2 < 5\%) = 0.242$<br>$N = 102$ | $t(-1.32, 1.85^2, 5)$<br>Median = 21%<br>IQR = 8% to 43%<br>95% range = 0.7% to 92%<br>$Pr(I^2 < 5\%) = 0.159$<br>$N = 129$ |
| **Subjective** | $t(-0.38, 1.38^2, 5)$<br>Median = 41%<br>IQR = 25% to 59%<br>95% range = 4% to 91%<br>$Pr(I^2 < 5\%) = 0.030$<br>$N = 237$ | $t(-0.97, 1.40^2, 5)$<br>Median = 28%<br>IQR = 15% to 46%<br>95% range = 2% to 86%<br>$Pr(I^2 < 5\%) = 0.070$<br>$N = 145$ | $t(-0.29, 1.02^2, 5)$<br>Median = 44%<br>IQR = 30% to 57%<br>95% range = 8% to 85%<br>$Pr(I^2 < 5\%) = 0.010$<br>$N = 217$ |

Table A4: Binary outcome data: Predictive distributions for logit($I^2$) expected in future meta-analyses of log relative risks, together with summary statistics for $I^2$ on the untransformed scale. A $t(\mu, \sigma^2, 5)$ distribution represents a $t$-distribution with location $\mu$, scale $\sigma$ and 5 degrees of freedom. $N$ denotes the total number of meta-analyses contributing in each category.

**Mean study size < 50 participants**

| | Pharmacological Vs. Placebo/ Control | Pharmacological Vs. Pharmacological | Non-Pharmacological (Any) |
|---|---|---|---|
| **All-cause mortality** | $t(-2.26, 0.99^2, 5)$<br>Median = 10%<br>IQR = 6% to 15%<br>95% range = 1% to 37%<br>$Pr(I^2 < 5\%) = 0.178$<br>$N = 39$ | $t(-2.78, 0.74^2, 5)$<br>Median = 6%<br>IQR = 4% to 8%<br>95% range = 1% to 20%<br>$Pr(I^2 < 5\%) = 0.363$<br>$N = 17$ | $t(-2.34, 1.75^2, 5)$<br>Median = 9%<br>IQR = 4% to 20%<br>95% range = 0.3% to 76%<br>$Pr(I^2 < 5\%) = 0.322$<br>$N = 29$ |
| **Semi-objective** | $t(-1.02, 1.32^2, 5)$<br>Median = 27%<br>IQR = 15% to 43%<br>95% range = 2% to 84%<br>$Pr(I^2 < 5\%) = 0.062$<br>$N = 70$ | $t(-1.57, 1.15^2, 5)$<br>Median = 18%<br>IQR = 10% to 29%<br>95% range = 2% to 67%<br>$Pr(I^2 < 5\%) = 0.094$<br>$N = 29$ | $t(-1.16, 2.27^2, 5)$<br>Median = 24%<br>IQR = 8% to 53%<br>95% range = 0.3% to 97%<br>$Pr(I^2 < 5\%) = 0.178$<br>$N = 90$ |
| **Subjective** | $t(-0.30, 1.08^2, 5)$<br>Median = 43%<br>IQR = 29% to 57%<br>95% range = 9% to 87%<br>$Pr(I^2 < 5\%) = 0.010$<br>$N = 154$ | $t(-0.78, 1.30^2, 5)$<br>Median = 32%<br>IQR = 18% to 49%<br>95% range = 4% to 85%<br>$Pr(I^2 < 5\%) = 0.040$<br>$N = 123$ | $t(-0.43, 1.21^2, 5)$<br>Median = 40%<br>IQR = 25% to 57%<br>95% range = 6% to 87%<br>$Pr(I^2 < 5\%) = 0.020$<br>$N = 140$ |

**Mean study size between 50 and 200 participants**

| | Pharmacological Vs. Placebo/ Control | Pharmacological Vs. Pharmacological | Non-Pharmacological (Any) |
|---|---|---|---|
| **All-cause mortality** | $t(-1.87, 0.98^2, 5)$<br>Median = 14%<br>IQR = 9% to 21%<br>95% range = 2% to 47%<br>$Pr(I^2 < 5\%) = 0.103$<br>$N = 99$ | $t(-2.38, 0.72^2, 5)$<br>Median = 9%<br>IQR = 6% to 12%<br>95% range = 2% to 28%<br>$Pr(I^2 < 5\%) = 0.159$<br>$N = 55$ | $t(-1.94, 1.75^2, 5)$<br>Median = 13%<br>IQR = 6% to 12%<br>95% range = 0.4% to 82%<br>$Pr(I^2 < 5\%) = 0.233$<br>$N = 89$ |
| **Semi-objective** | $t(-0.62, 1.31^2, 5)$<br>Median = 35%<br>IQR = 20% to 53%<br>95% range = 4% to 89%<br>$Pr(I^2 < 5\%) = 0.036$<br>$N = 156$ | $t(-1.17, 1.15^2, 5)$<br>Median = 24%<br>IQR = 14% to 38%<br>95% range = 3% to 75%<br>$Pr(I^2 < 5\%) = 0.057$<br>$N = 152$ | $t(-0.77, 2.27^2, 5)$<br>Median = 32%<br>IQR = 12% to 63%<br>95% range = 0.4% to 98%<br>$Pr(I^2 < 5\%) = 0.134$<br>$N = 269$ |
| **Subjective** | $t(\ 0.09, 1.08^2, 5)$<br>Median = 52%<br>IQR = 37% to 67%<br>95% range = 12% to 91%<br>$Pr(I^2 < 5\%) = 0.006$<br>$N = 480$ | $t(-0.39, 1.29^2, 5)$<br>Median = 40%<br>IQR = 24% to 58%<br>95% range = 5% to 89%<br>$Pr(I^2 < 5\%) = 0.022$<br>$N = 354$ | $t(-0.03, 1.20^2, 5)$<br>Median = 49%<br>IQR = 33% to 66%<br>95% range = 8% to 91%<br>$Pr(I^2 < 5\%) = 0.012$<br>$N = 437$ |

**Mean study size > 200 participants**

| | Pharmacological Vs. Placebo/ Control | Pharmacological Vs. Pharmacological | Non-Pharmacological (Any) |
|---|---|---|---|
| **All-cause mortality** | $t(-1.69, 0.98^2, 5)$<br>Median = 16%<br>IQR = 11% to 24%<br>95% range = 2% to 51%<br>$Pr(I^2 < 5\%) = 0.086$<br>$N = 78$ | $t(-2.21, 0.73^2, 5)$<br>Median = 10%<br>IQR = 7% to 14%<br>95% range = 3% to 31%<br>$Pr(I^2 < 5\%) = 0.105$<br>$N = 37$ | $t(-1.77, 1.75^2, 5)$<br>Median = 15%<br>IQR = 6% to 30%<br>95% range = 0.5% to 85%<br>$Pr(I^2 < 5\%) = 0.205$<br>$N = 65$ |
| **Semi-objective** | $t(-0.44, 1.32^2, 5)$<br>Median = 39%<br>IQR = 23% to 57%<br>95% range = 4% to 91%<br>$Pr(I^2 < 5\%) = 0.031$<br>$N = 81$ | $t(-0.99, 1.16^2, 5)$<br>Median = 27%<br>IQR = 16% to 42%<br>95% range = 3% to 79%<br>$Pr(I^2 < 5\%) = 0.043$<br>$N = 102$ | $t(-0.59, 2.27^2, 5)$<br>Median = 36%<br>IQR = 14% to 67%<br>95% range = 0.5% to 98%<br>$Pr(I^2 < 5\%) = 0.119$<br>$N = 129$ |
| **Subjective** | $t(\ 0.27, 1.08^2, 5)$<br>Median = 57%<br>IQR = 41% to 71%<br>95% range = 14% to 92%<br>$Pr(I^2 < 5\%) = 0.056$<br>$N = 237$ | $t(-0.21, 1.29^2, 5)$<br>Median = 45%<br>IQR = 28% to 62%<br>95% range = 6% to 91%<br>$Pr(I^2 < 5\%) = 0.018$<br>$N = 145$ | $t(\ 0.15, 1.21^2, 5)$<br>Median = 54%<br>IQR = 37% to 70%<br>95% range = 10% to 92%<br>$Pr(I^2 < 5\%) = 0.010$<br>$N = 217$ |

Table A5: Binary outcome data: Predictive distributions for logit($I^2$) expected in future meta-analyses of risk differences, together with summary statistics for $I^2$ on the untransformed scale. A $t(\mu, \sigma^2, 5)$ distribution represents a $t$-distribution with location $\mu$, scale $\sigma$ and 5 degrees of freedom. $N$ denotes the total number of meta-analyses contributing in each category.

| | Mean study size < 50 participants | | | Mean study size ≥ 50 participants | | |
|---|---|---|---|---|---|---|
| | **Pharmacological Vs. Placebo/ Control** | **Pharmacological Vs. Pharmacological** | **Non-Pharmacological (Any)** | **Pharmacological Vs. Placebo/ Control** | **Pharmacological Vs. Pharmacological** | **Non-Pharmacological (Any)** |
| **Obstetric outcome** | $t(-2.87, 1.91^2, 5)$<br>Median = 5%<br>IQR = 2% to 15%<br>95% range = 0.1% to 71%<br>$Pr(I^2 < 5\%) = 0.483$<br>$N = 0$ | $t(-3.06, 1.84^2, 5)$<br>Median = 4%<br>IQR = 2% to 12%<br>95% range = 0.1% to 67%<br>$Pr(I^2 < 5\%) = 0.529$<br>$N = 0$ | $t(-2.74, 1.89^2, 5)$<br>Median = 6%<br>IQR = 2% to 16%<br>95% range = 1% to 74%<br>$Pr(I^2 < 5\%) = 0.450$<br>$N = 0$ | $t(-1.90, 1.91^2, 5)$<br>Median = 13%<br>IQR = 4% to 32%<br>95% range = 0.3% to 86%<br>$Pr(I^2 < 5\%) = 0.274$<br>$N = 0$ | $t(-2.09, 1.84^2, 5)$<br>Median = 11%<br>IQR = 4% to 27%<br>95% range = 0.3% to 84%<br>$Pr(I^2 < 5\%) = 0.293$<br>$N = 0$ | $t(-1.76, 1.88^2, 5)$<br>Median = 15%<br>IQR = 5% to 34%<br>95% range = 0.4% to 88%<br>$Pr(I^2 < 5\%) = 0.237$<br>$N = 0$ |
| **Resource use & hospital stay/process** | $t(-1.82, 2.25^2, 5)$<br>Median = 14%<br>IQR = 4% to 38%<br>95% range = 0.2% to 93%<br>$Pr(I^2 < 5\%) = 0.287$<br>$N = 11$ | $t(-2.01, 2.19^2, 5)$<br>Median = 12%<br>IQR = 4% to 32%<br>95% range = 0.2% to 92%<br>$Pr(I^2 < 5\%) = 0.310$<br>$N = 1$ | $t(-1.68, 2.28^2, 5)$<br>Median = 15%<br>IQR = 5% to 41%<br>95% range = 0.2% to 94%<br>$Pr(I^2 < 5\%) = 0.262$<br>$N = 14$ | $t(-0.84, 2.24^2, 5)$<br>Median = 30%<br>IQR = 10% to 62%<br>95% range = 0.5% to 98%<br>$Pr(I^2 < 5\%) = 0.154$<br>$N = 13$ | $t(-1.03, 2.19^2, 5)$<br>Median = 27%<br>IQR = 9% to 55%<br>95% range = 0.4% to 97%<br>$Pr(I^2 < 5\%) = 0.166$<br>$N = 6$ | $t(-0.71, 2.28^2, 5)$<br>Median = 32%<br>IQR = 11% to 65%<br>95% range = 0.5% to 98%<br>$Pr(I^2 < 5\%) = 0.138$<br>$N = 24$ |
| **Internal & External structure related outcome** | $t(-1.74, 2.20^2, 5)$<br>Median = 15%<br>IQR = 5% to 41%<br>95% range = 0.2% to 94%<br>$Pr(I^2 < 5\%) = 0.267$<br>$N = 0$ | $t(-1.93, 2.19^2, 5)$<br>Median = 13%<br>IQR = 4% to 35%<br>95% range = 0.2% to 92%<br>$Pr(I^2 < 5\%) = 0.290$<br>$N = 0$ | $t(-1.60, 2.25^2, 5)$<br>Median = 17%<br>IQR = 5% to 44%<br>95% range = 0.2% to 95%<br>$Pr(I^2 < 5\%) = 0.254$<br>$N = 2$ | $t(-0.76, 2.20^2, 5)$<br>Median = 32%<br>IQR = 11% to 64%<br>95% range = 0.5% to 98%<br>$Pr(I^2 < 5\%) = 0.137$<br>$N = 1$ | $t(-0.95, 2.19^2, 5)$<br>Median = 29%<br>IQR = 10% to 59%<br>95% range = 0.5% to 97%<br>$Pr(I^2 < 5\%) = 0.162$<br>$N = 0$ | $t(-0.63, 2.25^2, 5)$<br>Median = 35%<br>IQR = 12% to 68%<br>95% range = 0.6% to 98%<br>$Pr(I^2 < 5\%) = 0.130$<br>$N = 0$ |
| **General physical health & Adverse event & Pain & Quality of life/functioning** | $t(-2.44, 2.01^2, 5)$<br>Median = 8%<br>IQR = 3% to 23%<br>95% range = 0.1% to 83%<br>$Pr(I^2 < 5\%) = 0.383$<br>$N = 86$ | $t(-2.63, 1.99^2, 5)$<br>Median = 7%<br>IQR = 2% to 19%<br>95% range = 0.1% to 79%<br>$Pr(I^2 < 5\%) = 0.424$<br>$N = 52$ | $t(-2.30, 2.06^2, 5)$<br>Median = 9%<br>IQR = 3% to 25%<br>95% range = 0.2% to 86%<br>$Pr(I^2 < 5\%) = 0.362$<br>$N = 100$ | $t(-1.46, 2.01^2, 5)$<br>Median = 19%<br>IQR = 6% to 44%<br>95% range = 0.4% to 92%<br>$Pr(I^2 < 5\%) = 0.212$<br>$N = 144$ | $t(-1.65, 1.99^2, 5)$<br>Median = 16%<br>IQR = 5% to 39%<br>95% range = 0.4% to 91%<br>$Pr(I^2 < 5\%) = 0.233$<br>$N = 182$ | $t(-1.33, 2.05^2, 5)$<br>Median = 21%<br>IQR = 7% to 47%<br>95% range = 0.4% to 94%<br>$Pr(I^2 < 5\%) = 0.190$<br>$N = 70$ |
| **Signs/symptoms reflecting continuation/end of condition & Infection/onset of new acute/chronic disease** | $t(-2.36, 2.11^2, 5)$<br>Median = 9%<br>IQR = 3% to 25%<br>95% range = 0.1% to 86%<br>$Pr(I^2 < 5\%) = 0.379$<br>$N = 13$ | $t(-2.55, 2.11^2, 5)$<br>Median = 7%<br>IQR = 2% to 21%<br>95% range = 0.1% to 84%<br>$Pr(I^2 < 5\%) = 0.410$<br>$N = 7$ | $t(-2.23, 2.13^2, 5)$<br>Median = 10%<br>IQR = 3% to 27%<br>95% range = 0.2% to 89%<br>$Pr(I^2 < 5\%) = 0.339$<br>$N = 12$ | $t(-1.39, 2.11^2, 5)$<br>Median = 20%<br>IQR = 7% to 47%<br>95% range = 0.3% to 94%<br>$Pr(I^2 < 5\%) = 0.204$<br>$N = 80$ | $t(-1.58, 2.11^2, 5)$<br>Median = 17%<br>IQR = 6% to 42%<br>95% range = 0.3% to 94%<br>$Pr(I^2 < 5\%) = 0.231$<br>$N = 100$ | $t(-1.25, 2.13^2, 5)$<br>Median = 22%<br>IQR = 8% to 49%<br>95% range = 0.3% to 96%<br>$Pr(I^2 < 5\%) = 0.182$<br>$N = 19$ |
| **Mental health outcome** | $t(-2.11, 1.76^2, 5)$<br>Median = 11%<br>IQR = 4% to 25%<br>95% range = 0.3% to 81%<br>$Pr(I^2 < 5\%) = 0.288$<br>$N = 0$ | $t(-2.30, 1.71^2, 5)$<br>Median = 9%<br>IQR = 4% to 22%<br>95% range = 0.3% to 75%<br>$Pr(I^2 < 5\%) = 0.331$<br>$N = 0$ | $t(-1.98, 1.77^2, 5)$<br>Median = 12%<br>IQR = 5% to 28%<br>95% range = 0.4% to 83%<br>$Pr(I^2 < 5\%) = 0.261$<br>$N = 1$ | $t(-1.14, 1.76^2, 5)$<br>Median = 24%<br>IQR = 11% to 47%<br>95% range = 0.9% to 92%<br>$Pr(I^2 < 5\%) = 0.130$<br>$N = 0$ | $t(-1.33, 1.71^2, 5)$<br>Median = 21%<br>IQR = 9% to 42%<br>95% range = 0.8% to 89%<br>$Pr(I^2 < 5\%) = 0.142$<br>$N = 0$ | $t(-1.00, 1.76^2, 5)$<br>Median = 27%<br>IQR = 12% to 50%<br>95% range = 1% to 93%<br>$Pr(I^2 < 5\%) = 0.112$<br>$N = 3$ |
| **Biological-marker** | $t(-2.81, 2.48^2, 5)$<br>Median = 6%<br>IQR = 1% to 21%<br>95% range = 0.04% to 90%<br>$Pr(I^2 < 5\%) = 0.477$<br>$N = 11$ | $t(-3.00, 2.42^2, 5)$<br>Median = 5%<br>IQR = 1% to 16%<br>95% range = 0.04% to 87%<br>$Pr(I^2 < 5\%) = 0.515$<br>$N = 4$ | $t(-2.68, 2.50^2, 5)$<br>Median = 6%<br>IQR = 2% to 23%<br>95% range = 0.05% to 91%<br>$Pr(I^2 < 5\%) = 0.455$<br>$N = 15$ | $t(-1.84, 2.48^2, 5)$<br>Median = 14%<br>IQR = 3% to 42%<br>95% range = 0.1% to 96%<br>$Pr(I^2 < 5\%) = 0.312$<br>$N = 11$ | $t(-2.03, 2.42^2, 5)$<br>Median = 12%<br>IQR = 3% to 34%<br>95% range = 0.09% to 94%<br>$Pr(I^2 < 5\%) = 0.327$<br>$N = 10$ | $t(-1.70, 2.49^2, 5)$<br>Median = 15%<br>IQR = 4% to 44%<br>95% range = 0.1% to 96%<br>$Pr(I^2 < 5\%) = 0.286$<br>$N = 6$ |
| **Various subjectively measured outcomes** | $t(-1.89, 2.17^2, 5)$<br>Median = 13%<br>IQR = 4% to 35%<br>95% range = 0.2% to 92%<br>$Pr(I^2 < 5\%) = 0.292$<br>$N = 9$ | $t(-2.08, 2.10^2, 5)$<br>Median = 11%<br>IQR = 4% to 30%<br>95% range = 0.2% to 87%<br>$Pr(I^2 < 5\%) = 0.313$<br>$N = 2$ | $t(-1.76, 2.17^2, 5)$<br>Median = 15%<br>IQR = 5% to 38%<br>95% range = 0.2% to 92%<br>$Pr(I^2 < 5\%) = 0.262$<br>$N = 3$ | $t(-0.92, 2.17^2, 5)$<br>Median = 29%<br>IQR = 10% to 59%<br>95% range = 0.6% to 97%<br>$Pr(I^2 < 5\%) = 0.148$<br>$N = 21$ | $t(-1.11, 2.10^2, 5)$<br>Median = 26%<br>IQR = 9% to 54%<br>95% range = 0.5% to 95%<br>$Pr(I^2 < 5\%) = 0.163$<br>$N = 13$ | $t(-0.78, 2.17^2, 5)$<br>Median = 32%<br>IQR = 11% to 62%<br>95% range = 0.6% to 97%<br>$Pr(I^2 < 5\%) = 0.131$<br>$N = 4$ |

Table A6: Continuous outcome data: Predictive distributions for logit($I^2$) in future meta-analyses specializing in respiratory diseases, performed on the standardized mean difference scale. Summary statistics are for $I^2$ on the untransformed scale. A $t(\mu, \sigma^2, 5)$ distribution represents a $t$-distribution with location $\mu$, scale $\sigma$ and 5 degrees of freedom. $N$ denotes the total number of meta-analyses contributing in each category.

| | Mean study size < 50 participants | | | Mean study size ≥ 50 participants | | |
|---|---|---|---|---|---|---|
| | **Pharmacological Vs. Placebo/ Control** | **Pharmacological Vs. Pharmacological** | **Non-Pharmacological (Any)** | **Pharmacological Vs. Placebo/ Control** | **Pharmacological Vs. Pharmacological** | **Non-Pharmacological (Any)** |
| **Obstetric outcome** | $t\ (0.44, 2.02^2, 5)$<br>Median = 60%<br>IQR = 31% to 84%<br>95% range = 3% to 99%<br>$Pr(I^2 < 5\%) = 0.048$<br>$N = 3$ | $t(\ 0.25, 1.95^2, 5)$<br>Median = 56%<br>IQR = 28% to 81%<br>95% range = 2% to 99%<br>$Pr(I^2 < 5\%) = 0.050$<br>$N = 7$ | $t(\ 0.57, 2.00^2, 5)$<br>Median = 64%<br>IQR = 35% to 86%<br>95% range = 3% to 99%<br>$Pr(I^2 < 5\%) = 0.042$<br>$N = 8$ | $t(\ 1.41, 2.01^2, 5)$<br>Median = 80%<br>IQR = 54% to 93%<br>95% range = 7% to 99.6%<br>$Pr(I^2 < 5\%) = 0.018$<br>$N = 0$ | $t(\ 1.22, 1.95^2, 5)$<br>Median = 77%<br>IQR = 51% to 91%<br>95% range = 6% to 99%<br>$Pr(I^2 < 5\%) = 0.020$<br>$N = 0$ | $t(\ 1.55, 1.99^2, 5)$<br>Median = 83%<br>IQR = 59% to 94%<br>95% range = 7% to 99.6%<br>$Pr(I^2 < 5\%) = 0.016$<br>$N = 0$ |
| **Resource use &<br>hospital stay/process** | $t(\ 1.49, 2.33^2, 5)$<br>Median = 82%<br>IQR = 51% to 95%<br>95% range = 4% to 99.8%<br>$Pr(I^2 < 5\%) = 0.031$<br>$N = 1$ | $t(\ 1.30, 2.28^2, 5)$<br>Median = 79%<br>IQR = 48% to 94%<br>95% range = 4% to 99.7%<br>$Pr(I^2 < 5\%) = 0.030$<br>$N = 0$ | $t(1.63, 2.36^2, 5)$<br>Median = 84%<br>IQR = 55% to 95%<br>95% range = 5% to 99.8%<br>$Pr(I^2 < 5\%) = 0.026$<br>$N = 0$ | $t(\ 2.47, 2.33^2, 5)$<br>Median = 92%<br>IQR = 74% to 98%<br>95% range = 10% to 99.9%<br>$Pr(I^2 < 5\%) = 0.014$<br>$N = 1$ | $t(\ 2.28, 2.27^2, 5)$<br>Median = 91%<br>IQR = 71% to 97%<br>95% range = 10% to 99.9%<br>$Pr(I^2 < 5\%) = 0.015$<br>$N = 0$ | $t(\ 2.60, 2.36^2, 5)$<br>Median = 93%<br>IQR = 77% to 98%<br>95% range = 11% to 99.9%<br>$Pr(I^2 < 5\%) = 0.014$<br>$N = 6$ |
| **Internal &<br>External structure<br>related outcome** | $t(\ 1.57, 2.31^2, 5)$<br>Median = 83%<br>IQR = 55% to 95%<br>95% range = 4% to 99.8%<br>$Pr(I^2 < 5\%) = 0.030$<br>$N = 0$ | $t(\ 1.38, 2.30^2, 5)$<br>Median = 81%<br>IQR = 50% to 94%<br>95% range = 4% to 99.7%<br>$Pr(I^2 < 5\%) = 0.031$<br>$N = 0$ | $t(\ 1.71, 2.35^2, 5)$<br>Median = 85%<br>IQR = 56% to 96%<br>95% range = 4% to 99.8%<br>$Pr(I^2 < 5\%) = 0.027$<br>$N = 0$ | $t(\ 2.55, 2.31^2, 5)$<br>Median = 93%<br>IQR = 76% to 98%<br>95% range = 10% to 99.9%<br>$Pr(I^2 < 5\%) = 0.014$<br>$N = 0$ | $t(\ 2.36, 2.29^2, 5)$<br>Median = 92%<br>IQR = 72% to 98%<br>95% range = 9% to 99.9%<br>$Pr(I^2 < 5\%) = 0.016$<br>$N = 0$ | $t(\ 2.68, 2.35^2, 5)$<br>Median = 94%<br>IQR = 77% to 98%<br>95% range = 11% to 99.9%<br>$Pr(I^2 < 5\%) = 0.013$<br>$N = 0$ |
| **General physical health &<br>Adverse event &<br>Pain &<br>Quality of life/functioning** | $t(0.87, 2.11^2, 5)$<br>Median = 70%<br>IQR = 40% to 90%<br>95% range = 4% to 99%<br>$Pr(I^2 < 5\%) = 0.037$<br>$N = 0$ | $t(0.68, 2.09^2, 5)$<br>Median = 66%<br>IQR = 35% to 87%<br>95% range = 3% to 99%<br>$Pr(I^2 < 5\%) = 0.040$<br>$N = 0$ | $t(\ 1.01, 2.15^2, 5)$<br>Median = 73%<br>IQR = 43% to 91%<br>95% range = 3% to 99%<br>$Pr(I^2 < 5\%) = 0.034$<br>$N = 2$ | $t(\ 1.85, 2.10^2, 5)$<br>Median = 86%<br>IQR = 63% to 96%<br>95% range = 8% to 99.8%<br>$Pr(I^2 < 5\%) = 0.014$<br>$N = 1$ | $t(\ 1.66, 2.08^2, 5)$<br>Median = 84%<br>IQR = 59% to 95%<br>95% range = 8% to 99.7%<br>$Pr(I^2 < 5\%) = 0.017$<br>$N = 0$ | $t(\ 1.98, 2.14^2, 5)$<br>Median = 88%<br>IQR = 67% to 96%<br>95% range = 9% to 99.8%<br>$Pr(I^2 < 5\%) = 0.015$<br>$N = 2$ |
| **Signs/symptoms reflecting<br>continuation/end of condition<br>& Infection/onset of new<br>acute/chronic disease** | $t(\ 0.95, 2.21^2, 5)$<br>Median = 72%<br>IQR = 41% to 91%<br>95% range = 3% to 99.6%<br>$Pr(I^2 < 5\%) = 0.038$<br>$N = 0$ | $t(0.76, 2.20^2, 5)$<br>Median = 68%<br>IQR = 36% to 89%<br>95% range = 2% to 99%<br>$Pr(I^2 < 5\%) = 0.044$<br>$N = 0$ | $t(\ 1.08, 2.23^2, 5)$<br>Median = 75%<br>IQR = 45% to 92%<br>95% range = 3% to 99.6%<br>$Pr(I^2 < 5\%) = 0.038$<br>$N = 0$ | $t(1.92, 2.20^2, 5)$<br>Median = 87%<br>IQR = 65% to 96%<br>95% range = 8% to 99.8%<br>$Pr(I^2 < 5\%) = 0.017$<br>$N = 0$ | $t(\ 1.73, 2.20^2, 5)$<br>Median = 85%<br>IQR = 60% to 96%<br>95% range = 6% to 99.8%<br>$Pr(I^2 < 5\%) = 0.020$<br>$N = 0$ | $t(\ 2.06, 2.22^2, 5)$<br>Median = 89%<br>IQR = 69% to 97%<br>95% range = 7% to 99.8%<br>$Pr(I^2 < 5\%) = 0.020$<br>$N = 1$ |
| **Mental health outcome** | $t(\ 1.20, 1.87^2, 5)$<br>Median = 77%<br>IQR = 52% to 91%<br>95% range = 7% to 99%<br>$Pr(I^2 < 5\%) = 0.019$<br>$N = 0$ | $t(\ 1.01, 1.83^2, 5)$<br>Median = 73%<br>IQR = 48% to 89%<br>95% range = 7% to 99%<br>$Pr(I^2 < 5\%) = 0.019$<br>$N = 0$ | $t(\ 1.33, 1.88^2, 5)$<br>Median = 79%<br>IQR = 55% to 92%<br>95% range = 8% to 99%<br>$Pr(I^2 < 5\%) = 0.017$<br>$N = 0$ | $t(\ 2.17, 1.87^2, 5)$<br>Median = 90%<br>IQR = 74% to 96%<br>95% range = 16% to 99.8%<br>$Pr(I^2 < 5\%) = 0.008$<br>$N = 1$ | $t(\ 1.98, 1.82^2, 5)$<br>Median = 88%<br>IQR = 71% to 96%<br>95% range = 17% to 99.6%<br>$Pr(I^2 < 5\%) = 0.008$<br>$N = 1$ | $t(-2.31, 1.87^2, 5)$<br>Median = 91%<br>IQR = 77% to 97%<br>95% range = 18% to 99.8%<br>$Pr(I^2 < 5\%) = 0.007$<br>$N = 3$ |
| **Biological-marker** | $t(\ 0.50, 2.56^2, 5)$<br>Median = 62%<br>IQR = 25% to 89%<br>95% range = 1% to 99.7%<br>$Pr(I^2 < 5\%) = 0.080$<br>$N = 1$ | $t(\ 0.31, 2.50^2, 5)$<br>Median = 57%<br>IQR = 24% to 85%<br>95% range = 0.8% to 99.6%<br>$Pr(I^2 < 5\%) = 0.079$<br>$N = 0$ | $t(\ 0.63, 2.57^2, 5)$<br>Median = 66%<br>IQR = 28% to 90%<br>95% range = 1% to 99.7%<br>$Pr(I^2 < 5\%) = 0.072$<br>$N = 0$ | $t(\ 1.48, 2.56^2, 5)$<br>Median = 81%<br>IQR = 47% to 95%<br>95% range = 2% to 99.9%<br>$Pr(I^2 < 5\%) = 0.040$<br>$N = 0$ | $t(\ 1.28, 2.50^2, 5)$<br>Median = 78%<br>IQR = 45% to 94%<br>95% range = 2% to 99.8%<br>$Pr(I^2 < 5\%) = 0.043$<br>$N = 0$ | $t(\ 1.61, 2.57^2, 5)$<br>Median = 84%<br>IQR = 51% to 96%<br>95% range = 2% to 99.9%<br>$Pr(I^2 < 5\%) = 0.036$<br>$N = 0$ |
| **Various subjectively<br>measured outcomes** | $t(1.42, 2.26^2, 5)$<br>Median = 81%<br>IQR = 51% to 94%<br>95% range = 5% to 99.7%<br>$Pr(I^2 < 5\%) = 0.026$<br>$N = 0$ | $t(\ 1.23, 2.19^2, 5)$<br>Median = 78%<br>IQR = 48% to 93%<br>95% range = 4% to 99.6%<br>$Pr(I^2 < 5\%) = 0.029$<br>$N = 0$ | $t(\ 1.55, 2.27^2, 5)$<br>Median = 83%<br>IQR = 55% to 95%<br>95% range = 5% to 99.7%<br>$Pr(I^2 < 5\%) = 0.027$<br>$N = 0$ | $t(\ 2.40, 2.26^2, 5)$<br>Median = 92%<br>IQR = 73% to 98%<br>95% range = 12% to 99.9%<br>$Pr(I^2 < 5\%) = 0.010$<br>$N = 0$ | $t(\ 2.20, 2.19^2, 5)$<br>Median = 90%<br>IQR = 71% to 97%<br>95% range = 11% to 99.8%<br>$Pr(I^2 < 5\%) = 0.013$<br>$N = 0$ | $t(\ 2.53, 2.26^2, 5)$<br>Median = 93%<br>IQR = 77% to 98%<br>95% range = 11% to 99.9%<br>$Pr(I^2 < 5\%) = 0.014$<br>$N = 0$ |

Table A7: Continuous outcome data: Predictive distributions for $\text{logit}(I^2)$ in future meta-analyses specializing in cancer, performed on the standardized mean difference scale. Summary statistics are for $I^2$ on the untransformed scale. A $t(\mu, \sigma^2, 5)$ distribution represents a $t$-distribution with location $\mu$, scale $\sigma$ and 5 degrees of freedom. $N$ denotes the total number of meta-analyses contributing in each category.

| | **Mean study size < 50 participants** | | | **Mean study size ≥ 50 participants** | | |
|---|---|---|---|---|---|---|
| | **Pharmacological Vs. Placebo/ Control** | **Pharmacological Vs. Pharmacological** | **Non-Pharmacological (Any)** | **Pharmacological Vs. Placebo/ Control** | **Pharmacological Vs. Pharmacological** | **Non-Pharmacological (Any)** |
| **Obstetric outcome** | $t(-0.73, 1.80^2, 5)$<br>Median = 33%<br>IQR = 14% to 58%<br>95% range = 1% to 94%<br>$Pr(I^2 < 5\%) = 0.091$<br>$N = 3$ | $t(-1.28, 1.72^2, 5)$<br>Median = 22%<br>IQR = 9% to 43%<br>95% range = 0.8% to 91%<br>$Pr(I^2 < 5\%) = 0.138$<br>$N = 7$ | $t(-0.77, 1.74^2, 5)$<br>Median = 31%<br>IQR = 14% to 56%<br>95% range = 1% to 94%<br>$Pr(I^2 < 5\%) = 0.085$<br>$N = 8$ | $t(0.02, 1.80^2, 5)$<br>Median = 51%<br>IQR = 26% to 74%<br>95% range = 3% to 97%<br>$Pr(I^2 < 5\%) = 0.045$<br>$N = 47$ | $t(-0.53, 1.72^2, 5)$<br>Median = 37%<br>IQR = 18% to 62%<br>95% range = 2% to 95%<br>$Pr(I^2 < 5\%) = 0.067$<br>$N = 39$ | $t(-0.02, 1.73^2, 5)$<br>Median = 49%<br>IQR = 26% to 73%<br>95% range = 3% to 97%<br>$Pr(I^2 < 5\%) = 0.041$<br>$N = 61$ |
| **Resource use & hospital stay/process** | $t(-0.30, 2.53^2, 5)$<br>Median = 43%<br>IQR = 14% to 77%<br>95% range = 0.4% to 99%<br>$Pr(I^2 < 5\%) = 0.129$<br>$N = 33$ | $t(-0.85, 2.47^2, 5)$<br>Median = 30%<br>IQR = 9% to 64%<br>95% range = 0.2% to 99%<br>$Pr(I^2 < 5\%) = 0.168$<br>$N = 0$ | $t(-0.34, 2.54^2, 5)$<br>Median = 41%<br>IQR = 14% to 76%<br>95% range = 0.5% to 99%<br>$Pr(I^2 < 5\%) = 0.131$<br>$N = 16$ | $t(0.45, 2.53^2, 5)$<br>Median = 61%<br>IQR = 26% to 89%<br>95% range = 0.9% to 99.6%<br>$Pr(I^2 < 5\%) = 0.077$<br>$N = 45$ | $t(-0.10, 2.47^2, 5)$<br>Median = 47%<br>IQR = 17% to 79%<br>95% range = 0.5% to 99%<br>$Pr(I^2 < 5\%) = 0.108$<br>$N = 46$ | $t(0.41, 2.53^2, 5)$<br>Median = 60%<br>IQR = 25% to 87%<br>95% range = 1% to 99.6%<br>$Pr(I^2 < 5\%) = 0.078$<br>$N = 200$ |
| **Internal & External structure related outcome** | $t(0.17, 2.05^2, 5)$<br>Median = 54%<br>IQR = 26% to 80%<br>95% range = 2% to 99%<br>$Pr(I^2 < 5\%) = 0.057$<br>$N = 32$ | $t(-0.38, 2.03^2, 5)$<br>Median = 41%<br>IQR = 17% to 69%<br>95% range = 1% to 97%<br>$Pr(I^2 < 5\%) = 0.089$<br>$N = 3$ | $t(-13, 2.05^2, 5)$<br>Median = 54%<br>IQR = 25% to 79%<br>95% range = 2% to 99%<br>$Pr(I^2 < 5\%) = 0.060$<br>$N = 9$ | $t(0.92, 2.05^2, 5)$<br>Median = 72%<br>IQR = 42% to 90%<br>95% range = 3% to 99%<br>$Pr(I^2 < 5\%) = 0.033$<br>$N = 42$ | $t(0.37, 2.03^2, 5)$<br>Median = 60%<br>IQR = 30% to 82%<br>95% range = 2% to 99%<br>$Pr(I^2 < 5\%) = 0.048$<br>$N = 1$ | $t(0.88, 2.05^2, 5)$<br>Median = 71%<br>IQR = 42% to 89%<br>95% range = 4% to 99%<br>$Pr(I^2 < 5\%) = 0.034$<br>$N = 35$ |
| **General physical health & Adverse event & Pain & Quality of life/functioning** | $t(-0.41, 2.12^2, 5)$<br>Median = 40%<br>IQR = 15% to 71%<br>95% range = 0.8% to 99%<br>$Pr(I^2 < 5\%) = 0.102$<br>$N = 143$ | $t(-0.96, 2.10^2, 5)$<br>Median = 28%<br>IQR = 10% to 58%<br>95% range = 0.5% to 99%<br>$Pr(I^2 < 5\%) = 0.144$<br>$N = 57$ | $t(-0.45, 2.13^2, 5)$<br>Median = 39%<br>IQR = 15% to 69%<br>95% range = 0.9% to 99%<br>$Pr(I^2 < 5\%) = 0.109$<br>$N = 249$ | $t(0.33, 2.12^2, 5)$<br>Median = 58%<br>IQR = 28% to 83%<br>95% range = 2% to 99%<br>$Pr(I^2 < 5\%) = 0.059$<br>$N = 413$ | $t(-0.22, 2.10^2, 5)$<br>Median = 45%<br>IQR = 18% to 74%<br>95% range = 1% to 98%<br>$Pr(I^2 < 5\%) = 0.084$<br>$N = 117$ | $t(0.30, 2.13^2, 5)$<br>Median = 57%<br>IQR = 28% to 82%<br>95% range = 2% to 99%<br>$Pr(I^2 < 5\%) = 0.059$<br>$N = 388$ |
| **Signs/symptoms reflecting continuation/end of condition & Infection/onset of new acute/chronic disease** | $t(-0.15, 2.05^2, 5)$<br>Median = 47%<br>IQR = 20% to 75%<br>95% range = 1% to 98%<br>$Pr(I^2 < 5\%) = 0.076$<br>$N = 63$ | $t(-0.70, 2.04^2, 5)$<br>Median = 33%<br>IQR = 13% to 63%<br>95% range = 8% to 97%<br>$Pr(I^2 < 5\%) = 0.115$<br>$N = 41$ | $t(-0.18, 2.04^2, 5)$<br>Median = 45%<br>IQR = 21% to 73%<br>95% range = 1% to 98%<br>$Pr(I^2 < 5\%) = 0.076$<br>$N = 76$ | $t(0.60, 2.05^2, 5)$<br>Median = 65%<br>IQR = 35% to 86%<br>95% range = 3% to 99%<br>$Pr(I^2 < 5\%) = 0.039$<br>$N = 221$ | $t(0.05, 2.04^2, 5)$<br>Median = 51%<br>IQR = 24% to 78%<br>95% range = 2% to 98%<br>$Pr(I^2 < 5\%) = 0.062$<br>$N = 69$ | $t(0.56, 2.03^2, 5)$<br>Median = 63%<br>IQR = 36% to 85%<br>95% range = 3% to 99%<br>$Pr(I^2 < 5\%) = 0.042$<br>$N = 143$ |
| **Mental health outcome** | $t(-0.25, 1.76^2, 5)$<br>Median = 44%<br>IQR = 22% to 68%<br>95% range = 2% to 97%<br>$Pr(I^2 < 5\%) = 0.057$<br>$N = 39$ | $t(-0.80, 1.69^2, 5)$<br>Median = 31%<br>IQR = 15% to 54%<br>95% range = 1% to 93%<br>$Pr(I^2 < 5\%) = 0.080$<br>$N = 16$ | $t(-0.28, 1.72^2, 5)$<br>Median = 42%<br>IQR = 22% to 67%<br>95% range = 2% to 96%<br>$Pr(I^2 < 5\%) = 0.053$<br>$N = 30$ | $t(0.50, 1.76^2, 5)$<br>Median = 62%<br>IQR = 38% to 82%<br>95% range = 5% to 98%<br>$Pr(I^2 < 5\%) = 0.028$<br>$N = 106$ | $t(-0.05, 1.69^2, 5)$<br>Median = 48%<br>IQR = 27% to 72%<br>95% range = 3% to 96%<br>$Pr(I^2 < 5\%) = 0.040$<br>$N = 46$ | $t(0.46, 1.71^2, 5)$<br>Median = 61%<br>IQR = 37% to 81%<br>95% range = 5% to 98%<br>$Pr(I^2 < 5\%) = 0.027$<br>$N = 64$ |
| **Biological-marker** | $t(-0.58, 2.48^2, 5)$<br>Median = 36%<br>IQR = 11% to 72%<br>95% range = 0.4% to 99%<br>$Pr(I^2 < 5\%) = 0.151$<br>$N = 175$ | $t(-1.13, 2.42^2, 5)$<br>Median = 24%<br>IQR = 7% to 57%<br>95% range = 0.2% to 98%<br>$Pr(I^2 < 5\%) = 0.193$<br>$N = 51$ | $t(-0.61, 2.46^2, 5)$<br>Median = 35%<br>IQR = 11% to 69%<br>95% range = 0.4% to 99%<br>$Pr(I^2 < 5\%) = 0.148$<br>$N = 128$ | $t(0.17, 2.48^2, 5)$<br>Median = 54%<br>IQR = 21% to 84%<br>95% range = 0.9% to 99%<br>$Pr(I^2 < 5\%) = 0.089$<br>$N = 212$ | $t(-0.38, 2.42^2, 5)$<br>Median = 41%<br>IQR = 14% to 73%<br>95% range = 0.5% to 99%<br>$Pr(I^2 < 5\%) = 0.119$<br>$N = 99$ | $t(0.13, 2.46^2, 5)$<br>Median = 53%<br>IQR = 20% to 83%<br>95% range = 0.8% to 99%<br>$Pr(I^2 < 5\%) = 0.085$<br>$N = 268$ |
| **Various subjectively measured outcomes** | $t(0.42, 2.27^2, 5)$<br>Median = 61%<br>IQR = 28% to 86%<br>95% range = 2% to 99%<br>$Pr(I^2 < 5\%) = 0.056$<br>$N = 14$ | $t(-0.13, 2.20^2, 5)$<br>Median = 48%<br>IQR = 20% to 77%<br>95% range = 1% to 98%<br>$Pr(I^2 < 5\%) = 0.081$<br>$N = 4$ | $t(0.39, 2.24^2, 5)$<br>Median = 60%<br>IQR = 29% to 85%<br>95% range = 2% to 99%<br>$Pr(I^2 < 5\%) = 0.059$<br>$N = 31$ | $t(1.17, 2.27^2, 5)$<br>Median = 77%<br>IQR = 45% to 93%<br>95% range = 4% to 99.6%<br>$Pr(I^2 < 5\%) = 0.031$<br>$N = 37$ | $t(0.62, 2.19^2, 5)$<br>Median = 66%<br>IQR = 34% to 87%<br>95% range = 2% to 99%<br>$Pr(I^2 < 5\%) = 0.044$<br>$N = 30$ | $t(1.13, 2.23^2, 5)$<br>Median = 76%<br>IQR = 46% to 92%<br>95% range = 3% to 99.6%<br>$Pr(I^2 < 5\%) = 0.036$<br>$N = 104$ |

Table A8: Continuous outcome data: Predictive distributions for logit($I^2$) in future meta-analyses specializing in therapeutic areas other than cancer and respiratory diseases, performed on the mean difference scale. Summary statistics are for $I^2$ on the untransformed scale. A $t(\mu, \sigma^2, 5)$ distribution represents a $t$-distribution with location $\mu$, scale $\sigma$ and 5 degrees of freedom. $N$ denotes the total number of meta-analyses contributing in each category.

| | Mean study size < 50 participants | | | Mean study size ≥ 50 participants | | |
|---|---|---|---|---|---|---|
| | **Pharmacological Vs. Placebo/ Control** | **Pharmacological Vs. Pharmacological** | **Non-Pharmacological (Any)** | **Pharmacological Vs. Placebo/ Control** | **Pharmacological Vs. Pharmacological** | **Non-Pharmacological (Any)** |
| **Obstetric outcome** | $t(-1.82, 1.81^2, 5)$<br>Median = 14%<br>IQR = 5% to 32%<br>95% range = 0.4% to 85%<br>$Pr(I^2 < 5\%) = 0.243$<br>$N = 0$ | $t(-2.37, 1.73^2, 5)$<br>Median = 9%<br>IQR = 3% to 20%<br>95% range = 0.3% to 78%<br>$Pr(I^2 < 5\%) = 0.343$<br>$N = 0$ | $t(-1.85, 1.75^2, 5)$<br>Median = 13%<br>IQR = 5% to 31%<br>95% range = 0.4% to 85%<br>$Pr(I^2 < 5\%) = 0.240$<br>$N = 0$ | $t(-1.07, 1.81^2, 5)$<br>Median = 25%<br>IQR = 10% to 50%<br>95% range = 0.9% to 92%<br>$Pr(I^2 < 5\%) = 0.128$<br>$N = 0$ | $t(-1.62, 1.73^2, 5)$<br>Median = 17%<br>IQR = 7% to 35%<br>95% range = 0.6% to 87%<br>$Pr(I^2 < 5\%) = 0.186$<br>$N = 0$ | $t(-1.11, 1.75^2, 5)$<br>Median = 25%<br>IQR = 11% to 48%<br>95% range = 0.9% to 92%<br>$Pr(I^2 < 5\%) = 0.120$<br>$N = 0$ |
| **Resource use & hospital stay/process** | $t(-1.39, 2.54^2, 5)$<br>Median = 20%<br>IQR = 5% to 54%<br>95% range = 0.1% to 98%<br>$Pr(I^2 < 5\%) = 0.246$<br>$N = 11$ | $t(-1.94, 2.48^2, 5)$<br>Median = 13%<br>IQR = 3% to 38%<br>95% range = 0.08% to 96%<br>$Pr(I^2 < 5\%) = 0.323$<br>$N = 1$ | $t(-1.42, 2.55^2, 5)$<br>Median = 19%<br>IQR = 5% to 52%<br>95% range = 0.2% to 97%<br>$Pr(I^2 < 5\%) = 0.251$<br>$N = 14$ | $t(-0.64, 2.54^2, 5)$<br>Median = 34%<br>IQR = 10% to 71%<br>95% range = 0.3% to 99%<br>$Pr(I^2 < 5\%) = 0.158$<br>$N = 13$ | $t(-1.19, 2.48^2, 5)$<br>Median = 24%<br>IQR = 7% to 56%<br>95% range = 0.2% to 98%<br>$Pr(I^2 < 5\%) = 0.213$<br>$N = 6$ | $t(-0.67, 2.54^2, 5)$<br>Median = 33%<br>IQR = 10% to 69%<br>95% range = 0.3% to 99%<br>$Pr(I^2 < 5\%) = 0.159$<br>$N = 24$ |
| **Internal & External structure related outcome** | $t(-0.92, 2.05^2, 5)$<br>Median = 29%<br>IQR = 10% to 58%<br>95% range = 0.6% to 96%<br>$Pr(I^2 < 5\%) = 0.137$<br>$N = 0$ | $t(-1.47, 2.03^2, 5)$<br>Median = 19%<br>IQR = 6% to 43%<br>95% range = 0.4% to 93%<br>$Pr(I^2 < 5\%) = 0.209$<br>$N = 0$ | $t(-0.95, 2.06^2, 5)$<br>Median = 28%<br>IQR = 10% to 58%<br>95% range = 0.6% to 96%<br>$Pr(I^2 < 5\%) = 0.144$<br>$N = 2$ | $t(-0.17, 2.05^2, 5)$<br>Median = 46%<br>IQR = 20% to 74%<br>95% range = 1% to 98%<br>$Pr(I^2 < 5\%) = 0.078$<br>$N = 1$ | $t(-0.72, 2.03^2, 5)$<br>Median = 34%<br>IQR = 13% to 61%<br>95% range = 0.8% to 97%<br>$Pr(I^2 < 5\%) = 0.118$<br>$N = 0$ | $t(-0.21, 2.06^2, 5)$<br>Median = 45%<br>IQR = 20% to 74%<br>95% range = 1% to 98%<br>$Pr(I^2 < 5\%) = 0.080$<br>$N = 0$ |
| **General physical health & Adverse event & Pain & Quality of life/functioning** | $t(-1.50, 2.13^2, 5)$<br>Median = 19%<br>IQR = 6% to 45%<br>95% range = 0.3% to 94%<br>$Pr(I^2 < 5\%) = 0.227$<br>$N = 86$ | $t(-2.05, 2.10^2, 5)$<br>Median = 12%<br>IQR = 3% to 31%<br>95% range = 0.2% to 90%<br>$Pr(I^2 < 5\%) = 0.313$<br>$N = 52$ | $t(-1.54, 2.14^2, 5)$<br>Median = 18%<br>IQR = 6% to 43%<br>95% range = 0.3% to 94%<br>$Pr(I^2 < 5\%) = 0.232$<br>$N = 100$ | $t(-0.75, 2.13^2, 5)$<br>Median = 32%<br>IQR = 11% to 63%<br>95% range = 0.6% to 97%<br>$Pr(I^2 < 5\%) = 0.137$<br>$N = 144$ | $t(-1.30, 2.10^2, 5)$<br>Median = 22%<br>IQR = 7% to 49%<br>95% range = 0.4% to 95%<br>$Pr(I^2 < 5\%) = 0.189$<br>$N = 182$ | $t(-0.79, 2.14^2, 5)$<br>Median = 32%<br>IQR = 11% to 62%<br>95% range = 0.7% to 97%<br>$Pr(I^2 < 5\%) = 0.139$<br>$N = 70$ |
| **Signs/symptoms reflecting continuation/end of condition & Infection/onset of new acute/chronic disease** | $t(-1.24, 2.06^2, 5)$<br>Median = 23%<br>IQR = 8% to 50%<br>95% range = 0.5% to 94%<br>$Pr(I^2 < 5\%) = 0.179$<br>$N = 13$ | $t(-1.79, 2.05^2, 5)$<br>Median = 14%<br>IQR = 5% to 36%<br>95% range = 0.3% to 91%<br>$Pr(I^2 < 5\%) = 0.258$<br>$N = 7$ | $t(-1.27, 2.05^2, 5)$<br>Median = 22%<br>IQR = 8% to 48%<br>95% range = 0.4% to 95%<br>$Pr(I^2 < 5\%) = 0.174$<br>$N = 12$ | $t(-0.49, 2.06^2, 5)$<br>Median = 38%<br>IQR = 15% to 68%<br>95% range = 1% to 97%<br>$Pr(I^2 < 5\%) = 0.101$<br>$N = 80$ | $t(-1.04, 2.052, 5)$<br>Median = 26%<br>IQR = 10% to 55%<br>95% range = 0.6% to 95%<br>$Pr(I^2 < 5\%) = 0.149$<br>$N = 100$ | $t(-0.52, 2.04^2, 5)$<br>Median = 37%<br>IQR = 16% to 66%<br>95% range = 0.9% to 97%<br>$Pr(I^2 < 5\%) = 0.100$<br>$N = 19$ |
| **Mental health outcome** | $t(-1.34, 1.77^2, 5)$<br>Median = 21%<br>IQR = 9% to 42%<br>95% range = 0.7% to 90%<br>$Pr(I^2 < 5\%) = 0.155$<br>$N = 0$ | $t(-1.89, 1.70^2, 5)$<br>Median = 13%<br>IQR = 5% to 29%<br>95% range = 0.5% to 81%<br>$Pr(I^2 < 5\%) = 0.230$<br>$N = 0$ | $t(-1.37, 1.73^2, 5)$<br>Median = 20%<br>IQR = 9% to 41%<br>95% range = 0.8% to 90%<br>$Pr(I^2 < 5\%) = 0.151$<br>$N = 1$ | $t(-0.59, 1.77^2, 5)$<br>Median = 36%<br>IQR = 17% to 61%<br>95% range = 2% to 95%<br>$Pr(I^2 < 5\%) = 0.078$<br>$N = 0$ | $t(-1.14, 1.70^2, 5)$<br>Median = 24%<br>IQR = 11% to 46%<br>95% range = 1% to 90%<br>$Pr(I^2 < 5\%) = 0.116$<br>$N = 0$ | $t(-0.62, 1.73^2, 5)$<br>Median = 35%<br>IQR = 16% to 59%<br>95% range = 2% to 95%<br>$Pr(I^2 < 5\%) = 0.075$<br>$N = 3$ |
| **Biological-marker** | $t(-1.67, 2.49^2, 5)$<br>Median = 16%<br>IQR = 4% to 46%<br>95% range = 0.1% to 97%<br>$Pr(I^2 < 5\%) = 0.286$<br>$N = 11$ | $t(-2.22, 2.42^2, 5)$<br>Median = 10%<br>IQR = 3% to 31%<br>95% range = 0.08% to 93%<br>$Pr(I^2 < 5\%) = 0.365$<br>$N = 4$ | $t(-1.70, 2.47^2, 5)$<br>Median = 15%<br>IQR = 4% to 43%<br>95% range = 0.1% to 96%<br>$Pr(I^2 < 5\%) = 0.291$<br>$N = 15$ | $t(-0.92, 2.49^2, 5)$<br>Median = 28%<br>IQR = 8% to 64%<br>95% range = 0.3% to 98%<br>$Pr(I^2 < 5\%) = 0.187$<br>$N = 11$ | $t(-1.47, 2.42^2, 5)$<br>Median = 19%<br>IQR = 5% to 48%<br>95% range = 0.2% to 97%<br>$Pr(I^2 < 5\%) = 0.240$<br>$N = 10$ | $t(-0.95, 2.46^2, 5)$<br>Median = 27%<br>IQR = 8% to 62%<br>95% range = 0.3% to 98%<br>$Pr(I^2 < 5\%) = 0.184$<br>$N = 6$ |
| **Various subjectively measured outcomes** | $t(-0.66, 2.28^2, 5)$<br>Median = 35%<br>IQR = 11% to 66%<br>95% range = 0.6% to 98%<br>$Pr(I^2 < 5\%) = 0.131$<br>$N = 9$ | $t(-1.21, 2.28^2, 5)$<br>Median = 24%<br>IQR = 7% to 52%<br>95% range = 0.3% to 95%<br>$Pr(I^2 < 5\%) = 0.183$<br>$N = 2$ | $t(-0.70, 2.24^2, 5)$<br>Median = 34%<br>IQR = 12% to 65%<br>95% range = 0.6% to 97%<br>$Pr(I^2 < 5\%) = 0.131$<br>$N = 3$ | $t(0.108, 2.27^2, 5)$<br>Median = 53%<br>IQR = 22% to 80%<br>95% range = 1% to 99%<br>$Pr(I^2 < 5\%) = 0.078$<br>$N = 21$ | $t(-0.47, 2.20^2, 5)$<br>Median = 40%<br>IQR = 15% to 70%<br>95% range = 0.7% to 98%<br>$Pr(I^2 < 5\%) = 0.105$<br>$N = 13$ | $t(0.05, 2.24^2, 5)$<br>Median = 52%<br>IQR = 22% to 80%<br>95% range = 1% to 99%<br>$Pr(I^2 < 5\%) = 0.076$<br>$N = 4$ |

Table A9: Continuous outcome data: Predictive distributions for logit($I^2$) in future meta-analyses specializing in respiratory diseases, performed on the mean difference scale. Summary statistics are for $I^2$ on the untransformed scale. A $t(\mu, \sigma^2, 5)$ distribution represents a $t$-distribution with location $\mu$, scale $\sigma$ and 5 degrees of freedom. $N$ denotes the total number of meta-analyses contributing in each category.

| | Mean study size < 50 participants | | | Mean study size ≥ 50 participants | | |
|---|---|---|---|---|---|---|
| | **Pharmacological Vs. Placebo/ Control** | **Pharmacological Vs. Pharmacological** | **Non-Pharmacological (Any)** | **Pharmacological Vs. Placebo/ Control** | **Pharmacological Vs. Pharmacological** | **Non-Pharmacological (Any)** |
| **Obstetric outcome** | $t(0.33, 1.91^2, 5)$<br>Median = 58%<br>IQR = 30% to 82%<br>95% range = 3% to 98%<br>$Pr(I^2 < 5\%) = 0.041$<br>$N = 3$ | $t(-0.22, 1.84^2, 5)$<br>Median = 45%<br>IQR = 21% to 71%<br>95% range = 2% to 97%<br>$Pr(I^2 < 5\%) = 0.060$<br>$N = 7$ | $t(0.29, 1.86^2, 5)$<br>Median = 57%<br>IQR = 29% to 81%<br>95% range = 3% to 98%<br>$Pr(I^2 < 5\%) = 0.039$<br>$N = 8$ | $t(1.08, 1.91^2, 5)$<br>Median = 74%<br>IQR = 48% to 91%<br>95% range = 6% to 99%<br>$Pr(I^2 < 5\%) = 0.020$<br>$N = 0$ | $t(0.52, 1.84^2, 5)$<br>Median = 63%<br>IQR = 36% to 83%<br>95% range = 4% to 99%<br>$Pr(I^2 < 5\%) = 0.032$<br>$N = 0$ | $t(1.04, 1.85^2, 5)$<br>Median = 74%<br>IQR = 48% to 90%<br>95% range = 6% to 99%<br>$Pr(I^2 < 5\%) = 0.022$<br>$N = 0$ |
| **Resource use &<br>hospital stay/process** | $t(0.76, 2.60^2, 5)$<br>Median = 69%<br>IQR = 30% to 91%<br>95% range = 1% to 99.8%<br>$Pr(I^2 < 5\%) = 0.072$<br>$N = 1$ | $t(0.21, 2.55^2, 5)$<br>Median = 56%<br>IQR = 21% to 85%<br>95% range = 0.6% to 99.5%<br>$Pr(I^2 < 5\%) = 0.094$<br>$N = 0$ | $t(0.72, 2.61^2, 5)$<br>Median = 67%<br>IQR = 31% to 91%<br>95% range = 1% to 99.7%<br>$Pr(I^2 < 5\%) = 0.072$<br>$N = 0$ | $t(1.51, 2.60^2, 5)$<br>Median = 82%<br>IQR = 47% to 96%<br>95% range = 2% to 99.9%<br>$Pr(I^2 < 5\%) = 0.042$<br>$N = 1$ | $t(0.96, 2.55^2, 5)$<br>Median = 72%<br>IQR = 37% to 92%<br>95% range = 1% to 99.8%<br>$Pr(I^2 < 5\%) = 0.059$<br>$N = 0$ | $t(1.47, 2.61^2, 5)$<br>Median = 81%<br>IQR = 49% to 95%<br>95% range = 2% to 99.9%<br>$Pr(I^2 < 5\%) = 0.044$<br>$N = 6$ |
| **Internal &<br>External structure<br>related outcome** | $t(1.23, 2.15^2, 5)$<br>Median = 77%<br>IQR = 48% to 93%<br>95% range = 4% to 99.6%<br>$Pr(I^2 < 5\%) = 0.029$<br>$N = 0$ | $t(0.68, 2.14^2, 5)$<br>Median = 67%<br>IQR = 34% to 88%<br>95% range = 3% to 99%<br>$Pr(I^2 < 5\%) = 0.044$<br>$N = 0$ | $t(1.19, 2.16^2, 5)$<br>Median = 77%<br>IQR = 47% to 93%<br>95% range = 4% to 99.6%<br>$Pr(I^2 < 5\%) = 0.033$<br>$N = 0$ | $t(1.98, 2.15^2, 5)$<br>Median = 88%<br>IQR = 66% to 97%<br>95% range = 7% to 99.8%<br>$Pr(I^2 < 5\%) = 0.018$<br>$N = 0$ | $t(1.42, 2.14^2, 5)$<br>Median = 81%<br>IQR = 52% to 94%<br>95% range = 5% to 99.6%<br>$Pr(I^2 < 5\%) = 0.023$<br>$N = 0$ | $t(1.94, 2.15^2, 5)$<br>Median = 88%<br>IQR = 65% to 96%<br>95% range = 7% to 99.8%<br>$Pr(I^2 < 5\%) = 0.017$<br>$N = 0$ |
| **General physical health &<br>Adverse event &<br>Pain &<br>Quality of life/functioning** | $t(0.65, 2.21^2, 5)$<br>Median = 66%<br>IQR = 32% to 88%<br>95% range = 2% to 99%<br>$Pr(I^2 < 5\%) = 0.049$<br>$N = 0$ | $t(0.10, 2.19^2, 5)$<br>Median = 53%<br>IQR = 22% to 81%<br>95% range = 1% to 99%<br>$Pr(I^2 < 5\%) = 0.076$<br>$N = 0$ | $t(0.61, 2.22^2, 5)$<br>Median = 65%<br>IQR = 32% to 88%<br>95% range = 2% to 99%<br>$Pr(I^2 < 5\%) = 0.053$<br>$N = 2$ | $t(1.39, 2.21^2, 5)$<br>Median = 80%<br>IQR = 50% to 94%<br>95% range = 5% to 99.7%<br>$Pr(I^2 < 5\%) = 0.027$<br>$N = 1$ | $t(0.84, 2.19^2, 5)$<br>Median = 70%<br>IQR = 38% to 90%<br>95% range = 3% to 99%<br>$Pr(I^2 < 5\%) = 0.043$<br>$N = 0$ | $t(1.36, 2.22^2, 5)$<br>Median = 80%<br>IQR = 50% to 94%<br>95% range = 4% to 99.7%<br>$Pr(I^2 < 5\%) = 0.029$<br>$N = 2$ |
| **Signs/symptoms reflecting<br>continuation/end of condition<br>& Infection/onset of new<br>acute/chronic disease** | $t(0.91, 2.15^2, 5)$<br>Median = 70%<br>IQR = 40% to 90%<br>95% range = 4% to 99%<br>$Pr(I^2 < 5\%) = 0.033$<br>$N = 0$ | $t(0.36, 2.14^2, 5)$<br>Median = 59%<br>IQR = 29% to 84%<br>95% range = 2% to 99%<br>$Pr(I^2 < 5\%) = 0.055$<br>$N = 0$ | $t(0.87, 2.13^2, 5)$<br>Median = 71%<br>IQR = 40% to 90%<br>95% range = 3% to 99%<br>$Pr(I^2 < 5\%) = 0.038$<br>$N = 0$ | $t(1.66, 2.15^2, 5)$<br>Median = 83%<br>IQR = 59% to 95%<br>95% range = 7% to 99.7%<br>$Pr(I^2 < 5\%) = 0.019$<br>$N = 0$ | $t(1.11, 2.14^2, 5)$<br>Median = 75%<br>IQR = 46% to 92%<br>95% range = 4% to 99.5%<br>$Pr(I^2 < 5\%) = 0.031$<br>$N = 0$ | $t(1.62, 2.13^2, 5)$<br>Median = 84%<br>IQR = 59% to 95%<br>95% range = 6% to 99.7%<br>$Pr(I^2 < 5\%) = 0.021$<br>$N = 1$ |
| **Mental health outcome** | $t(0.81, 1.87^2, 5)$<br>Median = 69%<br>IQR = 43% to 87%<br>95% range = 5% to 99%<br>$Pr(I^2 < 5\%) = 0.023$<br>$N = 0$ | $t(0.26, 1.81^2, 5)$<br>Median = 56%<br>IQR = 30% to 80%<br>95% range = 4% to 98%<br>$Pr(I^2 < 5\%) = 0.033$<br>$N = 0$ | $t(0.77, 1.83^2, 5)$<br>Median = 68%<br>IQR = 42% to 86%<br>95% range = 5% to 99%<br>$Pr(I^2 < 5\%) = 0.024$<br>$N = 0$ | $t(1.56, 1.87^2, 5)$<br>Median = 83%<br>IQR = 61% to 93%<br>95% range = 10% to 99.5%<br>$Pr(I^2 < 5\%) = 0.011$<br>$N = 1$ | $t(1.01, 1.81^2, 5)$<br>Median = 73%<br>IQR = 48% to 89%<br>95% range = 7% to 99%<br>$Pr(I^2 < 5\%) = 0.018$<br>$N = 1$ | $t(-1.52, 1.83^2, 5)$<br>Median = 82%<br>IQR = 61% to 93%<br>95% range = 10% to 99%<br>$Pr(I^2 < 5\%) = 0.013$<br>$N = 3$ |
| **Biological-marker** | $t(0.48, 2.56^2, 5)$<br>Median = 61%<br>IQR = 24% to 89%<br>95% range = 1% to 99.6%<br>$Pr(I^2 < 5\%) = 0.079$<br>$N = 1$ | $t(-0.07, 2.50^2, 5)$<br>Median = 48%<br>IQR = 17% to 80%<br>95% range = 0.6% to 99%<br>$Pr(I^2 < 5\%) = 0.106$<br>$N = 0$ | $t(0.44, 2.54^2, 5)$<br>Median = 61%<br>IQR = 25% to 88%<br>95% range = 1% to 99.6%<br>$Pr(I^2 < 5\%) = 0.075$<br>$N = 0$ | $t(1.23, 2.56^2, 5)$<br>Median = 77%<br>IQR = 40% to 94%<br>95% range = 2% to 99.8%<br>$Pr(I^2 < 5\%) = 0.046$<br>$N = 0$ | $t(0.68, 2.50^2, 5)$<br>Median = 67%<br>IQR = 31% to 89%<br>95% range = 1% to 99.7%<br>$Pr(I^2 < 5\%) = 0.063$<br>$N = 0$ | $t(1.19, 2.54^2, 5)$<br>Median = 76%<br>IQR = 41% to 94%<br>95% range = 2% to 99.8%<br>$Pr(I^2 < 5\%) = 0.046$<br>$N = 0$ |
| **Various subjectively<br>measured outcomes** | $t(1.48, 2.36^2, 5)$<br>Median = 81%<br>IQR = 51% to 95%<br>95% range = 4% to 99.8%<br>$Pr(I^2 < 5\%) = 0.029$<br>$N = 0$ | $t(0.93, 2.29^2, 5)$<br>Median = 72%<br>IQR = 39% to 91%<br>95% range = 3% to 99.5%<br>$Pr(I^2 < 5\%) = 0.041$<br>$N = 0$ | $t(1.45, 2.33^2, 5)$<br>Median = 81%<br>IQR = 51% to 95%<br>95% range = 4% to 99.8%<br>$Pr(I^2 < 5\%) = 0.029$<br>$N = 0$ | $t(2.23, 2.36^2, 5)$<br>Median = 90%<br>IQR = 70% to 97%<br>95% range = 9% to 99.9%<br>$Pr(I^2 < 5\%) = 0.015$<br>$N = 0$ | $t(1.68, 2.29^2, 5)$<br>Median = 85%<br>IQR = 58% to 96%<br>95% range = 5% to 99.8%<br>$Pr(I^2 < 5\%) = 0.024$<br>$N = 0$ | $t(2.19, 2.32^2, 5)$<br>Median = 90%<br>IQR = 69% to 97%<br>95% range = 8% to 99.9%<br>$Pr(I^2 < 5\%) = 0.017$<br>$N = 0$ |

Table A10: Continuous outcome data: Predictive distributions for logit($I^2$) in future meta-analyses specializing in cancer, performed on the mean difference scale. Summary statistics are for $I^2$ on the untransformed scale. A $t(\mu, \sigma^2, 5)$ distribution represents a $t$-distribution with location $\mu$, scale $\sigma$ and 5 degrees of freedom. $N$ denotes the total number of meta-analyses contributing in each category.

## A.5 Frequencies of meta-analyses within research settings

For each type of outcome data, we illustrate the frequencies of meta-analyses within research settings defined by the type of outcome, type of intervention comparison and mean study size. In each Figure, we use ‘pharmctrl’, ‘pharmpharm’ and ‘anynon’ to denote types of intervention comparison: pharmacological vs placebo/control, pharmacological vs pharmacological and non-pharmacological vs any intervention, respectively.

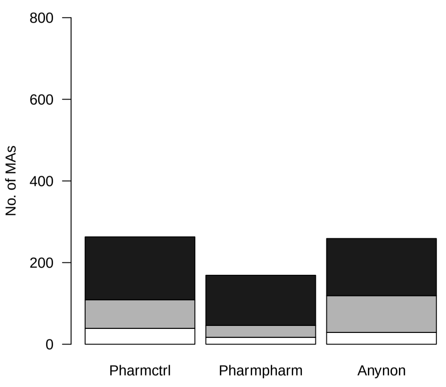


(a) Mean study size<50

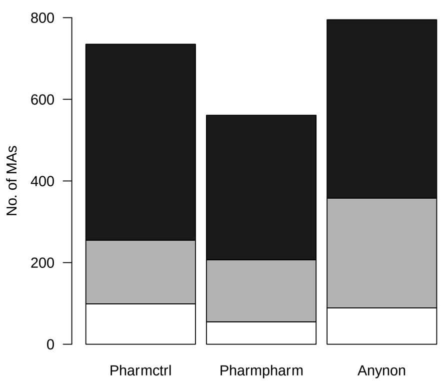


(b) Mean study size between 50 and 200

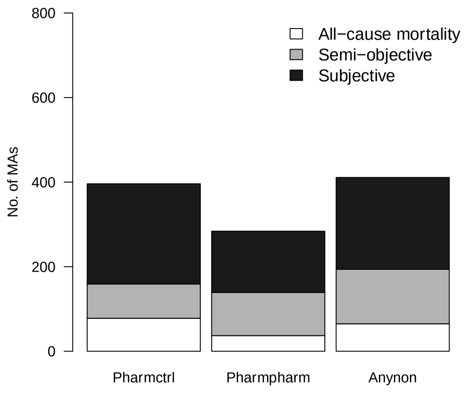


(c) Mean study size>50

Figure A5: Frequencies of binary outcome meta-analyses within research settings.

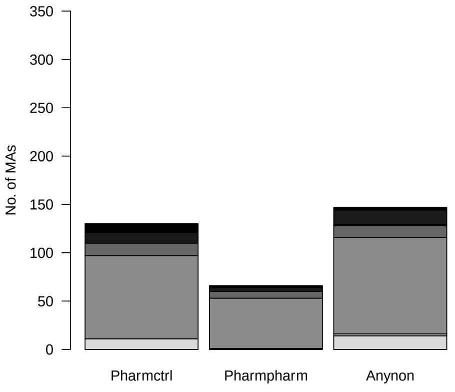


(a) Respiratory diseases

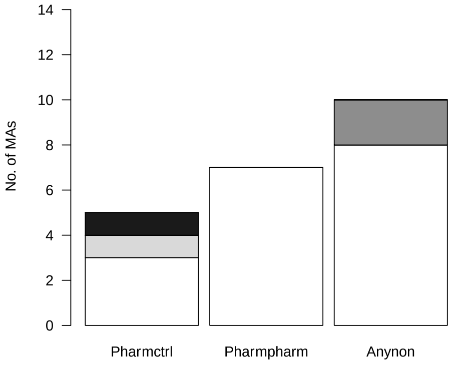


(b) Cancer

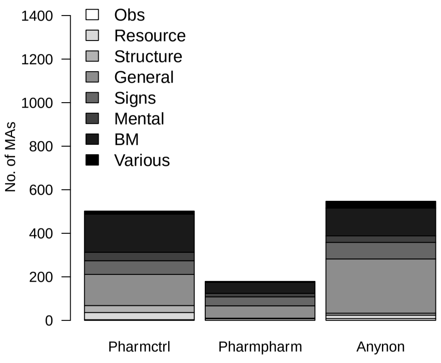


(c) Other

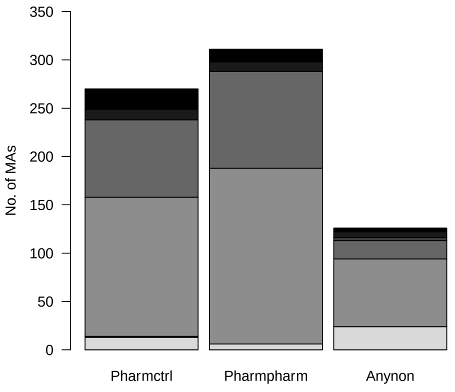


(d) Respiratory diseases

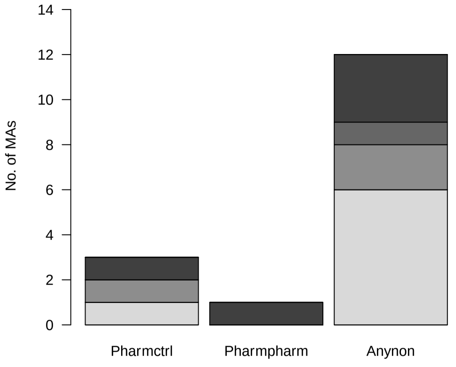


(e) Cancer

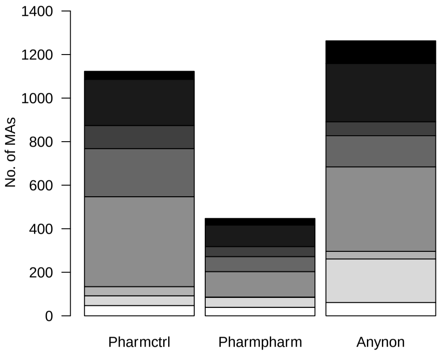


(f) Other

Figure A6: Frequencies of continuous outcome meta-analyses within research settings. Row 1: Mean study size<50. Row 2: Mean study size≥50.

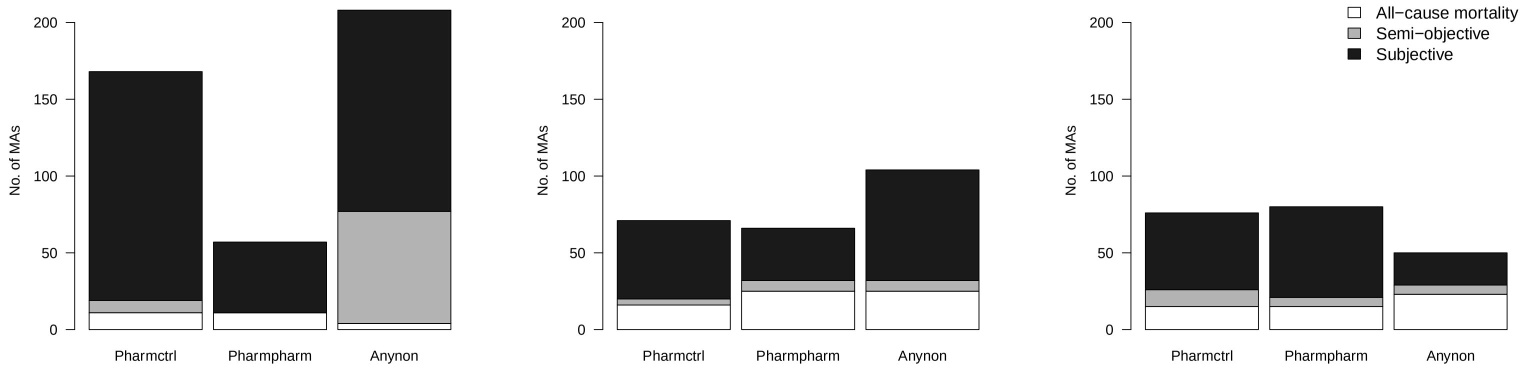


(a) Mean study size<50 (b) Mean study size between 50 and 200 (c) Mean study size>50

Figure A7: Frequencies of mixed outcome meta-analyses within research settings.

## A.6 WinBUGS code for the applications to example meta-analyses

We used the same random-effects models for a single meta-analysis as were used to obtain the predictive distributions for $I^2$ which may serve to inform priors for the between-study variance $\tau^2$. Note that 0.5 was added to all cells ($r_i^C$, $(n_i^C - r_i^C)$,$r_i^T$, $(n_i^T - r_i^T)$) in the $2 \times 2$ table where zeros caused problems with the calculation of the "typical" within-study variance `ssq`.

Bayesian binary outcome meta-analysis of radioisotopes data set (Roqué i Figuls *et al*, 2011), incorporating a predictive distribution for $I^2$ to provide an informative prior for $\tau^2$.

```
# Meta-analysis using the log odds ratio scale
#---------------------------------------------
model{
for(i in 1:K){

    #Perform random-effects meta-analysis
    #--------------------------------------
    r1[i] ~ dbin(p1[i],n1[i]) # Binomial distribution within studies (Control arm)
    r2[i] ~ dbin(p2[i],n2[i]) # Binomial distribution within studies (Treatment arm)
    v[i]<-1/r2[i]+1/r1[i]+1/(n2[i]-r2[i])+1/(n1[i]-r1[i])
    w[i]<-1/v[i]
    wsq[i]<-w[i]*w[i]
    alpha[i]<-logit(p1[i])+(theta[i]/2)
    logit(p2[i])<-alpha[i]+(theta[i]/2)
    p1[i]~dunif(0,1) # Vague prior for underlying probabilities of events
    theta[i] ~ dnorm(mu,invtausq) # Random-effects meta-analysis model
    }

    mu~dnorm(0,0.000001) # Vague prior for summary intervention effect (log OR scale)

    #Implement the informative prior for logit(I^2)
    #------------------------------------------------
    invtausq<-1/tausq
    W<-sum(w[])
    Wsq<-sum(wsq[])
    ssq<-(W*(K-1))/((W*W)-Wsq) # A fixed "typical" within-study variance
    tausq<-ssq*isq/(1-isq)
    isq<-exp(logit.isq)/(1+exp(logit.isq))
    prior.prec<-1/(0.82*0.82)
    logit.isq~dt(-1.14,prior.prec,5)
        # t(5df) prior with location parameter -1.14 and scale parameter 0.82
        for logit(I^2)
}
```

```
# Meta-analysis using the log relative risk scale
#---------------------------------------------------
model{
for(i in 1:K){

    #Perform random-effects meta-analysis
    #-------------------------------------
    r1[i] ~ dbin(p1[i],n1[i]) # Binomial distribution within studies (Control arm)
    r2[i] ~ dbin(p2[i],n2[i] # Binomial distribution within studies (Treatment arm)
    v[i]<-1/r2[i]+1/r1[i]-1/n2[i]-1/n1[i]
    w[i]<-1/v[i]
    wsq[i]<-w[i]*w[i]
    alpha[i]<-log(p1[i])+(thetaU[i]/2)
    log(p2[i])<-alpha[i]+(thetaU[i]/2)
    thetaU[i]<-min(theta[i],-log(p1[i])/C)
    p1[i]~dunif(0,1) #Vague prior for underlying probabilities of events
    theta[i] ~ dnorm(mu,invtausq)    # Random-effects meta-analysis model
      }

    mu~dnorm(0,0.000001) # Vague prior for summary intervention effect (log RR scale)
    C<-1.0000001

    #Implement the informative prior for logit(I^2)
    #-----------------------------------------------------
    invtausq<-1/tausq
    W<-sum(w[])
    Wsq<-sum(wsq[])
    ssq<-(W*(K-1))/((W*W)-Wsq) # A fixed "typical" within-study variance
    tausq<-ssq*isq/(1-isq)
    isq<-exp(logit.isq)/(1+exp(logit.isq))
    prior.prec<-1/(1.02*1.02)
    logit.isq~dt(-1.48,prior.prec,5)
        # t(5df) prior with location parameter -1.48 and scale parameter 1.02
        for logit(I^2)
    }
```

```
# Meta-analysis using the risk difference scale
#-------------------------------------------------
model{
for(i in 1:K){

    #Perform random-effects meta-analysis
    #-------------------------------------
    r1[i] ~ dbin(p1[i],n1[i])# Binomial distribution within studies (Control arm)
    r2[i] ~ dbin(p2[i],n2[i]) # Binomial distribution within studies (Treatment arm)
    v[i]<-((r2[i]*(n2[i]-r2[i]))/(n2[i]*n2[i]*n2[i]))
         +((r1[i]*(n1[i]-r1[i]))/(n1[i]*n1[i]*n1[i]))
    w[i]<-1/v[i]
    wsq[i]<-w[i]*w[i]
    alpha[i]<-p1[i]+((min(max(theta[i],-p1[i]),(1-p1[i])))/2)
    p2[i]<-alpha[i]+((min(max(theta[i],-p1[i]),(1-p1[i])))/2)
    p1[i]~dunif(0,1) # Vague prior for underlying probabilities of events
    theta[i] ~ dnorm(mu,invtausq) # Random-effects meta-analysis model
    }

    mu~dunif(-1,1) # Vague prior for summary intervention effect (RD scale)

    #Implement the informative prior for logit(I^2)
    #----------------------------------------------------
    invtausq<-1/tausq
    W<-sum(w[])
    Wsq<-sum(wsq[])
    ssq<-(W*(K-1))/((W*W)-Wsq) # A fixed "typical" within-study variance
    tausq<-ssq*isq/(1-isq)
    isq<-exp(logit.isq)/(1+exp(logit.isq))
    prior.prec<-1/(1.21*1.21)
    logit.isq~dt(-0.43,prior.prec,5)
        # t(5df) prior with location parameter -0.43 and scale parameter 1.21
        for logit(I^2)
}

# Radioisotopes data for 5 studies:
list(r2=c(8,7,28,5,16),r1=c(4,11,13,1,19),n2=c(12,19,43,6,36),n1=c(14,22,36,7,34),K=5)
```

Bayesian continuous outcome meta-analysis of schizophrenia data set (Li *et al*, 2009), assigning an informative prior for $I^2$ (and hence an informative prior for the between-study variance $\tau^2$).

```
# Meta-analysis using the SMD scale
#-----------------------------------
model{
for(i in 1:K){

     #Perform random-effects meta-analysis
     #-------------------------------------
     MD[i]<-treat_mean[i]-ctrl_mean[i]
      s[i]<-sqrt((((nT[i]-1)*treat_sd[i]*treat_sd[i])
               +((nC[i]-1)*ctrl_sd[i]*ctrl_sd[i]))/(N[i]-2))
     N[i]<-nC[i]+nT[i]
      y[i]<-(MD[i]/s[i])*(1-(3/(4*N[i]-9)))
     v[i]<-(N[i]/(nC[i]*nT[i]))+((y[i]*y[i])/(2*(N[i]-3.94)))
     prec.y[i]<-1/v[i]
     w[i]<-prec.y[i]
     wsq[i]<-w[i]*w[i]
     y[i]~dnorm(theta[i],prec.y[i]) # Assume normality for observed SMDs
     theta[i]~dnorm(mu,invtausq) # Random-effects meta-analysis model
     }

     mu~dnorm(0,0.000001) # Vague prior for summary intervention effect (SMD scale)

     #Implement the informative prior for logit(I^2)
     #----------------------------------------------------
     invtausq<-1/tausq
     W<-sum(w[])
     Wsq<-sum(wsq[])
     ssq<-(W*(K-1))/((W*W)-Wsq) # A fixed "typical" within-study variance
     tausq<-ssq*isq/(1-isq)
     isq<-exp(logit.isq)/(1+exp(logit.isq))
     prior.prec<-1/(1.70*1.70)
     logit.isq~dt( 0.25,prior.prec,5)
             # t(5df) prior with location parameter 0.25 and scale parameter 1.70
             for logit(I^2)
     }
```

```
# Meta-analysis using the mean difference (MD) scale
#----------------------------------------------------
model{
for(i in 1:K){

     #Perform random-effects meta-analysis
     #--------------------------------------
     y[i]<-treat_mean[i]-ctrl_mean[i]
     s[i]<-sqrt(((treat_sd[i]*treat_sd[i])/nT[i])+((ctrl_sd[i]*ctrl_sd[i])/nC[i]))
     v[i]<-s[i]*s[i]
     prec.y[i]<-1/v[i]
     w[i]<-prec.y[i]
       wsq[i]<-w[i]*w[i]
     y[i]~dnorm(theta[i],prec.y[i]) # Assume normality for observed MDs
     theta[i]~dnorm(mu,invtausq) # Random-effects meta-analysis model
     }

     mu~dnorm(0,0.000001) # Vague prior for summary intervention effect (MD scale)

     #Implement the informative prior for logit(I^2)
     #-----------------------------------------------------
     invtausq<-1/tausq
     W<-sum(w[])
     Wsq<-sum(wsq[])
     ssq<-(W*(K-1))/((W*W)-Wsq) # A "typical" within-study variance
     tausq<-ssq*isq/(1-isq)
     isq<-exp(logit.isq)/(1+exp(logit.isq))
     prior.prec<-1/(1.69*1.69)
     logit.isq~dt(-0.05,prior.prec,5)
             # t(5df) prior with location parameter -0.05 and scale parameter 1.69
             for logit(I^2)

}

# Schizophrenia data for four studies
list(K=4,nT=c(62,62,62,20), nC=c(62,62,62,20), treat_mean=c(59.4,9.5,21.3,39.2),
 ctrl_mean=c(47,8.6,15.9,41.9),treat_sd=c(17.2,2.6,7.2,17.6),ctrl_sd=c(6.9,0.9,3,19))
```

# References


[1] Chung Y, Rabe-Hesketh S, Choi I-H. 2013. Avoiding zero between-study variance estimates in random-effects meta-analysis. *Statistics in Medicine*, **32**: 4071-4089.

[2] Gart JJ, Pettigrew HM, Thomas DG. 1985. The effect of bias, variance estimation, skewness, and kurtosis of the empirical logit on weighted least squares analysis. *Biometrika* **72**:179-190.

[3] Hamza TH, Van Houwelingen HC, Stijnen T. 2008. The binomial distribution of meta-analysis was preferred to model within-study variability. *Journal of Clinical Epidemiology* **61**:41-51.

[4] Li C, Xia J, Wang J. 2009. Risperidone dose for schizophrenia. *Cochrane Database of Systematic Reviews* Issue 4. Art. No.:CD007474.

[5] Roqué i Figuls M, Martinez-Zapata MJ, Scott-Brown M, Alonso-Coello P. 2011. Radioisotopes for metastatic bone pain. *Cochrane Database of Systematic Reviews* Issue 7. Art. No.:CD003347.
